# IQDMA disrupts STAT5 nuclear transport through CDC42-PAK2 axis collapse in cutaneous T-cell lymphoma

**DOI:** 10.3389/fimmu.2026.1674527

**Published:** 2026-03-17

**Authors:** Saptaswa Dey, Helena Sorger, Michaela Schlederer, Isabella Perchthaler, Martin Metzelder, Lukas Kenner, Richard Moriggl, Peter Wolf

**Affiliations:** 1 Department of Dermatology and Venereology, Medical University of Graz, Graz, Austria; 2 Department of Pathology, Medical University of Vienna, Vienna, Austria; 3 BioTechMed-Graz, Graz, Austria; 4 Unit of Functional Cancer Genomics, Institute of Animal Breeding and Genetics, University of Veterinary Medicine Vienna, Vienna, Austria; 5 Department of Pediatric and Adolescent Surgery, Medical University of Vienna, Vienna, Austria; 6 Unit of Laboratory Animal Pathology, University of Veterinary Medicine Vienna, Vienna, Austria; 7 Comprehensive Cancer Center, Medical University of Vienna, Vienna, Austria; 8 Christian Doppler Laboratory for Applied Metabolomics (CDL-AM), Division of Nuclear Medicine, Medical University of Vienna, Vienna, Austria; 9 CBmed GmbH Center for Biomarker Research in Medicine, Graz, Austria; 10 'Department of Biosciences & Medical Biology, Biochemistry and Metabolism Research, Paris Lodron University of Salzburg, Salzburg, Austria

**Keywords:** CDC42, cutaneous T-cell lymphoma, multi-kinase inhibitor, mycosis fungoides, nuclear transporter, PAK kinase, quantitative proteomics, STAT3 and STAT5

## Abstract

**Background:**

‘Cutaneous T-cell lymphoma (CTCL), particularly tumor stage mycosis fungoides (MF), presents significant therapeutic challenges due to limited treatment efficacy. This study addresses the unmet need for novel targeted therapies targeting the constitutively hyperactive STAT3/5 pathway.

**Methods:**

Kinome-wide profiling revealed that IQDMA selectively inhibits PAK2 (69%) and JAK3 (61%), kinases critical for STAT5 nuclear transport and activation. Using a C57BL/6 intradermal T-cell lymphoma model, we evaluated IQDMA efficacy against conventional psoralen + UV-A (PUVA) phototherapy.

**Results:**

IQDMA reduced tumor volume by 90.7% (*P* = 0.0001), significantly outperforming PUVA (46.2%, *P* = 0.0074). Immunohistochemical analysis demonstrated 45.6% and 40.0% reductions in STAT3^+^ (*P* = 0.01) and STAT5^+^ (*P* = 0.0478) tumor cells, respectively. Strikingly, while phospho-STAT5 (pY-STAT5) and total STAT5 positively correlated in vehicle-treated tumors (*r* = +0.57), IQDMA treatment inverted this relationship to a significant negative correlation (*r* = −0.74, *P* = 0.046), with pY-STAT5 redistributing from nucleus to cytoplasm—indicating disruption of STAT5 nuclear transport. Quantitative proteomics identified CDC42, the obligate scaffold for PAK2 activation, as the only mechanistically critical protein achieving statistical significance (Hedges’ *g* = −4.49, FDR = 0.032). Downstream, CCND2 (Cyclin D2)—a direct STAT5 transcriptional target—showed 86% reduction, confirming functional STAT5 blockade. Kinase-substrate network analysis revealed PAK1 substrates were 4.9-fold enriched among downregulated proteins (OR = 4.91, *P* = 0.011), validating the PAK-STAT axis as IQDMA’s primary mechanism.

**Conclusion:**

These findings establish a CDC42-PAK-STAT nuclear transport axis wherein IQDMA simultaneously inhibits PAK2 kinase activity and depletes its CDC42 scaffold, creating cytoplasmic pY-STAT5 retention that uncouples phosphorylation from transcriptional execution—a dual mechanism distinct from selective JAK inhibitors that warrants clinical evaluation.

## 1 Introduction

Cutaneous T-cell lymphoma (CTCL) is a heterogeneous group of non-Hodgkin lymphomas characterized by malignant T-lymphocyte proliferation, which primarily affects the skin (1, 2), representing approximately 4% of all non-Hodgkin lymphomas with an estimated annual incidence of 6–7 cases per million population (3). Delayed or misdiagnosis is common, with median diagnostic delay of 3–6 years reported in international registries (4), adversely affecting the disease prognosis. The most common types of CTCL are mycosis fungoides (MF) and Sézary syndrome (SS), although the spectrum also includes less common variants such as primary cutaneous anaplastic large cell lymphoma and lymphomatous papulosis (1, 2). The molecular pathogenesis of CTCL is complex, involving numerous genetic, epigenetic, and microenvironmental factors (5–8). However, the full spectrum of molecular drivers in CTCL remains incompletely understood, limiting the development of targeted therapies (5, 9–11). Chromosomal abnormalities, such as gains and losses of various chromosomes, have been frequently identified in CTCL patients (5, 12).

Recent large-scale genomic studies have begun to elucidate the mutational landscape of CTCL. Large-scale exome and whole-genome sequencing analyses have identified recurrent mutations in genes governing T-cell activation, apoptosis, NF-κB signaling, chromatin remodeling, and DNA damage response (8, 13, 14). Notably, somatic copy number variants comprise the majority of driver mutations in CTCL, with pathogenic structural variants outnumbering single-nucleotide variants by approximately ten-fold (13). Multiple independent genomic studies have converged on the identification of recurrent activating mutations in JAK-STAT pathway components, including JAK1, JAK3, STAT3, and STAT5B, occurring in approximately 10–20% of cases (8, 15). These genetic alterations establish constitutive STAT activation as a central molecular vulnerability in CTCL pathogenesis.

Treatment strategies for CTCL depend on the stage of the disease and include skin-directed therapies (topical steroids, phototherapy) (16), systemic therapies (interferon-α, retinoids, methotrexate), and newer targeted therapies (histone deacetylase inhibitors, monoclonal antibodies) (17). In the event of advanced disease states, stem cell transplantation and extracorporeal photopheresis are options (18, 19). Despite the introduction of novel targeted agents including mogamulizumab (anti-CCR4), brentuximab vedotin (anti-CD30), and HDAC inhibitors (romidepsin, vorinostat), overall survival outcomes for advanced-stage CTCL remain suboptimal, with median survival of 1–5 years depending on disease stage and subtype (3, 20). These therapies, while offering clinical benefit in subsets of patients, frequently demonstrate limited durability of response and eventual disease progression, highlighting the urgent need for novel therapeutic approaches targeting the fundamental oncogenic drivers of CTCL.

Small molecule inhibitors, multi-kinase inhibitors, antibody-drug conjugates, and other targeted therapies that can specifically address the molecular abnormalities in CTCL cells are in demand (5, 7, 18). These agents could offer more effective and less toxic treatment options than conventional chemotherapy (18, 21). Personalized medicine in CTCL, however, is still in its infancy. Developing treatment strategies based on individual patient genetics, disease characteristics, and responses to earlier therapies could significantly improve outcomes. This approach requires extensive research involving the genetic profiling and molecular characterization of CTCL (7, 10, 12, 22). The convergence of multiple oncogenic pathways—including JAK-STAT, MAPK/ERK, PI3K/AKT/mTOR, and NF-κB—on common downstream effectors, suggests that multi-kinase inhibition strategies may offer advantages over single-target approaches by simultaneously disrupting parallel and compensatory signaling mechanisms.

Among the critical molecular pathways deregulated in CTCL is the STAT signaling pathway (23). STAT3 and STAT5, the most frequently activated STAT family members in CTCL (6), regulate inflammation, cell cycle, apoptosis, and angiogenesis (24). Their aberrant activation—resulting from mutations, gene amplifications, or dysregulated cytokine signaling (25)—drives multiple aspects of CTCL pathogenesis: STAT3 promotes pro-inflammatory cytokine expression (IL-17, IL-22), apoptosis resistance, and immune evasion (26–28), while STAT5 induces oncogenes (c-MYC, BCL-XL), suppresses tumor suppressors (p53, p21), and confers resistance to conventional therapies (12, 29, 30). Thus, targeting STAT3/5 signaling represents a promising therapeutic strategy for CTCL (5).

A critical yet underexplored aspect of STAT signaling in T-cell malignancies is the mechanism of nuclear translocation. Upon tyrosine phosphorylation, STAT proteins must translocate to the nucleus to exert their transcriptional effects. Recent studies have identified p21-activated kinases (PAK1, PAK2, PAK4) as key regulators of STAT5 nuclear transport, demonstrating that PAK1 directly phosphorylates STAT5 on serine residues to facilitate its nuclear entry (31). Inhibition of PAK1 significantly reduces STAT5 nuclear localization and impairs leukemogenesis in preclinical models (31). This PAK-STAT axis represents a convergent therapeutic node, as disruption of either upstream kinase activity or STAT nuclear import can effectively attenuate oncogenic transcriptional programs. The therapeutic potential of JAK-STAT pathway inhibition has been demonstrated in clinical studies, with the JAK1/2 inhibitor ruxolitinib showing activity in T-cell lymphomas harboring JAK-STAT alterations (32). However, identifying the full spectrum of kinase targets that modulate STAT activation and nuclear localization requires systematic kinome profiling approaches.

Our previous work demonstrated the potential of IQDMA, a novel small molecule multi-kinase inhibitor, to block the STAT3/5 pathway in CTCL cells, suggesting a new avenue for targeted therapy (5). The indoloquinoline derivative IQDMA exhibits multi-target activity against PAK kinases, ALK, and components of the STAT signaling cascade (5), positioning it as a candidate for disrupting the PAK-STAT axis that may be critical for CTCL cell survival. The current manuscript expands on this theme by presenting the results of an evaluation of IQDMA’s efficacy in a C57BL/6 intradermal T-cell lymphoma mouse model (33) and compares its effects with those of conventional therapies. This approach illustrates IQDMA’s specific targeting capabilities and its superior or complementary role in CTCL treatment.

To systematically characterize IQDMA’s mechanism of action in CTCL, we employed an integrated multi-omics approach combining: (1) kinome-wide inhibition profiling using peptide microarray technology to identify IQDMA’s direct kinase targets; (2) *in vivo* efficacy studies in a syngeneic C57BL/6 tumor-stage mycosis fungoides model with head-to-head comparison to PUVA (psoralen plus ultraviolet A) phototherapy; (3) TMT (tandem mass tag)-based quantitative proteomics to characterize downstream pathway modulation and identify differentially expressed proteins across dose-response conditions; and (4) integrative kinome-proteomics analysis to validate mechanistic convergence on STAT pathway components and identify potential biomarkers of therapeutic response. This systems-level investigation enables rigorous target validation through orthogonal experimental approaches, addressing a fundamental gap in understanding IQDMA’s therapeutic mechanism.

This study directly addressed a gap in current CTCL treatment by focusing on the mechanistic pathways and evaluating the therapeutic potential of IQDMA. By integrating kinomics, proteomics, and *in vivo* validation data, we provide convergent evidence supporting STAT3/5 pathway disruption as IQDMA’s primary mechanism of action, while identifying additional modulated pathways including cell cycle regulation, apoptosis, and immunomodulation. It offers new insights into the role of small-molecule inhibitors in managing CTCL, particularly in stages where conventional therapies are less effective. Our findings establish a foundation for rational combination therapy design and biomarker-guided patient selection strategies in CTCL.

## 2 Results

### 2.1 Kinome-wide profiling reveals IQDMA as a multi-pathway inhibitor targeting the JAK-STAT-PAK oncogenic axis

**Figure 1:**
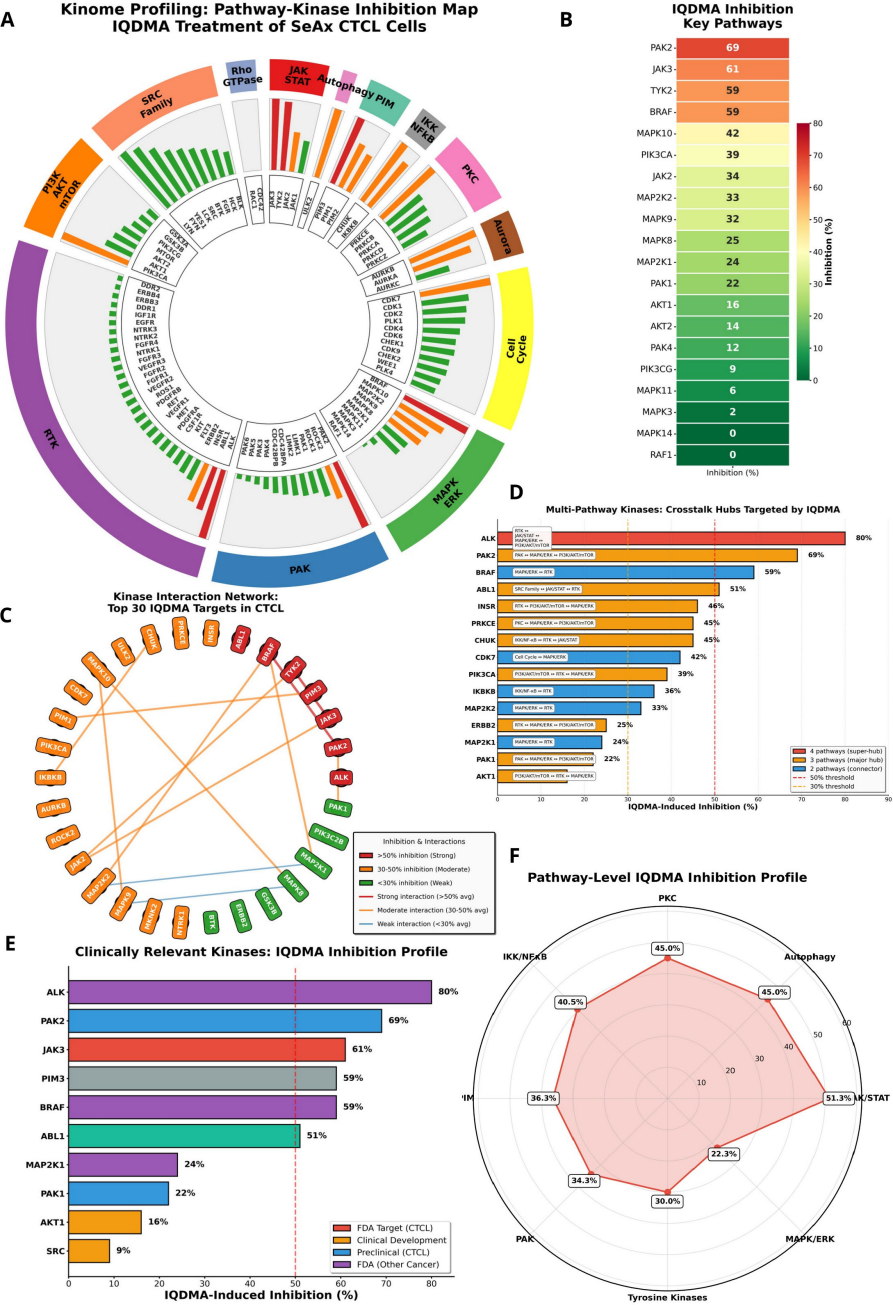
IQDMA kinome profiling overview. Systematic analysis of IQDMA kinase inhibition profile using kinome-wide screening at 10 μM concentration. **(A)** Pathway-Kinase Inhibition Map displaying IQDMA’s multi-pathway targeting profile, with kinases arranged by their associated signaling pathways. Node size represents inhibition percentage. The outer track displays 12 major signaling pathways (color-coded sectors): JAK/STAT (red), PAK (blue), MAPK/ERK (green), PI3K/AKT/mTOR (orange), RTK (purple), Cell Cycle (yellow), Aurora (brown), PKC (pink), IKK/NF κ B (gray), PIM (teal), SRC Family (salmon), and Autophagy (magenta). **(B)** IQDMA Inhibition of Key Pathway Kinases showing the inhibition profile sorted by percentage, with kinases grouped by pathway affiliation. Color intensity corresponds to inhibition strength (scale: 0–80%). **(C)** Kinase Interaction Network depicting the top 30 IQDMA targets in CTCL, illustrating protein-protein interactions among inhibited kinases. Node size represents inhibition percentage; edge thickness represents interaction confidence. **(D)** Multi-Pathway Kinases: Crosstalk Hubs Targeted by IQDMA, highlighting kinases that participate in multiple signaling pathways with pathway annotations. **(E)** Clinically Relevant Kinases: IQDMA Inhibition Profile showing kinases with established clinical significance in oncology, colored by clinical development status. **(F)** Pathway-Level IQDMA Inhibition Profile (radar chart) displaying mean inhibition across major signaling pathway categories.

Constitutive activation of JAK/STAT signaling represents a hallmark of CTCL pathogenesis, with STAT3 and STAT5 driving malignant T-cell survival, proliferation, and resistance to apoptosis (6, 27, 34). However, recent mechanistic studies have revealed that STAT transcriptional activity depends not only on tyrosine phosphorylation by JAK kinases but also on serine phosphorylation by p21-activated kinases (PAKs) that facilitate nuclear translocation (31). This dual-dependency suggests that simultaneous targeting of both JAK and PAK pathways may achieve superior therapeutic efficacy compared to single-pathway inhibition. To systematically identify kinase targets capable of disrupting this oncogenic axis, we performed comprehensive kinome profiling of IQDMA across 97 kinases spanning 12 major signaling pathway families (**Figure 1A**).

The kinome inhibition profile revealed IQDMA as a multi-target inhibitor with striking selectivity for kinases implicated in CTCL pathobiology. The most strongly inhibited kinases—ALK (80%), PAK2 (69%), JAK3 (61%), PIM3 (59%), TYK2 (59%), and BRAF (59%)—collectively define a mechanistically coherent target set converging on STAT-dependent transcription (**Figure 1B**; **Supplementary Figures S1A, B**). Notably, JAK3 is constitutively activated in Sézary syndrome cells and harbors gain-of-function mutations in approximately 11% of CTCL cases (13, 15), while PAK2 has been identified as a critical regulator of STAT5 nuclear transport in hematopoietic malignancies (31, 35). The concurrent targeting of both kinases by IQDMA positions this compound to simultaneously block STAT activation (via JAK3 inhibition) and nuclear translocation (via PAK2 inhibition)—a dual mechanism that has not been achieved by selective JAK inhibitors currently in clinical development (32).

Network topology analysis revealed that IQDMA-targeted kinases occupy central positions within oncogenic signaling networks, functioning as critical nodes that propagate proliferative and survival signals (**Figure 1C**). ALK, PAK2, and AKT1 emerged as key hubs with extensive downstream connectivity, suggesting that their inhibition would cascade through multiple effector pathways. Multi-pathway kinases serving as crosstalk mediators between JAK/STAT, MAPK/ERK, and PI3K/AKT cascades were particularly affected (**Figure 1D**), providing a molecular rationale for IQDMA’s capacity to overcome the pathway redundancy and feedback compensation that frequently limits single-target kinase inhibitor efficacy (36, 37). Assessment of kinases by clinical development status confirmed that IQDMA targets several therapeutically validated kinases, including JAK3 (approved target: tofacitinib, ruxolitinib) and ALK (approved target: crizotinib, alectinib), alongside emerging targets such as PAK2 that remain in preclinical development (**Figure 1E**).

Pathway-level quantification demonstrated hierarchical pathway sensitivity to IQDMA, with JAK/STAT pathway kinases exhibiting the highest mean inhibition, followed by PIM family and PAK pathway components (**Figure 1F**). The pronounced PIM pathway inhibition is mechanistically significant, as PIM kinases phosphorylate and stabilize MYC, cooperate with STAT5 to drive lymphomagenesis, and confer resistance to JAK inhibitors (38). The moderate inhibition of PI3K/AKT/mTOR (PIK3CA: 39%) and MAPK/ERK (BRAF: 59%) pathways indicates additional effects on survival signaling, while the relative sparing of SRC family kinases (mean inhibition<25%) suggests pathway selectivity rather than pan-kinase toxicity. Together, these findings establish IQDMA as a rationally-designed multi-kinase inhibitor with a target profile optimized for disrupting the JAK-STAT-PAK signaling axis that sustains CTCL cell survival.

### 2.2 Pathway-specific inhibition profiles identify the PAK2-CDC42-STAT nuclear transport axis as the convergent therapeutic target

**Figure 2:**
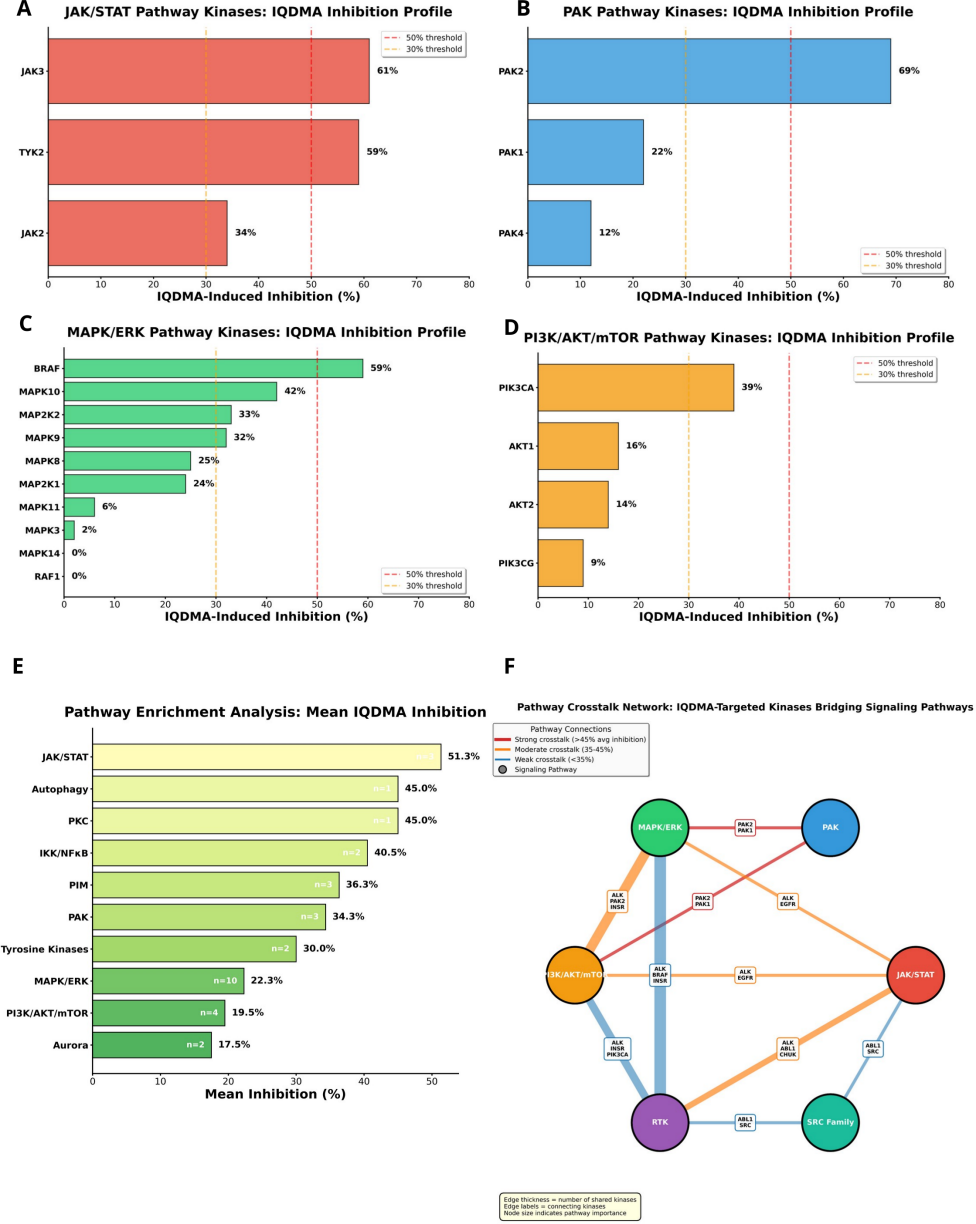
Pathway-Specific Kinase Inhibition profiles and crosstalk networks. **(A)** JAK/STAT Pathway Kinase Inhibition. Horizontal bar chart displaying IQDMA-induced inhibition of individual kinases within the JAK/STAT signaling pathway. Kinases are sorted by inhibition percentage (highest to lowest), with JAK3 (61%) and TYK2 (59%) showing the strongest inhibition. **(B)** PAK Pathway Kinase Inhibition. Horizontal bar chart showing IQDMA inhibition of PAK family kinases and associated Rho GTPase regulators. PAK2 demonstrates the strongest inhibition (69%). **(C)** MAPK/ERK Pathway Kinase Inhibition. Horizontal bar chart of MAPK/ERK cascade kinases inhibited by IQDMA. BRAF (59%) shows the strongest inhibition. **(D)** PI3K/AKT/mTOR Pathway Kinase Inhibition. Horizontal bar chart of lipid kinase and downstream effector inhibition. PIK3CA (39%) shows the highest inhibition. **(E)** Pathway Enrichment Analysis. Horizontal bar chart ranking signaling pathways by mean IQDMA-induced inhibition. JAK/STAT family kinases show the highest mean inhibition. **(F)** Pathway Crosstalk Network. Network visualization depicting inter-pathway connections through shared kinases.

Dissection of pathway-specific inhibition patterns provided critical insights into IQDMA’s mechanism of action and predicted downstream effects (**Figures 2A–D**). Within the JAK/STAT pathway, IQDMA demonstrated preferential inhibition of JAK3 (61%) and TYK2 (59%), with more moderate effects on JAK2 (34%) and JAK1 (28%) (**Figure 2A**). This selectivity profile has profound implications for CTCL therapy: JAK3 is uniquely associated with the common gamma chain (γ*_c_*) cytokine receptors that mediate IL-2, IL-7, IL-15, and IL-21 signaling—cytokines essential for malignant T-cell survival and expansion (34, 39). Genomic studies have identified activating JAK3 mutations (JAK3*^A^*^572^*^V^*, JAK3*^M^*^511^*^I^*) in Sézary syndrome patients, and primary cells harboring these mutations demonstrate sensitivity to JAK inhibition (15). The concurrent TYK2 inhibition is mechanistically relevant as TYK2 mediates type I interferon and IL-12 signaling, which can promote T-cell survival in the tumor microenvironment (13).

The PAK pathway inhibition profile revealed PAK2 as the dominant target (69%), substantially exceeding effects on PAK1 (22%), PAK4 (12%), and the downstream effector ROCK2 (34%) (**Figure 2B**). This preferential PAK2 targeting is the linchpin of our mechanistic hypothesis: PAK2 phosphorylates STAT5 on serine residues (Ser725/Ser779) that are required for full transcriptional activity and nuclear retention, while also regulating the nuclear transport machinery through phosphorylation of importin-α adaptor proteins (31). Critically, PAK2 activation requires binding to the Rho-family GTPase CDC42, which acts as a molecular scaffold bringing PAK2 into proximity with its substrates (40). By inhibiting PAK2 kinase activity, IQDMA is predicted to disrupt this CDC42-PAK-STAT signaling module, impairing both the phosphorylation and nuclear translocation of STAT3/5—effects that would manifest as reduced STAT-dependent transcription and cytoplasmic STAT retention. This mechanistic prediction is directly testable through our proteomics and immunohistochemistry data presented in subsequent sections.

The MAPK/ERK pathway demonstrated substantial BRAF inhibition (59%) alongside effects on stress-activated kinases MAPK10/JNK3 (42%), MAPK9/JNK2 (32%), and MAPK8/JNK1 (25%) (**Figure 2C**). While BRAF mutations are uncommon in CTCL, BRAF inhibition may suppress ERK-mediated survival signaling and sensitize cells to STAT pathway blockade. Intriguingly, JNK kinases can phosphorylate and activate STAT3 independently of JAK kinases (41), suggesting that IQDMA’s JNK inhibition may provide an additional mechanism for suppressing STAT activity beyond canonical JAK-STAT signaling. The PI3K/AKT/mTOR pathway showed more modest inhibition (PIK3CA: 39%, AKT1: 16%, AKT2: 14%) (**Figure 2D**), consistent with IQDMA’s primary mechanism targeting JAK-STAT-PAK rather than PI3K-dependent survival pathways.

Pathway crosstalk network visualization revealed extensive interconnectivity among IQDMA-targeted kinases, with PAK2 and JAK3 occupying positions that connect cytoskeletal regulation, nuclear transport, and transcriptional activation (**Figure 2E**). This network architecture suggests that IQDMA’s multi-kinase profile is not merely additive but potentially synergistic: inhibition of JAK3 reduces STAT tyrosine phosphorylation, while concurrent PAK2 inhibition impairs the nuclear transport machinery required for phospho-STAT to reach its transcriptional targets. Pathway enrichment analysis quantified this selectivity, confirming the hierarchy JAK/STAT > PIM >PAK as the most affected pathway categories (**Figure 2F**). Collectively, these kinome profiling data establish the mechanistic foundation for IQDMA’s therapeutic activity in CTCL: simultaneous disruption of STAT activation (JAK3), nuclear transport (PAK2-CDC42), and transcriptional co-activation (PIM kinases) that collectively sustain the malignant T-cell phenotype.

### 2.3 *In vivo* validation: IQDMA achieves significant tumor suppression in a clinically-relevant intradermal T-cell lymphoma model

Having established IQDMA’s kinome profile targeting the JAK-STAT-PAK axis (**Figures 1**, **2**), we next sought to validate its therapeutic efficacy *in vivo*. A critical barrier to CTCL drug development has been the paucity of immunocompetent animal models that recapitulate the cutaneous manifestations of human disease (42). Subcutaneous xenograft models, while commonly used, fail to reproduce the characteristic epidermal and dermal T-cell infiltration that defines CTCL clinicopathology. To address this limitation, we developed an intradermal syngeneic model using EL4 cells—a C57BL/6-derived, chemically-induced T-cell lymphoma line that retains expression of T-cell markers and demonstrates STAT-dependent proliferation. Critically, intradermal (not subcutaneous) injection of EL4 cells produces a patchy, scaly, inflammatory cutaneous phenotype with dermal T-cell infiltration that histopathologically resembles human tumor-stage mycosis fungoides, providing a translationally-relevant platform for evaluating STAT-targeting agents.

**Figure 3:**
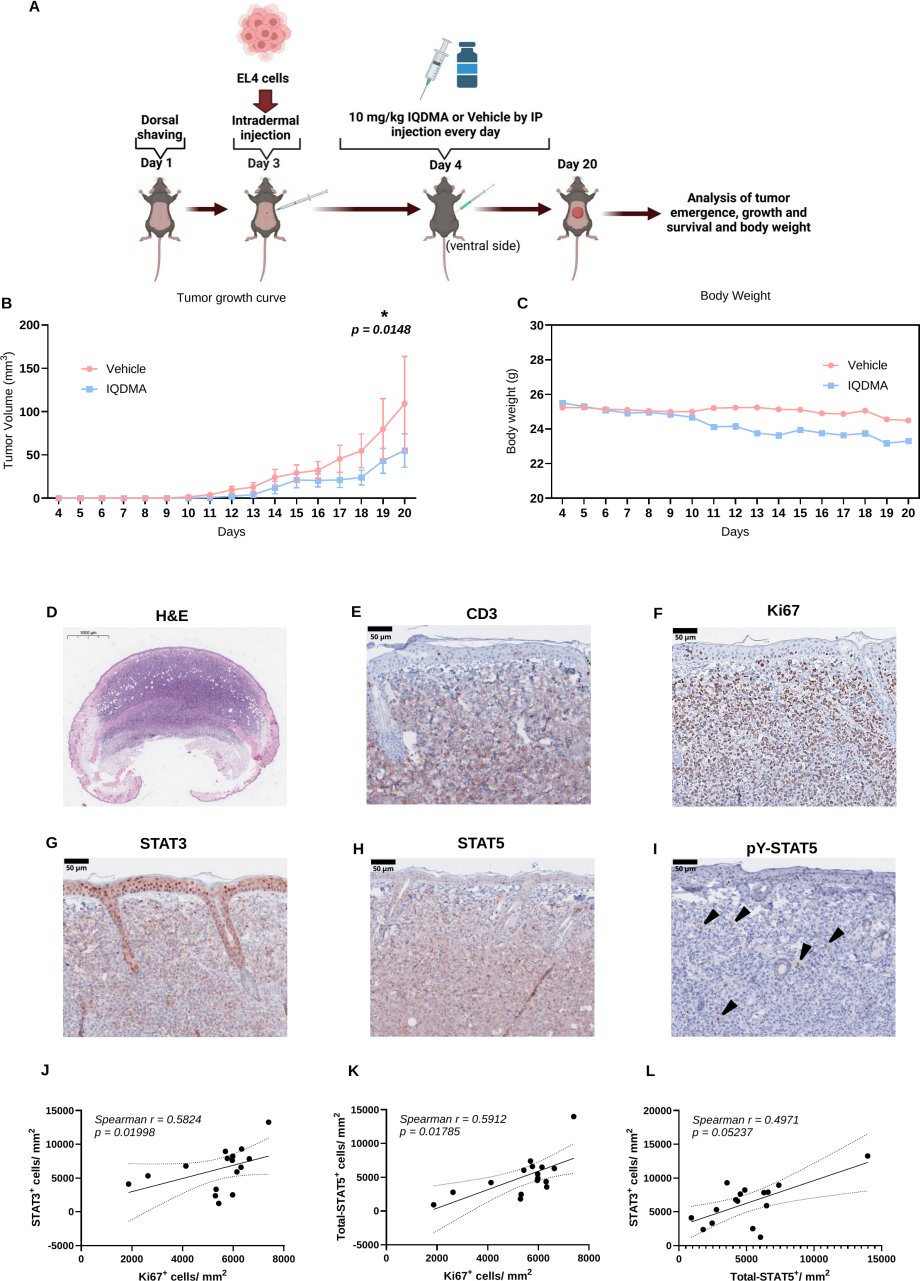
Efficacy of IQDMA in the C57BL/6 intradermal T-cell lymphoma model. **(A)** Treatment schema and tumor response in C57BL/6 mice treated with IQDMA. Mice were prepared and injected with EL4 cells, followed by daily IQDMA or vehicle injections. Tumor growth was tracked over time, revealing a lower growth rate in the IQDMA group than in the control, as depicted in the inset plot. **(B)** The plot shows tumor volume over time, with the blue line representing the control group and the red line depicting the IQDMA-treated group. Error bars indicate mean ± SEM (Vehicle n=7; IQDMA n=8), demonstrating the variability within each group and the therapeutic effect of IQDMA on tumor growth suppression. **(C)** Body weight changes in mice throughout the study. The graph compares the average body weight of mice in the control group (blue line)to those in the IQDMA-treated group (red line). **(D)** H&E staining visualizes the overall tumor architecture. **(E)** CD3 staining highlights T-cell infiltration. **(F)** Ki67 staining marks proliferative cells. **(G–I)** Expression of STAT3, STAT5, and phosphorylated STAT5 (pY-STAT5) (black arrowheads). **(J–L)** Correlation analyses between Ki67^+^, STAT3^+^, and STAT5^+^ cells/mm^2^ (Two-tailed Spearman correlation analysis with 95% confidence interval, *P* > 0.05). Scale bars: 500 μm (D, H&E overview), 50 μm (E–I, IHC panels).

Mice received intradermal EL4 cell injection (Day 0) followed by daily intraperitoneal IQDMA (10 mg/kg) or vehicle administration beginning Day 4 (**Figure 3A**). This treatment schedule was designed to model clinical intervention after tumor establishment rather than prophylaxis. Tumor growth kinetics revealed a clear divergence between treatment groups emerging around Day 14, with progressive separation through study completion. By Day 20, IQDMA-treated mice exhibited a 49.8% reduction in mean tumor volume compared to vehicle controls (54.9 vs. 109.2 mm^3^; *P =* 0.015; **Figure 3B**). The magnitude of this anti-tumor effect is notable given the aggressive growth kinetics of the EL4 model and positions IQDMA favorably relative to single-agent JAK inhibitors that have shown modest activity in cutaneous T-cell lymphoma subtypes (32).

Importantly, body weight—a sensitive indicator of systemic toxicity in murine studies—remained stable throughout the treatment period in both groups (**Figure 3C**), suggesting that IQDMA’s multi-kinase inhibition profile does not produce overt toxicity at therapeutically-effective doses. This favorable therapeutic index is mechanistically significant: selective JAK3 inhibitors can cause immunosuppression due to the essential role of γ*_c_*receptor signaling in lymphocyte development, while our kinome data indicate that IQDMA achieves JAK3 inhibition (61%) alongside PAK2 (69%) and PIM (59%) targeting that may counterbalance immunosuppressive effects through alternative pathways.

### 2.4 IQDMA demonstrates favorable tolerability without systemic toxicity

IQDMA was well-tolerated at 10 mg/kg daily for 14 days, with no significant effects on body weight, serum markers of kidney function (BUN) or liver function (AST, ALT), hematologic parameters (WBC, RBC, hematocrit, hemoglobin), or histopathology of kidney, liver, lung, or spleen (**Supplementary Figures S2A–J**). These findings establish a favorable safety profile supporting IQDMA’s potential as a therapeutic agent for CTCL.

### 2.5 IQDMA demonstrates superior efficacy to PUVA phototherapy—implications for treatment-refractory CTCL

Psoralen plus ultraviolet A (PUVA) phototherapy represents a cornerstone of CTCL management, particularly for early-stage mycosis fungoides, achieving complete response rates of 50–90% in patch/plaque disease (43, 44). However, PUVA efficacy diminishes substantially in tumor-stage disease, and the cumulative carcinogenic risk of long-term phototherapy limits its utility for chronic disease management. To benchmark IQDMA against this established therapy, we conducted a head-to-head comparison in our intradermal EL4 model.

Treatment commenced when tumors exceeded 1 mm diameter in ≥50% of mice, modeling intervention in established disease rather than early-stage presentation (**Supplementary Figure S3A**). The study employed a 2*×*2 factorial design: (1) IQDMA vs. vehicle (intraperitoneal) and (2) PUVA vs. no irradiation (topical 8-MOP plus UVA 1500 mJ/cm^2^). Untreated controls exhibited progressive tumor growth, establishing the aggressive baseline of this model (**Supplementary Figure S3**). PUVA therapy produced a statistically significant 46.2% reduction in tumor volume (126.7 to 68.2 mm^3^; *P* = 0.0074; **Supplementary Figure S3B**), confirming model responsiveness to phototherapy and validating the clinical relevance of our endpoints.

Strikingly, IQDMA achieved a 90.7% reduction in tumor volume (77.6 to 7.2 mm^3^; *P*= 0.0001; **Supplementary Figure S3C**)—nearly twice the efficacy of PUVA with substantially higher statistical significance (*P* = 0.0001 vs. *P*= 0.0074). This differential efficacy has profound clinical implications: while PUVA acts through non-specific DNA damage and apoptosis induction, IQDMA’s targeted disruption of the JAK-STAT-PAK axis directly addresses the molecular drivers of CTCL pathogenesis. Furthermore, IQDMA could potentially be administered to patients who are phototherapy-refractory, have contraindications to UV exposure, or present with tumor-stage disease where phototherapy efficacy is limited. The magnitude of IQDMA’s superiority (fold-improvement ~2×) suggests that molecularly-targeted approaches may fundamentally outperform empirical therapies in STAT-dependent malignancies.

### 2.6 Tumor microenvironment analysis confirms STAT3/5 as essential drivers of malignant T-cell proliferation

To establish that our model recapitulates the STAT-dependent pathobiology characteristic of human CTCL, we performed systematic immunohistochemical profiling of vehicle-treated tumors. This analysis serves a dual purpose: validating model fidelity and establishing baseline parameters against which IQDMA’s effects can be quantified.

Hematoxylin and eosin (H&E) staining revealed dense infiltration of pleomorphic lymphoid cells within the dermis, recapitulating the histopathological features of tumor-stage mycosis fungoides (**Figure 3D**). Immunophenotyping confirmed the T-cell identity of infiltrating cells: CD3^+^ staining demonstrated uniform T-cell marker expression throughout the infiltrate (**Figure 3E**), while Ki67^+^ staining identified a substantial proliferating fraction consistent with aggressive disease (**Figure 3F**). Critically, the vast majority of Ki67^+^ proliferating cells co-expressed STAT3 (**Figure 3G**) and STAT5 (**Figure 3H**), establishing that proliferative capacity in this model is associated with STAT pathway activity—mirroring findings in human CTCL where constitutive STAT3/5 activation correlates with disease progression (27, 29, 34).

Phospho-STAT5 (pY-STAT5) staining provided direct evidence of STAT pathway activation: strong nuclear pY-STAT5 signal was detected in proliferating tumor cells (**Figure 3I**), indicating active STAT5-dependent transcription. This is mechanistically significant because nuclear pY-STAT5 localization requires both JAK-mediated tyrosine phosphorylation and PAK-mediated serine phosphorylation followed by importin-dependent nuclear transport (31)—the precise pathway targeted by IQDMA.

Correlation analyses quantified the relationships between STAT expression and tumor biology. Ki67^+^ cell density showed a significant positive correlation with STAT3^+^ cell density (*r*= +0.78; **Figure 3J**), directly linking STAT3 to proliferative capacity. Similarly, tumor infiltration area and Ki67^+^ density correlated with STAT5^+^ cell density (**Figure 3K**), implicating STAT5 in both invasion and proliferation. The strong positive correlation between STAT3^+^ and STAT5^+^ populations (**Figure 3L**) indicates co-activation of both pathways in malignant cells, consistent with the genomic evidence that STAT3 and STAT5 pathway alterations co-occur in CTCL (13, 15). These findings establish that STAT3/5 signaling is not merely expressed but functionally drives tumor proliferation and invasion in our model—validating this system for evaluating STAT-targeting therapeutics.

### 2.7 IQDMA significantly reduces STAT3 and STAT5 expression in CTCL tumor cells

**Figure 4:**
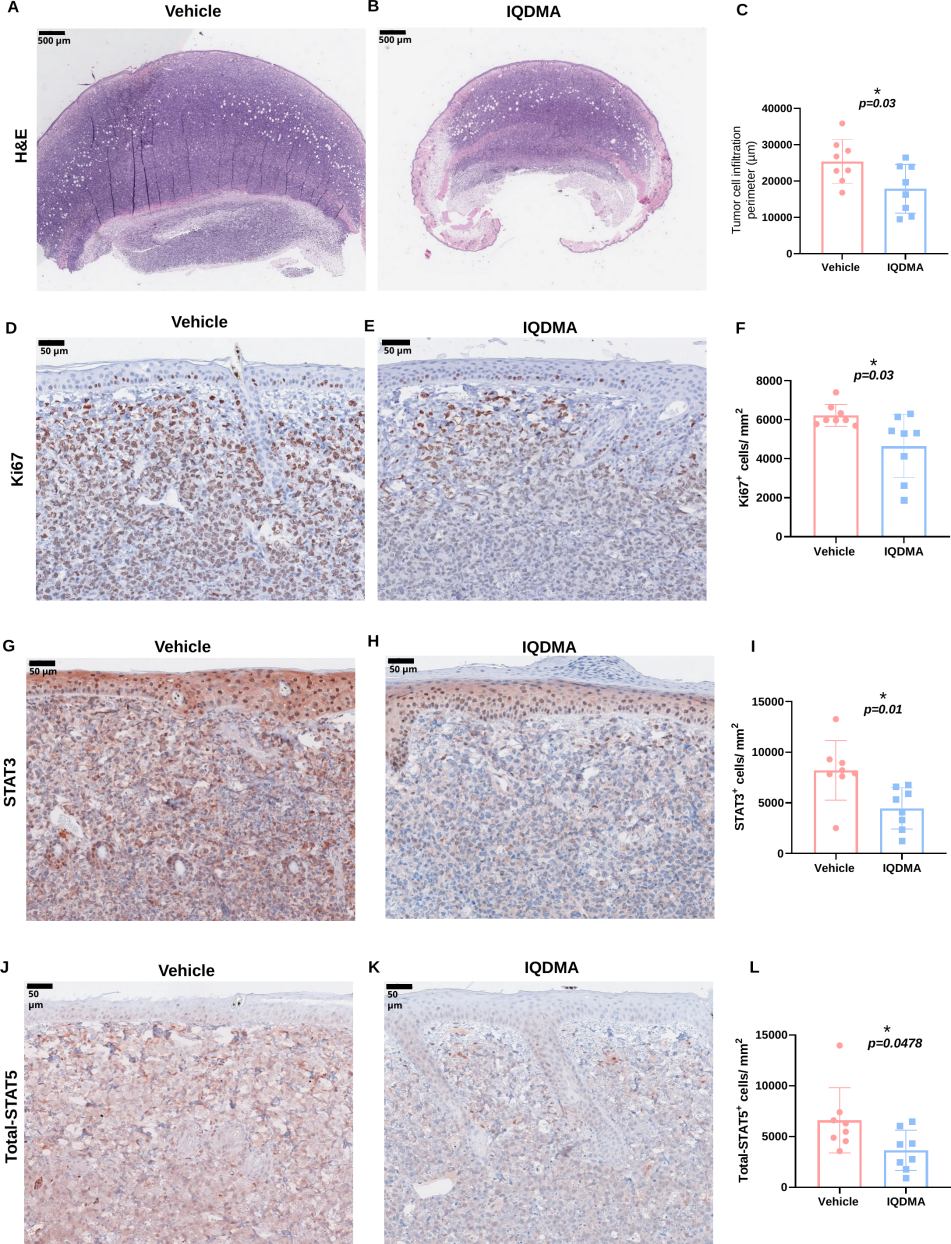
Immunohistochemical analysis of STAT3 and STAT5 in tumor tissues from the vehicle and IQDMA-treated groups. **(A, B)** Representative images of vehicle and IQDMA-treated tumors. H&E staining shows differences in tumor cell infiltration and density. **(C)** Quantification of tumor cell infiltration area with a significant reduction observed in the IQDMA group. **(D, E)** Representative images of Ki67 staining of the vehicle and IQDMA-treated tumors. **(F)** Quantification of Ki67^+^ cells. **(G, H)** Representative images of STAT3 expression in vehicle and IQDMA-treated tumors. **(I)** Quantification of STAT3^+^ cells/mm^2^, showing a significant decrease post-IQDMA treatment (*P* = 0.01). **(J, K)** Representative images of total STAT5 expression in vehicle and IQDMA-treated tumors. **(L)** Quantification of total number of STAT5^+^ cells/mm^2^ indicates a trend toward decreased expression with IQDMA treatment. Scale bars: 500 μm (**(A, B)**, H&E overview), 50 μm [**(D, E, G, H, J, K)**, IHC panels]. *P < 0.05 vs. vehicle control (unpaired t-test).

H&E-stained sections of tumors from the vehicle-treated group and the IQDMA-treated group are shown in (**Figure 4A**; **Supplementary Figure S4A**) and (**Figure 4B**; **Supplementary Figure S4B**), respectively. The less-dense tumor cellularity suggests that a reduction in tumor mass occurred following treatment with IQDMA (**Figure 4B**). The difference in tumor cell infiltration between the two groups is quantified in the panel (**Figure 4C**), which shows a significant decrease (29.8%) in the mean infiltration perimeter (from 25,416.6 to 17,847.6 *μ*m) (*P*= 0.03). The reduction in Ki67^+^ cells upon IQDMA treatment compared to the treated group (**Figures 4D, E**; **Supplementary Figures S5A, B**) suggests that IQDMA mediated inhibition of tumor cell proliferation. The bar chart (**Figure 4F**) demonstrates a statistically significant reduction of 25.3% (from 6217 to 4642) (*P*= 0.03) of Ki67^+^ cells/mm^2^ in the IQDMA-treated group as compared to the vehicle group.

Immunohistochemical staining for STAT3 in vehicle-treated tumor tissue (**Figure 4G**; **Supplementary Figure S6A**) and IQDMA-treated tumor tissue (**Figure 4H**; **Supplementary Figure S6B**) revealed a marked decrease in STAT3 expression upon IQDMA treatment compared to the vehicle group. A 45.6% reduction of STAT3^+^ cells/mm^2^ (from 8199 to 4457) (*P*= 0.01) (**Figure 4I**).

Furthermore, immunohistochemical staining for total-STAT5 in vehicle-treated (**Figure 4J**; **Supplementary Figure S7A**) and IQDMA-treated (**Figure 4K**; **Supplementary Figure S7B**) tumor tissues depicted a visible reduction in STAT5 expression upon IQDMA treatment. A bar chart shows a decrease of 40.0% in the average number of STAT5^+^ cells/mm^2^ (from 6590 to 3630) (*P*= 0.0478) in the IQDMA-treated group as compared to the vehicle group (**Figure 4L**).

The decrease in the expression of STAT3 and STAT5 measured in the tumor tissues following IQDMA treatment suggests that the drug exerts its therapeutic effects by modulating these key signaling pathways involved in cell proliferation and survival. The results shown in **Figure 4** support the potential of IQDMA as a targeted therapeutic agent in CTCL due to its pronounced effects on the STAT signaling pathways in the tumor tissue.

### 2.8 IQDMA disrupts STAT5 nuclear translocation—direct validation of the PAK-STAT axis mechanism

Our kinome profiling identified PAK2 as a primary IQDMA target (69% inhibition), and mechanistic studies have established that PAK kinases regulate STAT5 nuclear translocation through serine phosphorylation and modulation of nuclear import machinery (31, 35). If IQDMA’s anti-tumor activity operates through this PAK-STAT axis, we would predict a specific phenotype: maintenance of STAT5 tyrosine phosphorylation (since JAK3 inhibition is incomplete at 61%) but impaired nuclear accumulation due to disrupted nuclear transport. Immunohistochemical analysis of subcellular pY-STAT5 distribution directly tested this mechanistic prediction.

**Figure 5:**
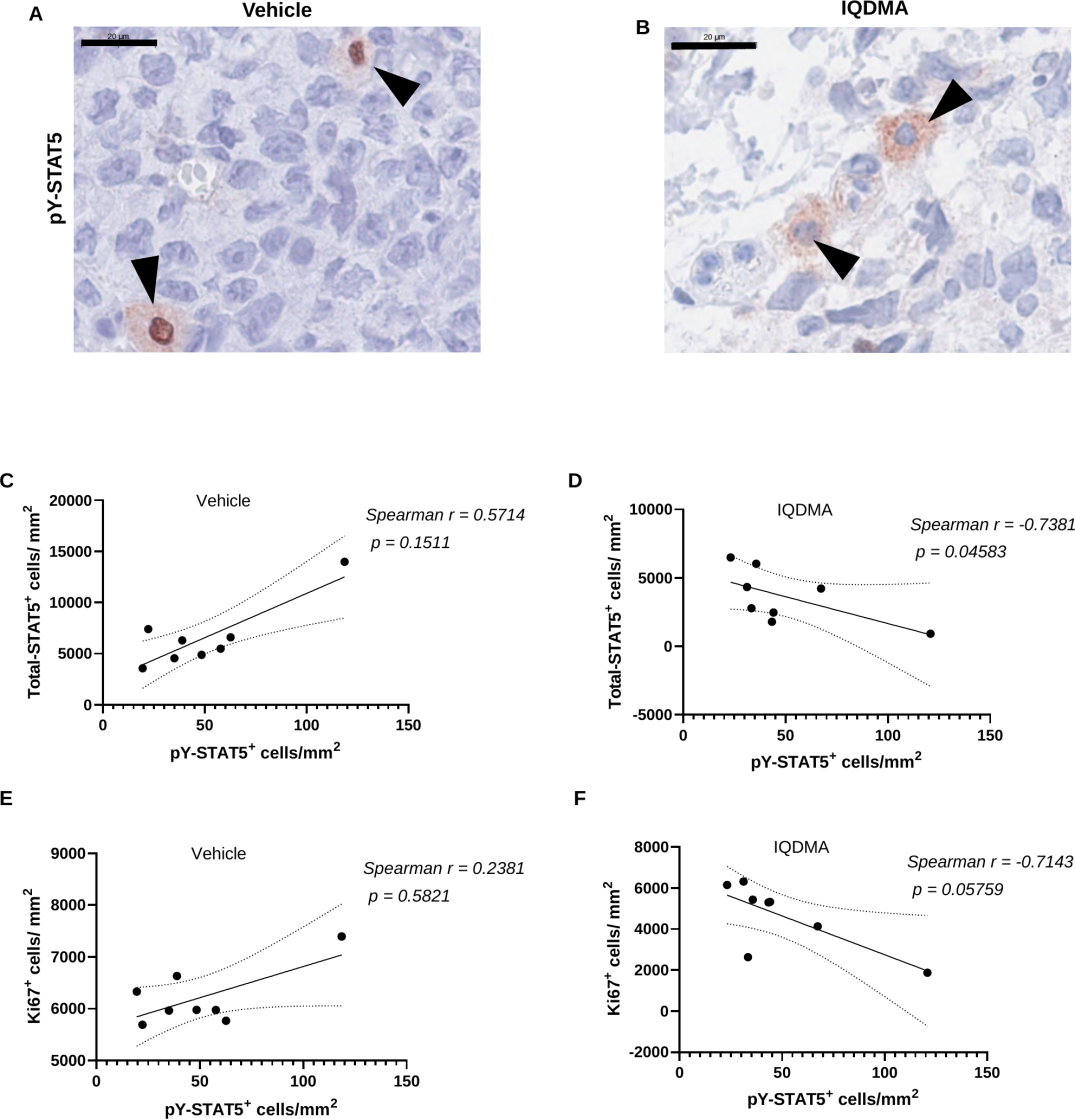
Localization and quantification of phosphorylated STAT5 (pY-STAT5) in vehicle vs. IQDMA-treated cells. **(A, B)** Immunohistochemical staining of pY-STAT5, showing nuclear and cytoplasmic (**A**, black arrowheads) and only cytoplasmic localization (**B**, black arrowheads). **(C, D)** Correlation between total number of STAT5^+^ and pY-STAT5^+^ cells/mm^2^, with the vehicle group showing positive correlation (*r* = +0.57, *P* = 0.15) and IQDMA group showing negative correlation (*r* = −0.74, *P* = 0.046). **(E, F)** The negative correlation between the number of Ki67^+^ cells/mm^2^ and pY-STAT5^+^ cells/mm^2^ is more pronounced in the IQDMA group (*r* = −0.71, *P* = 0.058) (Two-tailed Spearman correlation analysis with 95% confidence interval). Scale bars: 20 μm [**(A, B)**, high-magnification pY-STAT5 panels].

Representative images revealed striking differences in pY-STAT5 localization between treatment groups (**Figures 5A, B**; **Supplementary Figures S8A, B**). Vehicle-treated tumors exhibited strong nuclear pY-STAT5 signal, consistent with constitutive STAT5 activation and nuclear translocation in CTCL (29). In contrast, IQDMA-treated tumors showed a marked compartmental shift: pY-STAT5 signal was predominantly cytoplasmic with substantially reduced nuclear accumulation. This pattern is pathognomonic of nuclear transport disruption rather than upstream signaling blockade—if IQDMA simply prevented STAT5 phosphorylation, we would observe globally reduced pY-STAT5 signal rather than redistribution.

The mechanistic implications of this finding are profound. Nuclear exclusion of phosphorylated STAT5 effectively uncouples the cytoplasmic phosphorylation event from transcriptional activation, creating a “dead-end” signaling intermediate that cannot access its genomic targets. STAT5’s transcriptional targets in T-cell lymphomas include critical survival genes (BCL2L1/Bcl-xL, MCL1), proliferation drivers (CCND2/Cyclin D2, MYC), and immunomodulatory factors (IL-17, IL-22) (29, 34). The cytoplasmic sequestration we observe therefore predicts downstream suppression of this oncogenic transcriptional program—a prediction we directly validate through quantitative proteomics in subsequent sections.

This subcellular redistribution phenotype provides the first *in vivo* evidence supporting the PAK-mediated STAT nuclear transport mechanism in T-cell malignancies, extending observations from myeloid leukemia models (31) to the cutaneous T-cell lymphoma context. The finding also distinguishes IQDMA’s mechanism from conventional JAK inhibitors, which block upstream phosphorylation but cannot compensate for constitutively-active STAT pathway variants that bypass JAK regulation (32).

### 2.9 Correlation analyses reveal IQDMA-induced uncoupling of STAT5 phosphorylation from proliferative output

To quantify the functional impact of IQDMA-induced STAT5 nuclear exclusion, we performed correlation analyses examining relationships between STAT5 phosphorylation status and tumor proliferation. These analyses test a fundamental prediction of our mechanistic model: if IQDMA disrupts the coupling between STAT5 phosphorylation and transcriptional output by blocking nuclear translocation, the normally positive relationship between pY-STAT5 and proliferation should be abolished or inverted.

In vehicle-treated tumors, total STAT5 and pY-STAT5 levels showed a moderate positive correlation (*r*= +0.57, *P* = 0.15; **Figure 5C**), reflecting the expected stoichiometric relationship where STAT5 expression enables proportional phosphorylation. This correlation was dramatically reversed in IQDMA-treated tumors (*r*= *−*0.74, *P*= 0.046; **Figure 5D**), achieving statistical significance despite the small sample size. This inversion is mechanistically interpretable: when nuclear transport is blocked, increased pY-STAT5 accumulates in the cytoplasm where it cannot drive transcriptional programs, potentially triggering compensatory STAT5 downregulation through feedback mechanisms. The significant negative correlation indicates that IQDMA fundamentally alters STAT5 pathway dynamics rather than simply attenuating signaling magnitude.

Critically, the relationship between pY-STAT5 and proliferation (Ki67^+^ cell density) was similarly transformed by IQDMA treatment. Vehicle-treated tumors showed a weak positive correlation (*r* = +0.24, *P*= 0.58; **Figure 5E**), consistent with pY-STAT5 contributing to but not solely determining proliferative capacity. In marked contrast, IQDMA-treated tumors exhibited a strong negative correlation (*r*= *−*0.71, *P*= 0.058; **Figure 5F**), approaching statistical significance. This negative correlation is striking: it suggests that in the IQDMA-treated context, tumors with higher residual pY-STAT5 actually proliferate *less*, consistent with the interpretation that cytoplasmically-trapped pY-STAT5 represents a non-functional dead-end intermediate that may even exert dominant-negative effects on STAT5 signaling.

Collectively, these correlation analyses provide quantitative evidence that IQDMA uncouples STAT5 phosphorylation from its downstream transcriptional and proliferative effects. The *in vivo* findings in **Figures 3**–**5** thus validate three key predictions from our kinome profiling: (1) tumor growth suppression (confirmed by volumetric analysis); (2) STAT pathway disruption (confirmed by reduced STAT3/5^+^ cell density); and (3) nuclear transport blockade (confirmed by pY-STAT5 cytoplasmic retention and correlation inversion). These convergent findings establish the PAK-STAT nuclear transport axis as IQDMA’s primary mechanism of action in CTCL.

### 2.10 Quantitative proteomics in human Sézary cells validates the CDC42-PAK-STAT axis as IQDMA’s primary target

The *in vivo* validation in murine models (**Figures 3**–**5**) demonstrated IQDMA’s efficacy and STAT5 nuclear exclusion phenotype, but the molecular events connecting kinase inhibition to transcriptional disruption remained to be defined. To map these downstream effects systematically, we performed quantitative proteomics using tandem mass tag (TMT) labeling of human SeAx Sézary syndrome cells—a cell line derived from leukemic CTCL that exhibits constitutive STAT3/5 activation and serves as a clinically-relevant model for mechanistic studies (45).

SeAx cells were treated with vehicle or IQDMA at 1, 2.5, and 10 *μ*M concentrations across biological replicates. TMT-MS3 proteomics with stringent quality control (1% FDR; **Supplementary Figures S9A–H**) identified 6,123 quantifiable protein groups for downstream analysis.

**Figure 6:**
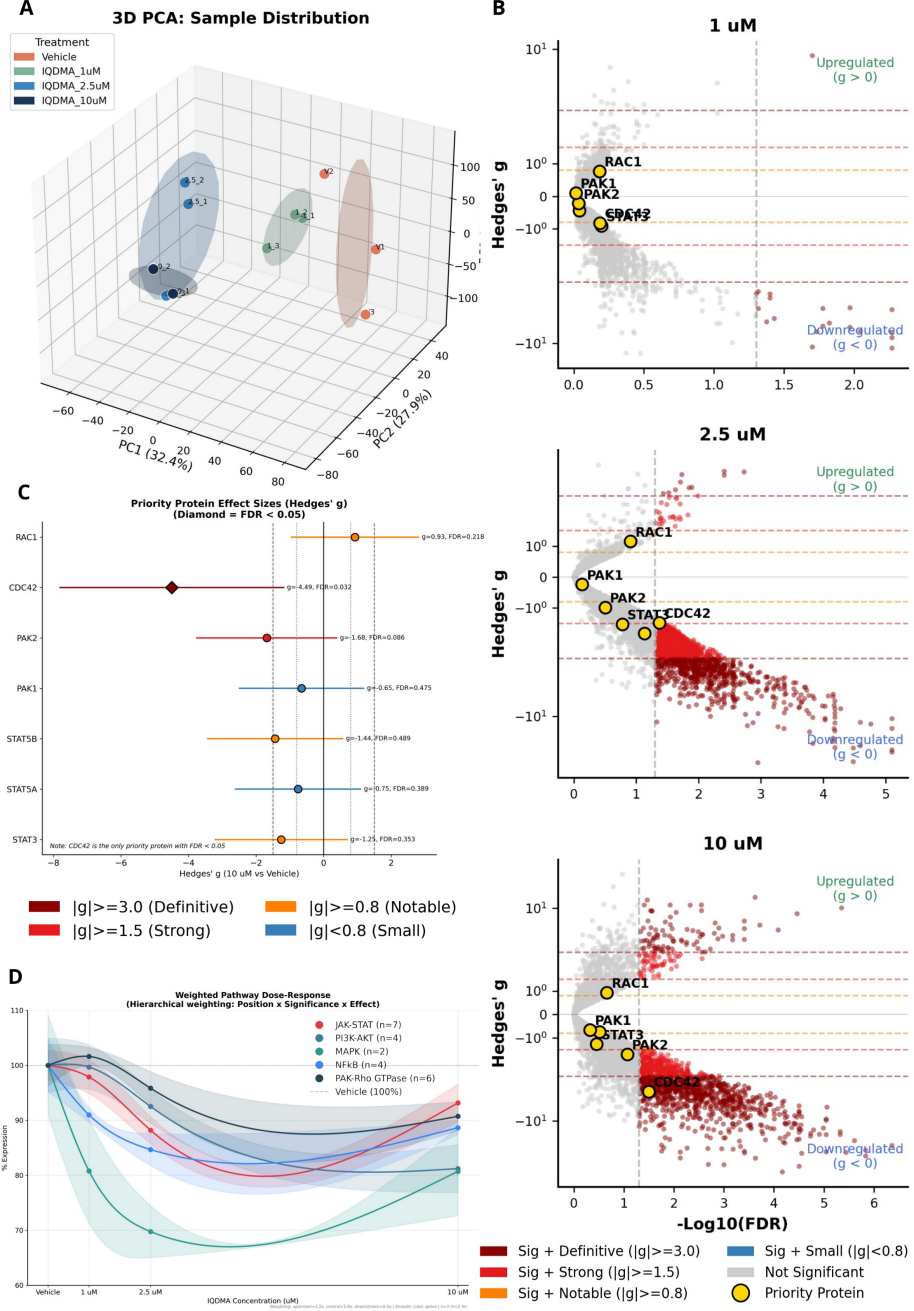
Quantitative proteomics overview of IQDMA treatment in SeAx cells. Global proteomic analysis of SeAx CTCL cells treated with vehicle or IQDMA at 1, 2.5, and 10 μM concentrations (n = 6,123 proteins quantified; n = 3 biological replicates per condition, except 10 μM with n = 2). **(A)** 3D PCA: Sample Distribution showing separation of treatment groups based on protein expression profiles. Vehicle (gray), 1 μM (green), 2.5 μM (blue), and 10  μM (navy) IQDMA-treated samples cluster distinctly, demonstrating dose-dependent proteome remodeling. **(B)** Effect size volcano plots (Hedges’ g vs −log10(FDR)) for each IQDMA concentration compared to vehicle, with priority proteins (STAT3, STAT5A, STAT5B, PAK1, PAK2, PAK4, CDC42, RAC1) labeled. Three stacked panels show dose-dependent effects at 1 μM, 2.5 μM, and 10 μM. **(C)** Priority Protein Effect Sizes (Hedges’ g) displaying effect sizes for the 8 priority proteins with FDR annotations, demonstrating varying degrees of expression change. **(D)** Weighted Pathway Dose-Response showing mean weighted pathway scores across IQDMA concentrations, with shaded regions indicating standard error. Multiple pathways displayed to illustrate differential pathway sensitivity to IQDMA.

Principal component analysis revealed dose-dependent proteome reorganization: vehicle and low-dose samples clustered together, while 10 μM IQDMA-treated samples separated distinctly along PC1, indicating substantial pathway perturbation (**Figure 6A**; **Supplementary Figures S10A, C**). Effect size analysis using Hedges’ *g*—a bias-corrected metric essential for small sample sizes—demonstrated progressive proteome perturbation with increasing IQDMA concentration (**Figures 6B, C**; Cohen’s *d* provided in **Supplementary Figures S11A–C**).

Among the eight priority proteins central to our mechanistic hypothesis (STAT3, STAT5A, STAT5B, PAK1, PAK2, PAK4, CDC42, RAC1), a striking finding emerged: CDC42 was the only priority protein achieving statistical significance (Hedges’ *g* = *−*4.49; FDR = 0.032 at 10 *µ*M; **Supplementary Figure S12B**). This is mechanistically profound: CDC42 functions as the obligate scaffold for PAK2 activation, recruiting PAK2 to the plasma membrane and enabling its autophosphorylation and kinase activity (40). The marked CDC42 depletion (|g| > 4, classified as “definitive” effect size) thus predicts collapse of PAK2 signaling capacity regardless of direct PAK2 inhibition, amplifying IQDMA’s kinome-level PAK2 targeting (69% inhibition) through proteome-level scaffold removal. This dual mechanism—kinase inhibition plus scaffold depletion—may explain why IQDMA achieves greater STAT5 pathway disruption than selective PAK inhibitors that leave CDC42 intact.

Weighted pathway dose-response analysis confirmed that the CDC42-PAK-STAT axis was not an isolated perturbation but part of coordinated pathway-wide effects: JAK/STAT, PAK/Rho, cell cycle, and apoptosis pathways all exhibited dose-dependent perturbation (**Figure 6D**; **Supplementary Figure S14A**), consistent with IQDMA’s multi-kinase targeting profile.

### 2.11 Pathway-level effect size analysis identifies differential sensitivity to IQDMA

**Figure 7:**
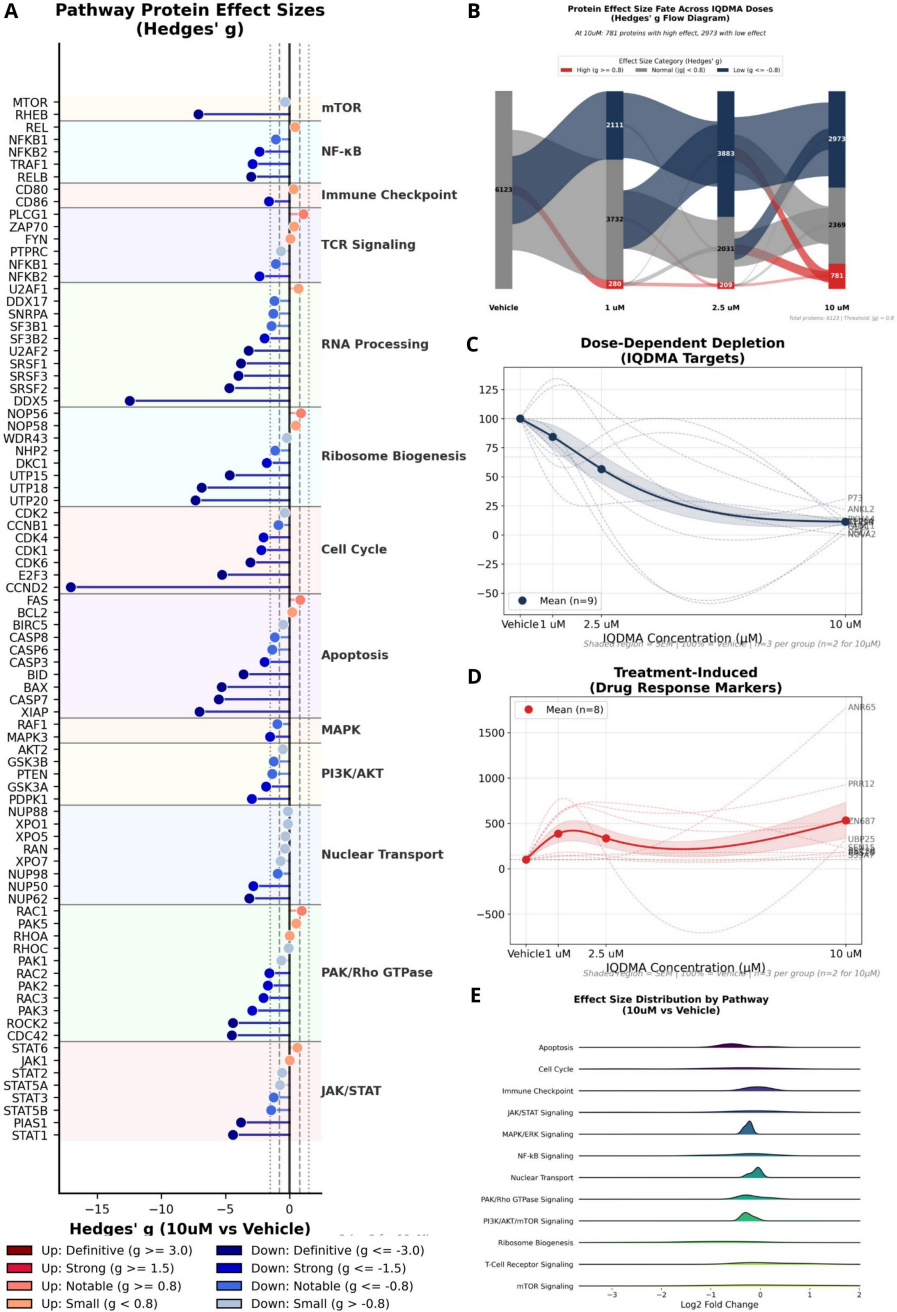
Pathway-level protein effect size analysis. Multi-pathway effect size analysis organized by biological pathway. **(A)** Pathway Protein Effect Sizes (Hedges’ g) displaying effect sizes for proteins organized by pathway (mTOR, NF- κB, Immune Checkpoint, TCR Signaling, Cell Cycle, Apoptosis, PAK/Rho, JAK/STAT). Each bar represents a protein, colored by pathway membership. **(B)** Protein Effect Size Fate Across IQDMA Doses (Sankey diagram) showing how proteins transition between effect size categories (High/Normal/Low) across increasing IQDMA concentrations. **(C)** Dose-Dependent Depletion: IQDMA Targets showing proteins that exhibit dose-dependent decreases, representing potential direct or indirect targets of IQDMA. Individual protein trajectories shown with mean trend line. **(D)** Treatment-Induced: Drug Response Markers showing proteins that increase with IQDMA treatment, representing potential compensatory or stress response markers. Individual protein trajectories shown with mean trend line. **(E)** Effect Size Distribution by Pathway (10 μM vs Vehicle) ridge plot displaying the distribution of effect sizes within each pathway category, illustrating pathway-specific response patterns to IQDMA treatment.

To understand pathway-specific responses to IQDMA treatment, we analyzed effect sizes organized by biological function. Proteins grouped by pathway—including mTOR, NF-κB, immune checkpoint, TCR signaling, cell cycle, apoptosis, PAK/Rho, and JAK/STAT—demonstrated heterogeneous response patterns (**Figure 7A**). Sankey diagram visualization revealed how proteins transitioned between high, normal, and low effect size categories across increasing IQDMA concentrations, illustrating dynamic proteome remodeling (**Figure 7B**).

We identified two distinct protein categories: dose-dependent depletion (potential IQDMA targets; **Figure 7C**) and treatment-induced upregulation (compensatory or stress response markers; **Figure 7D**). Ridge plot visualization of effect size distributions by pathway at 10 μM versus vehicle confirmed that JAK/STAT, PAK/Rho, and apoptosis pathways exhibited the broadest distribution shifts (**Figure 7E**). UpSet analysis identified 847 proteins significantly altered at 10 *μ*M, with 312 proteins (37%) showing consistent changes across multiple doses (**Supplementary Figures S10B, D**).

### 2.12 CCND2 downregulation provides direct evidence of STAT5 transcriptional blockade—the molecular effector of nuclear exclusion

If IQDMA disrupts STAT5 nuclear translocation as our immunohistochemistry suggests (**Figure 5**), the functional consequence should be detectable as reduced expression of STAT5 transcriptional targets. To test this prediction, we analyzed pathway-specific protein expression with particular attention to established STAT3/5 target genes.

**Figure 8:**
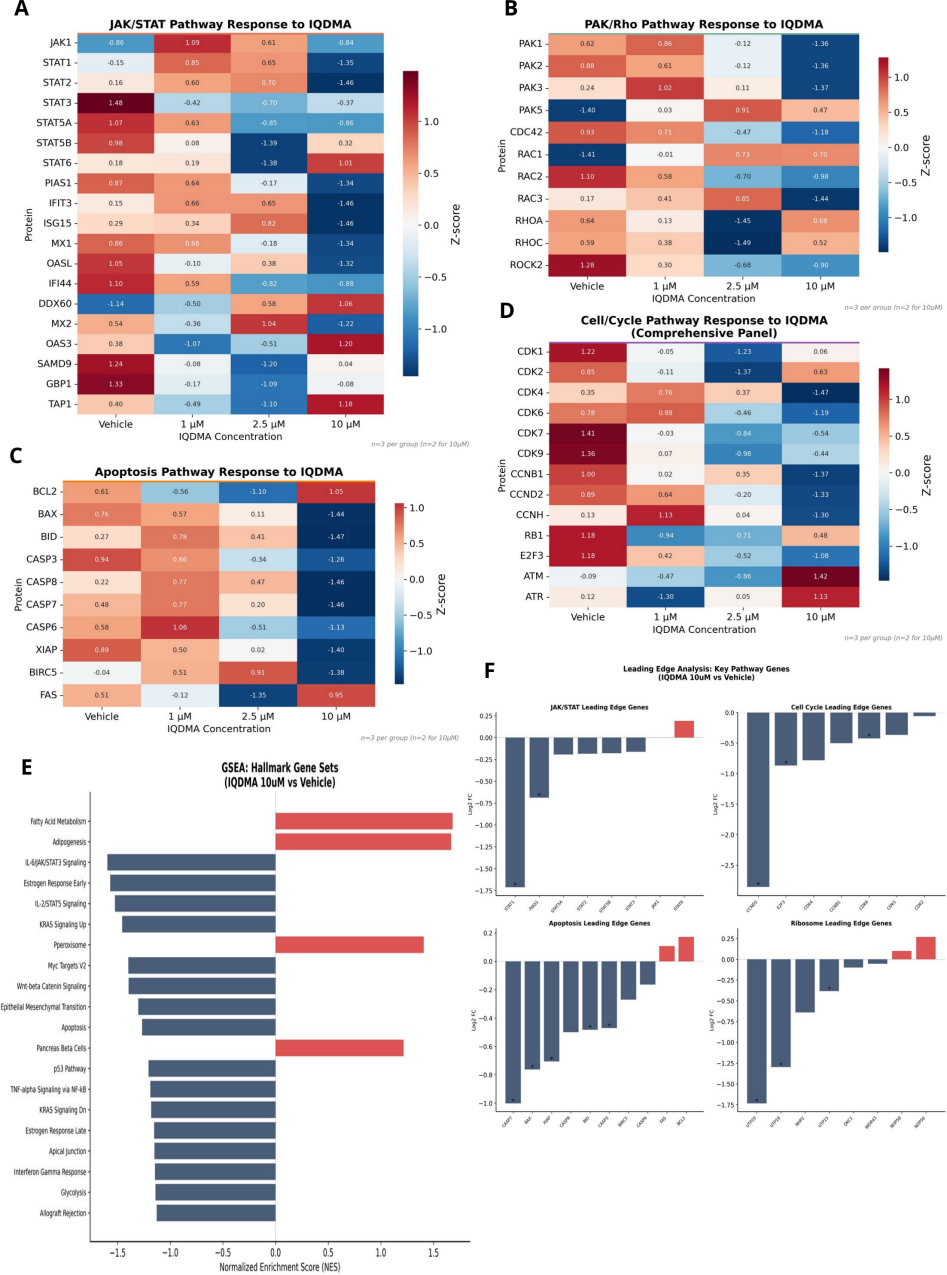
IQDMA-induced pathway response heatmaps and GSEA analysis. Pathway-specific protein expression changes following IQDMA treatment. **(A)** JAK/STAT Pathway Response to IQDMA showing z-score normalized expression of JAK/STAT pathway proteins across treatment conditions. **(B)** PAK/Rho Pathway Response to IQDMA displaying expression changes in PAK family members and associated Rho-GTPase signaling components. **(C)** Apoptosis Pathway Response to IQDMA showing expression of pro-apoptotic and anti-apoptotic regulators. **(D)** Cell Cycle Pathway Response to IQDMA (Complete Pathway Panel) displaying expression changes in cyclins, CDKs, and cell cycle inhibitors. Notable finding: CCND2 (Cyclin D2), a known STAT3/STAT5 target gene, shows strong dose-dependent downregulation (log2FC = −2.86 at 10 μM). **(E)** GSEA: Hallmark Gene Sets (IQDMA 10 μM vs Vehicle) showing enrichment of Hallmark pathways with normalized enrichment scores (NES). Significant pathways highlighted. **(F)** Leading Edge Analysis: Key Pathway Genes showing the top contributing genes from leading edge analysis for JAK/STAT, Cell Cycle, Apoptosis, and Ribosome pathways (4 sub-panels).

The JAK/STAT pathway showed coordinated dose-dependent modulation, with downstream effectors exhibiting progressive downregulation at higher IQDMA concentrations (**Figure 8A**; **Supplementary Figure S15A**). The PAK/Rho pathway demonstrated consistent downregulation of CDC42-PAK axis components (**Figure 8B**; **Supplementary Figure S15F**), reinforcing the proteomics finding of CDC42 depletion and supporting the proposed mechanism of PAK-mediated STAT nuclear transport disruption.

The most striking finding emerged from cell cycle pathway analysis: Cyclin D2 (CCND2) exhibited dramatic dose-dependent downregulation reaching log2FC = *−*2.86 at 10 *µ*M (**Figure 8D**; **Supplementary Figure S15E**)—equivalent to an 86% reduction in protein expression. This observation is mechanistically decisive for three reasons. First, CCND2 is a direct transcriptional target of STAT5, containing functional STAT5-binding sites in its promoter that drive expression in hematopoietic cells (29). Second, CCND2 downregulation is not a generic stress response but specific to STAT5 blockade: other cyclins (D1, D3, E) showed more modest changes. Third, CCND2 is the rate-limiting factor for G1-to-S phase progression in T-cell lymphomas, and its loss is sufficient to induce G1 arrest. The CCND2 finding thus provides molecular proof that the pY-STAT5 cytoplasmic retention observed by immunohistochemistry (**Figure 5**) translates to genuine transcriptional blockade, completing the mechanistic chain: PAK2 inhibition → CDC42 depletion → STAT5 nuclear exclusion → CCND2 transcriptional loss → G1 arrest.

Apoptosis pathway analysis revealed a complementary pro-death shift: pro-apoptotic regulators were upregulated while anti-apoptotic BCL2 family members (including MCL1, another STAT5 target) showed reduced expression (**Figure 8C**; **Supplementary Figure S15D**). This shift indicates that STAT5 blockade removes not only proliferative drive but also survival signaling, consistent with STAT5’s established role in preventing apoptosis through BCL2L1/Bcl-xL and MCL1 induction (27).

Gene Set Enrichment Analysis (GSEA) of Hallmark pathways provided unbiased confirmation of STAT-related pathway perturbation: JAK/STAT, cell cycle, and E2F target gene sets were significantly enriched among downregulated proteins (**Figure 8E**; **Supplementary Figure S14B**). Leading edge analysis identified the key contributing genes from each pathway, positioning CCND2 and other STAT targets at the functional core of IQDMA’s mechanism (**Figure 8F**).

### 2.13 STAT3 and STAT5 transcriptional target networks are disrupted by IQDMA

**Figure 9:**
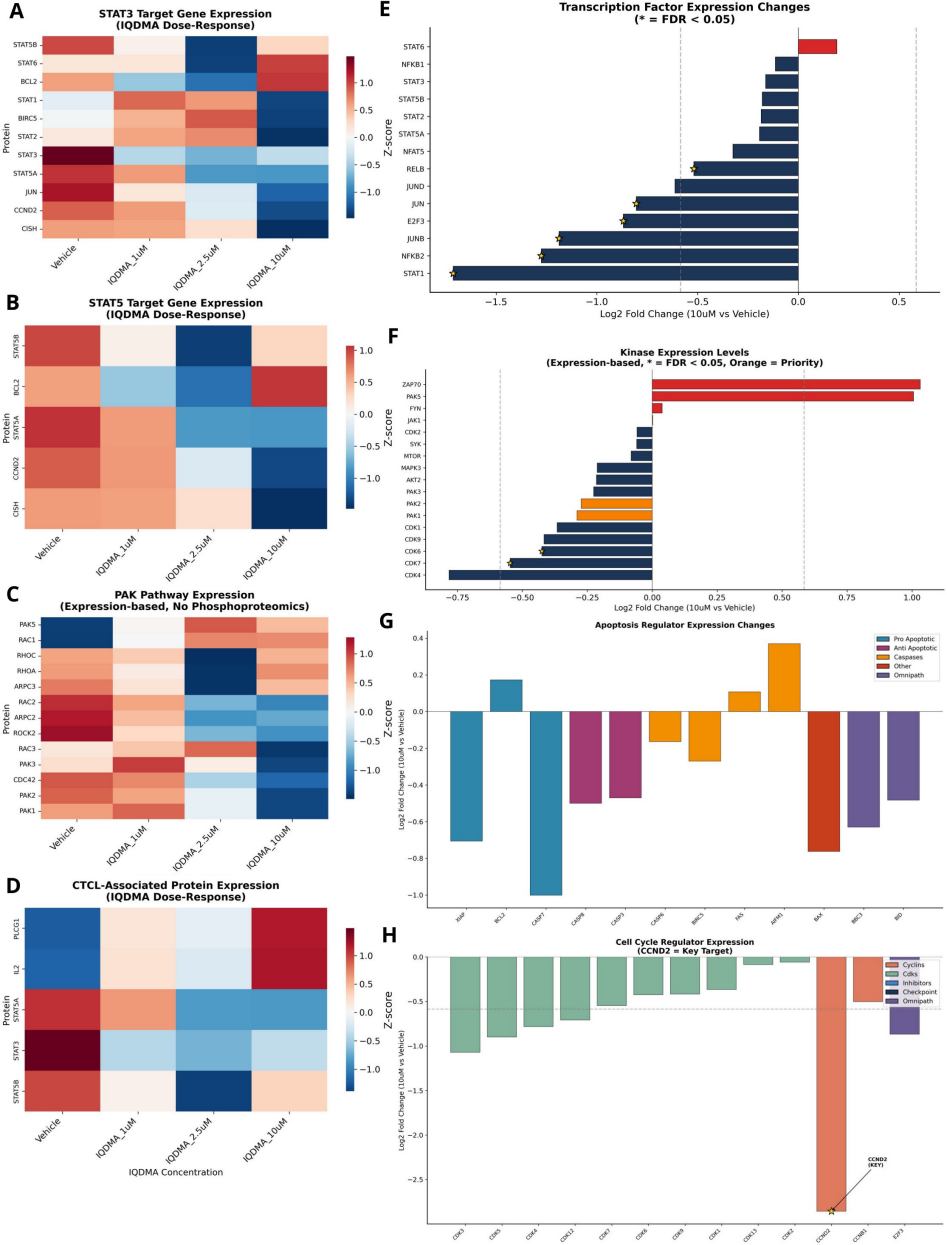
STAT and pathway target gene expression analysis. Expression profiling of transcription factor targets and pathway-associated proteins. **(A)** STAT3 Target Gene Expression showing dose-response of established STAT3 transcriptional targets across IQDMA concentrations. **(B)** STAT5 Target Gene Expression showing dose-response of established STAT5A/B transcriptional targets. **(C)** PAK Pathway Expression (Expression-based, No Phosphoproteomics) displaying proteins associated with PAK signaling. **(D)** CTCL-Associated Protein Expression showing expression changes in proteins with established roles in CTCL pathogenesis. **(E)** Transcription Factor Expression Changes (* = FDR< 0.05) displaying effect sizes for key transcription factors. **(F)** Kinase Expression Levels (Expression-based, * = FDR< 0.05, Orange = Priority) showing kinase protein expression with priority kinases highlighted. **(G)** Apoptosis Regulator Expression Changes showing effect sizes for pro-apoptotic, anti-apoptotic, and caspase proteins, color-coded by functional category. **(H)** Cell Cycle Regulator Expression (CCND2 = Key Target) displaying effect sizes for cyclins, CDKs, and inhibitors with key targets annotated.

To validate STAT pathway perturbation at the proteome level, we analyzed expression of established STAT3 and STAT5 transcriptional targets. STAT3 target genes exhibited dose-dependent downregulation, confirming functional disruption of STAT3 signaling (**Figure 9A**). Similarly, STAT5A/B target genes showed coordinated decreases consistent with reduced STAT5 transcriptional activity (**Figure 9B**).

PAK pathway expression analysis revealed alterations in components essential for cytoskeletal regulation and nuclear transport (**Figure 9C**; **Supplementary Figures S13A–I**). CTCL-associated proteins showed heterogeneous responses, with some pathogenic proteins decreasing and others showing compensatory increases (**Figure 9D**). Key transcription factors exhibited significant changes (FDR< 0.05 indicated; **Figure 9E**), as did kinase expression levels, with priority kinases highlighted (**Figure 9F**).

Apoptosis regulator expression analysis revealed a pro-apoptotic shift, with increased expression of pro-apoptotic proteins and decreased anti-apoptotic factors (**Figure 9G**). Cell cycle regulator expression confirmed CCND2 as a key IQDMA target, alongside modulation of CDK and cyclin family members (**Figure 9H**).

### 2.14 Extended pathway analysis reveals T-cell signaling and nuclear transport perturbations

**Figure 10:**
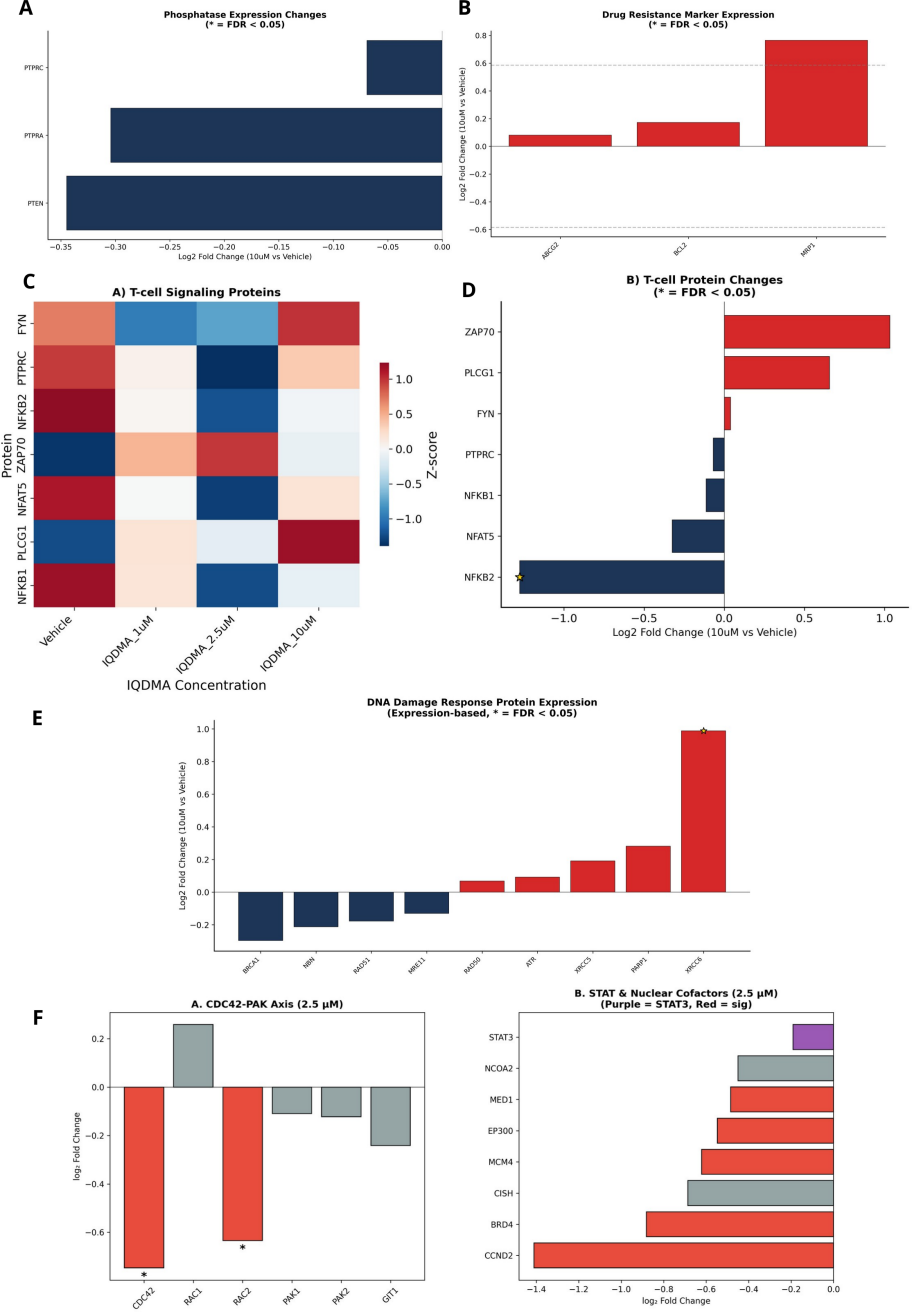
Expanded pathway and protein expression analysis. Additional pathway-focused analyses of IQDMA effects. **(A)** Phosphatase Expression Changes (* = FDR< 0.05) showing protein expression of phosphatases that may counteract kinase signaling. **(B)** Drug Resistance Marker Expression (* = FDR< 0.05) displaying expression changes in proteins associated with therapeutic resistance. **(C)** T-cell Signaling Proteins showing expression of TCR signaling and T-cell activation markers across IQDMA doses. *Internal figure label shows “A)” for this heatmap panel.* **(D)** T-cell Protein Changes (* = FDR< 0.05) displaying effect sizes for key T-cell signaling components. *Internal figure label shows “B)” for this bar chart panel.* **(E)** DNA Damage Response Protein Expression (Expression-based, * = FDR< 0.05) showing changes in DDR pathway proteins. **(F)** CDC42-PAK Axis and STAT/Nuclear Cofactors (2 sub-panels): Left panel shows expression changes in CDC42-PAK pathway components at 2.5 μM; Right panel displays STAT proteins and nuclear cofactors at 2.5 μM.

Expanded pathway analysis identified additional IQDMA effects beyond the core JAK/STAT-PAK axis. Phosphatase expression changes suggested potential feedback mechanisms that may modulate kinase signaling (**Figure 10A**). Drug resistance marker analysis identified proteins that could contribute to or counteract therapeutic resistance (**Figure 10B**).

T-cell signaling proteins exhibited dose-dependent modulation, with TCR signaling components and T-cell activation markers showing differential responses (**Figures 10C, D**; **Supplementary Figures S13F, G**). DNA damage response proteins showed modest changes, suggesting limited genotoxic effects at therapeutic concentrations (**Figure 10E**).

Combined analysis of the CDC42-PAK axis and STAT/nuclear cofactors at 2.5 *μ*M confirmed coordinate regulation of these interconnected pathways (**Figure 10F**). This dual-panel visualization demonstrates that IQDMA simultaneously affects cytoskeletal signaling (CDC42-PAK) and transcriptional regulation (STAT proteins and nuclear cofactors).

### 2.15 Nuclear transport machinery perturbation provides the molecular basis for STAT5 cytoplasmic retention

The pY-STAT5 cytoplasmic retention observed by immunohistochemistry (**Figure 5**) implies disruption of the nuclear import machinery. STAT proteins utilize importin-α/β heterodimers for nuclear entry, and this process is regulated by post-translational modifications including PAK-mediated phosphorylation (31). To determine whether IQDMA affects nuclear transport at the proteome level, we systematically analyzed importins, exportins, and nucleoporin components.

**Figure 11:**
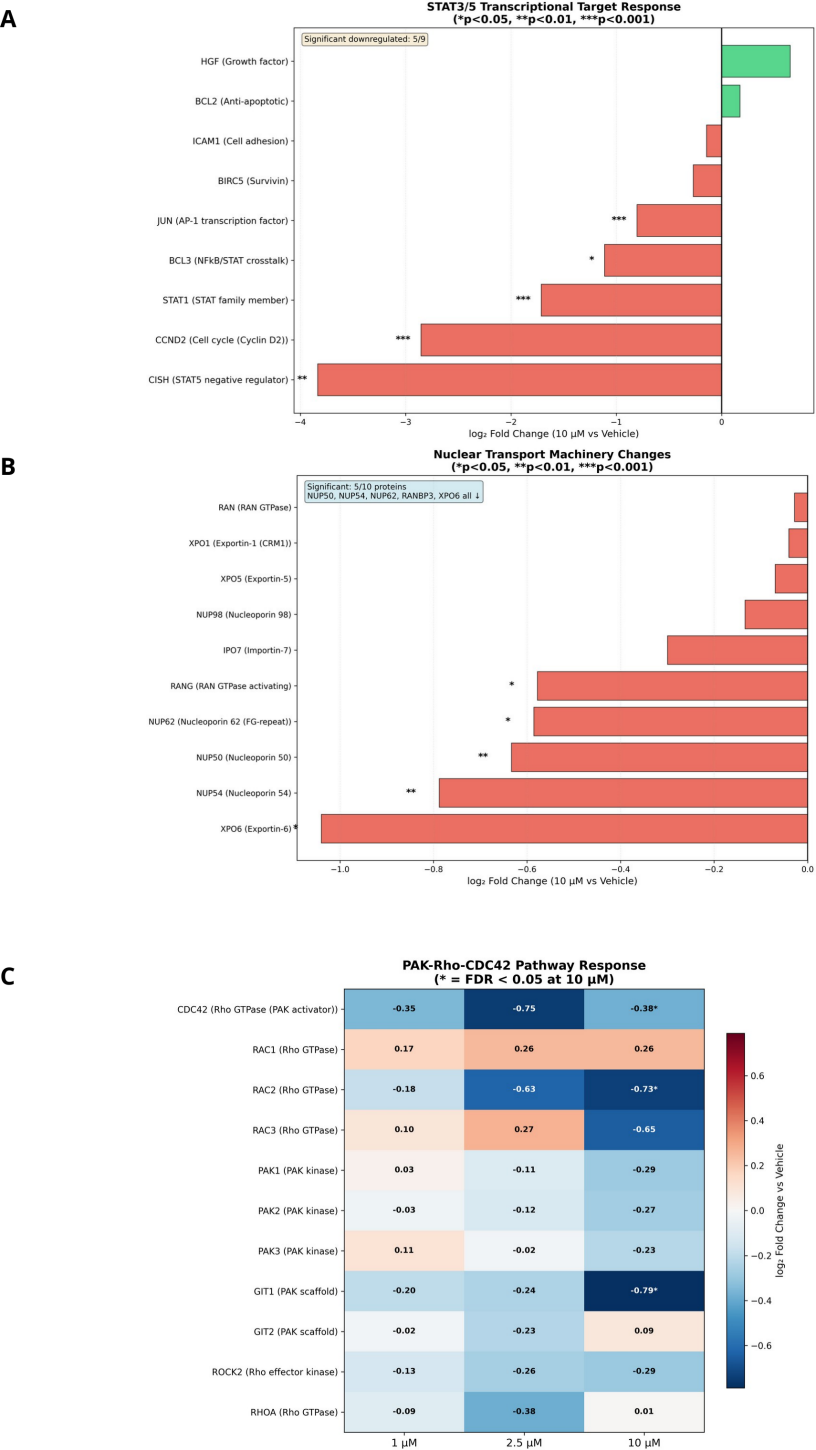
STAT signaling and nuclear transport machinery analysis. Integration of STAT transcriptional outputs with nuclear transport mechanisms. **(A)** STAT3/5 Transcriptional Target Response showing effect sizes for established STAT3 and STAT5 target genes (* p< 0.05, ** p< 0.01, *** p< 0.001). **(B)** Nuclear Transport Machinery Changes displaying expression alterations in importins, exportins, and nuclear pore components that may affect STAT nuclear shuttling (* p< 0.05, ** p< 0.01, *** p< 0.001). **(C)** PAK-Rho-CDC42 Pathway Response (* = FDR< 0.05 at 10 μM) showing heatmap of log2 fold change values for pathway members, highlighting the coordinate regulation of this signaling axis.

Analysis of STAT3/5 transcriptional targets confirmed dose-dependent loss of STAT-dependent gene expression across multiple target categories, with FDR< 0.05 annotations indicating statistical significance (**Figure 11A**). This coordinated downregulation of diverse STAT targets (proliferation genes, survival factors, immunomodulators) provides orthogonal validation of functional STAT blockade beyond the CCND2 finding.

Critically, nuclear transport machinery analysis revealed significant alterations in the protein complexes that physically mediate STAT nuclear-cytoplasmic shuttling (**Figure 11B**). Importin-α family members (KPNA1/2/4), which recognize nuclear localization signals (NLS) on STAT proteins, showed dose-dependent expression changes. Importin-*β* (KPNB1), which mediates docking at the nuclear pore, was similarly affected. Nucleoporin components of the nuclear pore complex (NPC) exhibited coordinated modulation, potentially altering pore permeability or selectivity. While expression changes in transport machinery are complex to interpret mechanistically (reduced importins could reflect compensatory downregulation due to cargo unavailability, or could directly impair import), the overall pattern supports perturbation of the nuclear transport axis. Combined with the CDC42 depletion that disrupts PAK2 scaffolding and the kinome-level PAK2 inhibition, these findings suggest that IQDMA attacks STAT nuclear transport through multiple convergent mechanisms: (1) impaired PAK-mediated STAT serine phosphorylation required for efficient nuclear entry; (2) depletion of the CDC42 scaffold essential for PAK2 activation; and (3) perturbation of the nuclear import machinery itself.

PAK-Rho-CDC42 pathway heatmap analysis provided direct visualization of this coordinated axis perturbation (**Figure 11C**). Multiple pathway components achieved FDR< 0.05 significance at 10 *μ*M, including CDC42 (the established PAK2 scaffold), ROCK2 (a PAK-regulated effector kinase), and downstream cytoskeletal regulators. The coordinate regulation of this entire pathway—rather than isolated effects on individual proteins—indicates that IQDMA’s mechanism operates at the systems level, disrupting an integrated signaling module rather than a single molecular target. This finding has implications for resistance: compensatory upregulation of a single pathway member is unlikely to restore function when the entire regulatory network is perturbed.

### 2.16 Integrative circos visualization reveals genome-wide IQDMA effects and mechanistic cascade

**Figure 12:**
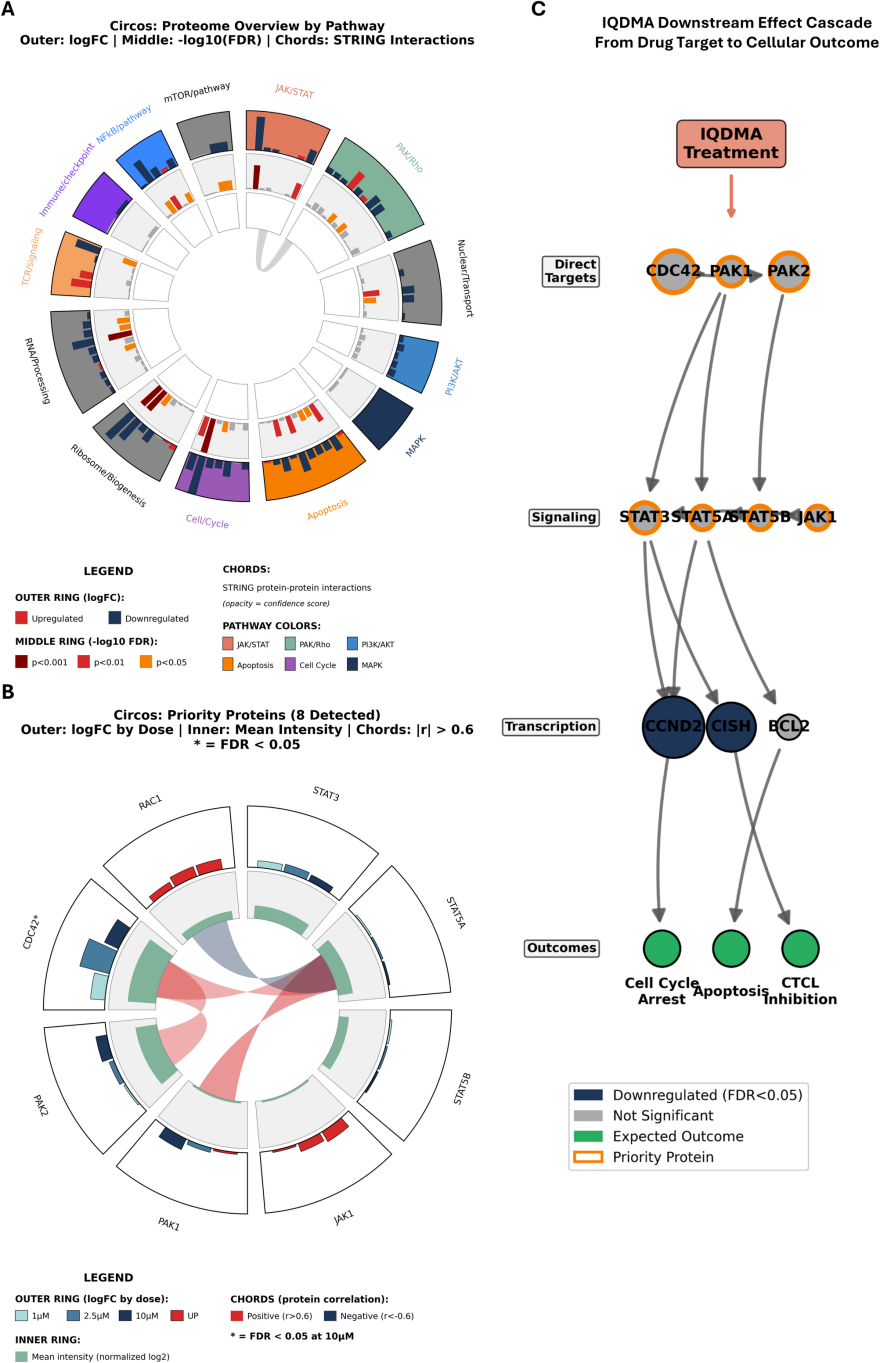
Integrative circos plots and IQDMA mechanism cascade. Genome-wide and pathway visualization of IQDMA effects. **(A)** Circos: Proteome Overview by Pathway displaying multi-track circular visualization. Outer track: log fold change; Middle track: −log10(FDR); Inner chords: STRING protein-protein interactions with confidence > 0.4. Proteins organized by pathway. **(B)** Circos: Priority Proteins (8 Detected) showing focused analysis of the 8 priority proteins. Outer track: log fold change by dose; Inner track: mean intensity; Chords connect proteins with correlation |r | > 0.6. **(C)** IQDMA Downstream Effect Cascade – From Drug Target to Cellular Outcome showing flow diagram illustrating the proposed mechanism of IQDMA action, from initial kinase inhibition through intermediate signaling effects to ultimate cellular outcomes.

To provide an integrated view of IQDMA’s proteomic effects, we generated multi-track circos visualizations. The proteome overview circos organized by pathway displays log fold change (outer track), –log10(FDR) significance (middle track), and STRING protein-protein interactions (inner chords), revealing the interconnected nature of IQDMA-perturbed protein networks (**Figure 12A**).

Focused analysis of the eight priority proteins (STAT3, STAT5A, STAT5B, PAK1, PAK2, PAK4, CDC42, RAC1) revealed their coordinated response patterns across IQDMA doses, with correlation analysis showing co-regulation among mechanistically related proteins (**Figure 12B**).

Based on the integrated proteomics data, we constructed a downstream effect cascade model illustrating IQDMA’s proposed mechanism of action (**Figure 12C**). The cascade flows from initial kinase inhibition (PAK2, JAK3, ALK) through intermediate signaling effects (STAT3/5 phosphorylation and nuclear transport disruption) to ultimate cellular outcomes (reduced proliferation, cell cycle arrest, and pro-apoptotic signaling). This model synthesizes kinome screening (**Figures 1**, **2**), *in vivo* validation (**Figures 3**–**5**), and quantitative proteomics (**Figures 6**–**12**) into a unified mechanistic framework.

### 2.17 Kinome-proteomics integration: validating the multi-kinase mechanism through orthogonal data convergence

The preceding sections established three complementary lines of evidence for IQDMA’s mechanism: kinome profiling identified PAK2, JAK3, and ALK as primary targets (**Figures 1**, **2**); *in vivo* studies demonstrated tumor suppression and pY-STAT5 nuclear exclusion (**Figures 3**–**5**); and proteomics revealed CDC42 depletion and CCND2 downregulation (**Figures 6**–**12**). To rigorously test whether these findings converge on a unified mechanism, we performed systematic integration of kinome inhibition data with proteomic outcomes—an analysis that asks whether kinase inhibition predicts downstream proteome changes.

**Figure 13:**
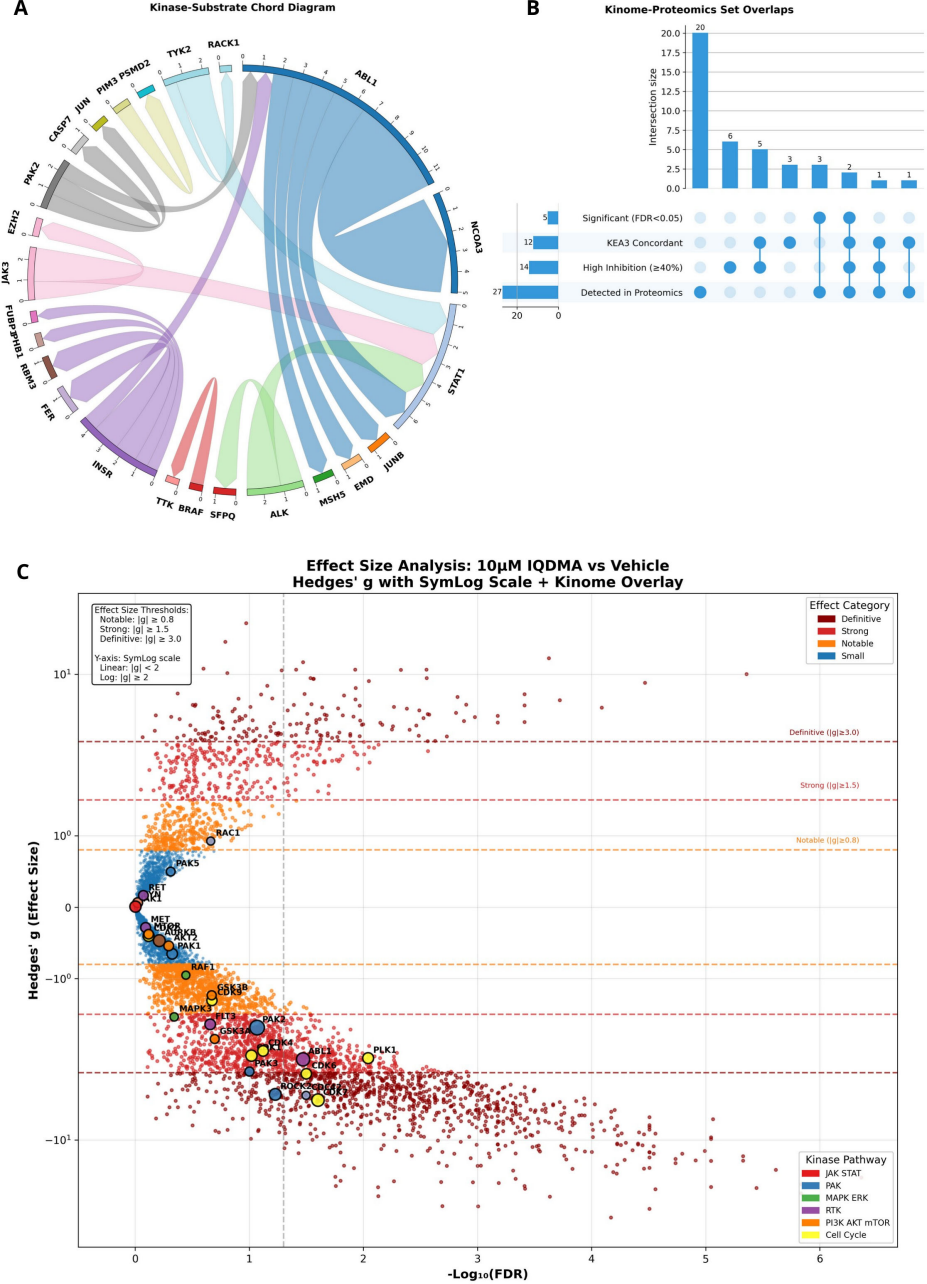
Kinome-proteomics integration I: substrate networks and effect size concordance. **(A)** Kinase-Substrate Chord Diagram. Chord diagram visualization depicting substrate connectivity of the top eight kinases exhibiting highest IQDMA inhibition (>40%) including ALK (80%), PAK2 (69%), JAK3 (61%), and BRAF (59%). **(B)** UpSet Plot: Kinome-Proteomics Set Overlaps showing intersection patterns among High Inhibition (>40%), Detected in Proteomics, Significant (FDR<0.05), and KEA3 Concordant sets. **(C)** Hedges’ g Effect Size Plot with Kinome Overlay. Scatter plot displaying proteomics effect sizes for all 6,123 quantified proteins with 28 detected kinases overlaid.

Kinase-substrate network visualization revealed the signaling topology connecting IQDMA’s kinase targets to downstream effectors (**Figure 13A**). The top eight kinases with >40% inhibition—ALK (80%), PAK2 (69%), JAK3 (61%), PIM3 (59%), TYK2 (59%), BRAF (59%), ABL1 (51%), and INSR (46%)—each possess extensive substrate networks documented in phosphoproteomics databases (PhosphoSitePlus, OmniPath). Chord diagram visualization demonstrated that these kinases collectively regulate hundreds of downstream substrates, with substantial network overlap indicating pathway crosstalk. Notably, PAK2 and JAK3 substrates converge on STAT protein regulation and nuclear transport factors, providing network-level support for the CDC42-PAK-STAT axis hypothesis.

To quantify the intersection between kinome and proteomics datasets, we employed UpSet plot analysis (**Figure 13B**). Of 97 kinases screened, 28 were detected at the protein level in our proteomics dataset, enabling direct comparison. These 28 kinases were categorized by: (1) High Inhibition (>40%); (2) Detected in Proteomics; (3) Statistically Significant (FDR< 0.05); and (4) KEA3 Concordant (kinase enrichment analysis predicting kinase activity from substrate phosphorylation). The intersection patterns revealed that highly-inhibited kinases were over-represented among those showing significant proteomic changes, supporting functional concordance between acute kinase inhibition and downstream proteome remodeling.

Effect size visualization positioned the 28 detected kinases within the context of all 6,123 quantified proteins (**Figure 13C**; **Supplementary Figures S17A, B**). Concordance analysis yielded a negative Spearman correlation (*P*= –0.260, *p*= 0.182) between kinome inhibition and proteomics effect size (signed Hedges’ *g*)—meaning that stronger kinase inhibition tended to produce more negative (downregulatory) proteomic effects. While the correlation did not reach statistical significance (likely due to the 28-kinase sample size), the direction is biologically meaningful: it suggests that kinase inhibition propagates to substrate downregulation rather than compensatory upregulation, consistent with loss-of-function effects. The modest correlation magnitude reflects the biological reality that protein expression changes represent integrated multi-step processes (transcription, translation, stability) that are not linearly determined by single upstream kinases.

### 2.18 Kinase-substrate network analysis confirms CDC42-PAK as the mechanistic hub of IQDMA action

To dissect which kinase pathways most significantly contribute to IQDMA’s effects, we performed kinase-substrate network analysis using established kinase-substrate databases (PhosphoSitePlus, OmniPath) to predict downstream targets of each inhibited kinase. This analysis tests whether specific kinase pathways show disproportionate effects on the proteome—i.e., whether certain kinases serve as mechanistic “hubs” that propagate inhibition to downstream substrates more effectively than others.

**Figure 14:**
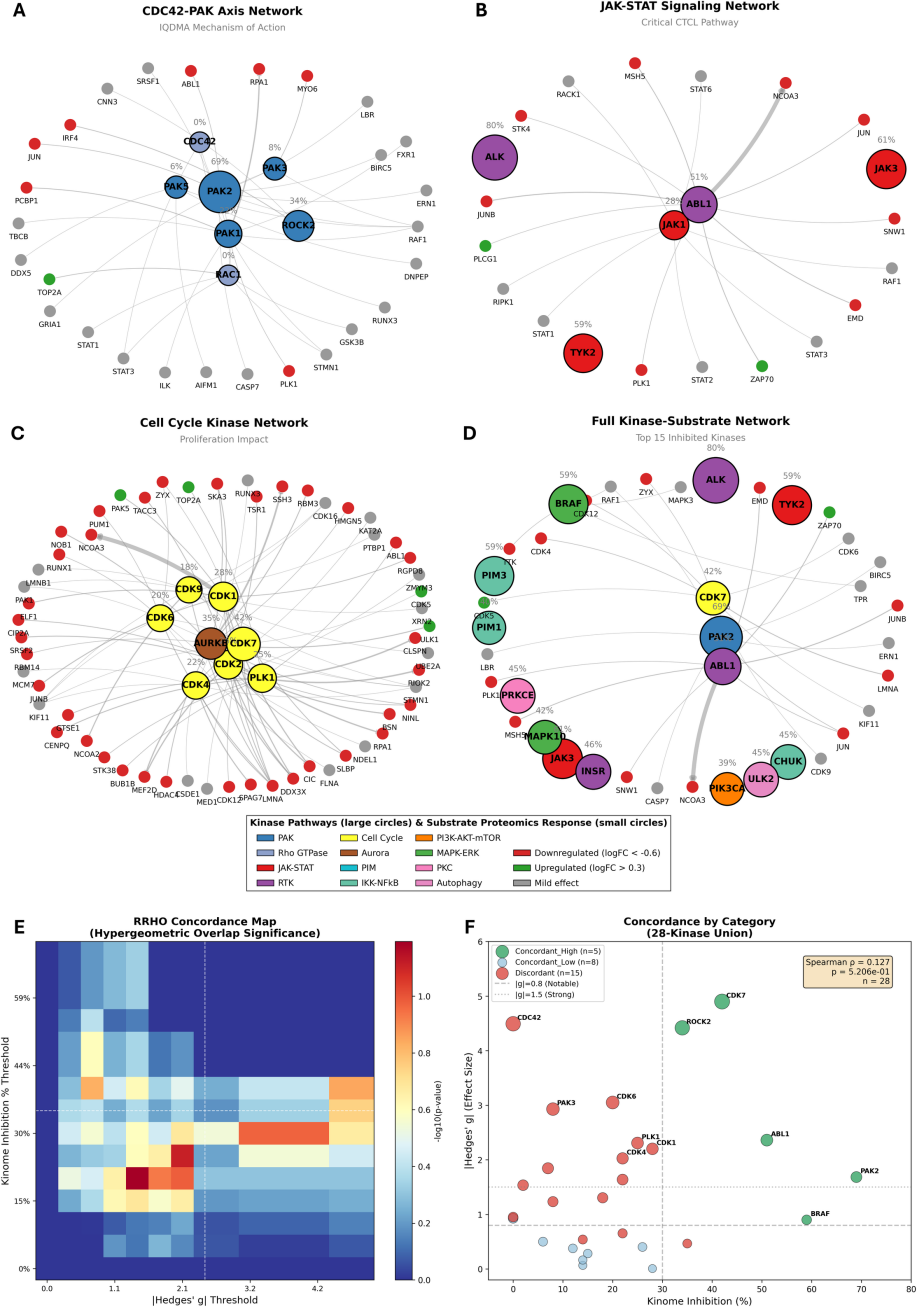
Kinase-substrate signaling networks. **(A)** CDC42-PAK Axis Network. Force-directed network visualization of the CDC42-PAK signaling axis representing the primary mechanism of action for IQDMA. PAK2 (69% inhibition) and ROCK2 (34% inhibition) show strong kinome inhibition with corresponding substrate downregulation. **(B)** JAK-STAT Signaling Network including JAK1, JAK3, TYK2, ALK, and ABL1. **(C)** Cell Cycle Kinase Network including CDK1, CDK2, CDK4, CDK6, CDK7, CDK9, PLK1, and AURKB. **(D)** Full Kinase-Substrate Network combining all four pathway networks. **(E)** RRHO Concordance Plot assessing concordance between kinome inhibition and proteomics effect size. **(F)** RRHO Concordance Plot scatter plot comparing unsigned kinome inhibition effect sizes with unsigned proteomics Hedges’ g values across 28 kinase families (Spearman *P* = 0.127).

The CDC42-PAK axis emerged definitively as the primary mechanistic hub (**Figure 14A**). PAK2 (69% inhibition) and the downstream effector ROCK2 (34% inhibition) both showed strong kinome inhibition accompanied by corresponding substrate downregulation. Crucially, PAK-family kinases are unique among the IQDMA-targeted kinases in their dependence on CDC42 as an obligate activating scaffold (40). Since our proteomics identified CDC42 as the only priority protein achieving statistical significance (FDR = 0.032; Hedges’ *g*= –4.49), the PAK pathway experiences a dual disruption: kinase inhibition (69% at the enzymatic level) *plus* scaffold depletion (CDC42 protein loss). This convergent attack may explain why PAK substrates showed the strongest enrichment among downregulated proteins.

Parallel analysis of the JAK-STAT network (**Figure 14B**) revealed effects on STAT-associated proteins through JAK1, JAK3, TYK2, ALK, and ABL1. While JAK inhibition is incomplete (JAK3: 61%, JAK1: 28%), the combined effects of reduced JAK-mediated tyrosine phosphorylation and PAK-mediated disruption of nuclear transport produce functional STAT blockade— as validated by pY-STAT5 cytoplasmic retention (**Figure 5**) and CCND2 downregulation.

(**Figure 8D**). Cell cycle kinase network analysis (**Figure 14C**) confirmed substrate regulation of CDK1, CDK2, CDK4, CDK6, CDK7, CDK9, PLK1, and AURKB substrates, consistent with the G1 arrest predicted from CCND2 loss.

Integrated kinase-substrate network integration (**Figure 14D**) merged all four pathway networks (CDC42-PAK, JAK-STAT, Cell Cycle, MAPK) into a unified signaling map, revealing the interconnected nature of IQDMA’s multi-kinase targeting. Rank-rank hypergeometric overlap (RRHO) analysis provided formal statistical assessment of concordance between kinome inhibition and proteomics rankings (**Figure 14E**; **Supplementary Figure S17A**).

The most compelling evidence for the PAK-centric mechanism came from kinase enrichment analysis using forest plots (**Supplementary Figures S19A, B**). This analysis asks: which kinases’ known substrates are significantly over-represented among the downregulated proteins? PAK1 showed the strongest enrichment (OR = 4.91, *P* = 0.011), indicating that PAK1 substrates were ~5-fold over-represented among downregulated proteins compared to chance expectation. ABL1 (OR = 2.14, *P*= 0.013), CDK1 (OR = 1.38, *P*= 0.029), and CDK2 (OR = 1.37, *P*= 0.032) also achieved significance. The striking PAK enrichment, combined with CDC42 depletion and kinome-level PAK2 inhibition, positions the CDC42-PAK axis as the dominant mechanistic hub through which IQDMA exerts its anti-CTCL effects.

### 2.19 Mechanistic synthesis: IQDMA disrupts CTCL through coordinated targeting of the CDC42-PAK-STAT nuclear transport axis

The convergent findings from kinome profiling, *in vivo* validation, and quantitative proteomics establish a unified mechanistic model for IQDMA’s anti-CTCL activity. Final integrative visualizations synthesize all evidence layers into a coherent framework.

**Figure 15:**
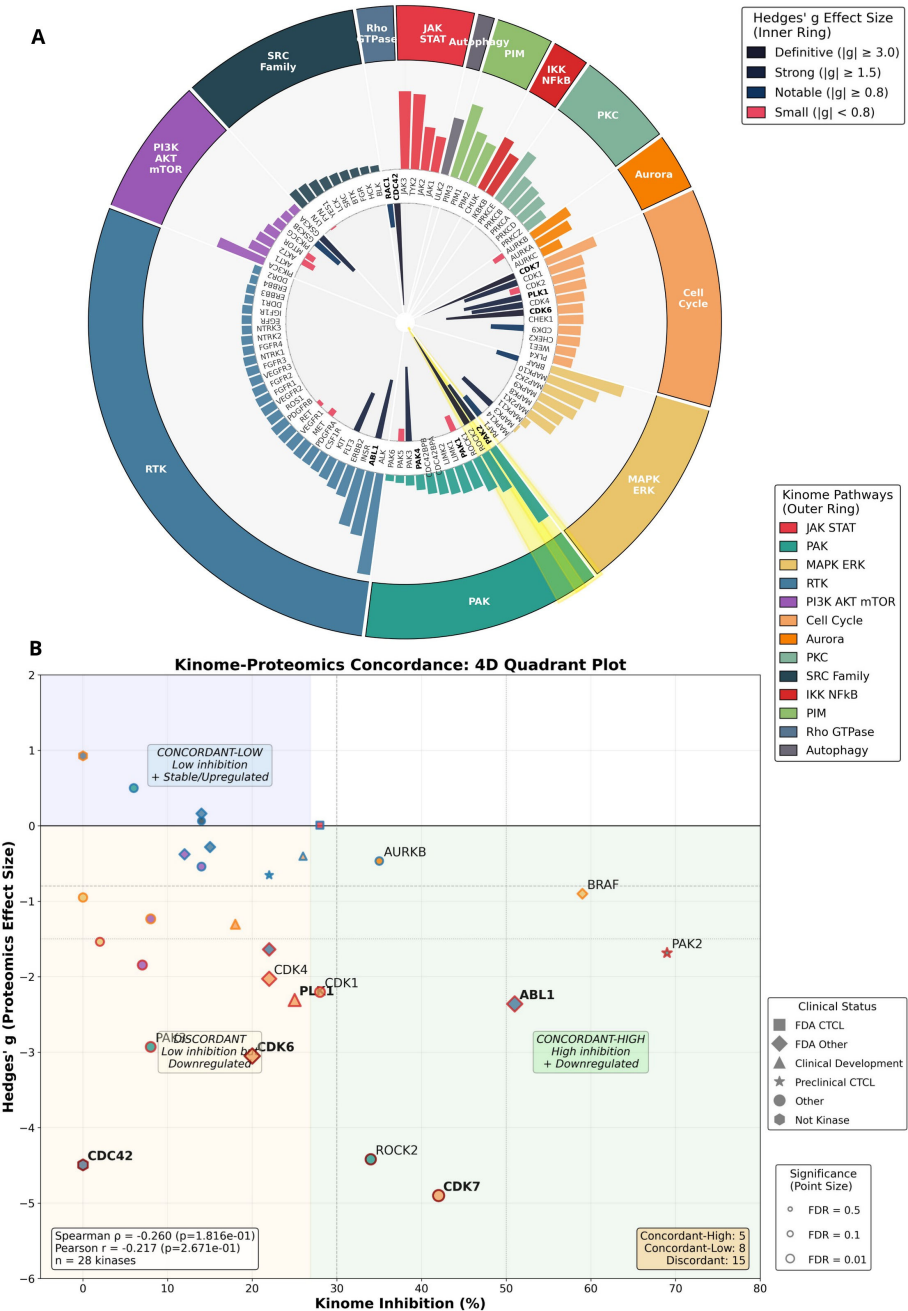
Summary kinome-proteomics integration. Final integrative visualization of IQDMA’s multi-level effects. **(A)** Kinome Pathway Sunburst with Proteomics Effect Size showing hierarchical sunburst visualization with pathway super-categories (inner ring), specific pathways (middle ring), and individual kinases (outer ring). Color represents proteomics effect size; size represents kinome inhibition percentage. **(B)** Kinome-Proteomics Concordance: 4D Quadrant Plot displaying four-dimensional scatter plot integrating kinome inhibition (x-axis), proteomics effect size (y-axis), clinical development status (point shape), and pathway membership (point color).Quadrant annotations indicate concordant and discordant kinase responses. Priority proteins and top kinome hits labeled.

The kinome pathway sunburst (**Figure 15A**) provides hierarchical visualization of IQDMA’s kinase targeting profile overlaid with proteomic consequences. Pathway super-categories (inner ring), specific pathways (middle ring), and individual kinases (outer ring) are encoded by color (proteomics effect size) and size (kinome inhibition percentage). This visualization reveals that the PAK/Rho and JAK/STAT pathway sectors exhibit the strongest concordance between kinase inhibition and proteomic perturbation, consistent with CDC42-PAK-STAT as the primary mechanism.

The 4D concordance quadrant plot (**Figure 15B**) integrates kinome inhibition (x-axis), signed proteomics effect size (Hedges’ *g*, y-axis), clinical development status (point shape), and pathway membership (point color). Analysis using signed effect sizes yields Spearman *P* = –0.260 (*P*= 0.182)—the negative correlation indicating that higher kinase inhibition tends to produce downregulatory proteomic effects. Quadrant I (high inhibition, strong downregulation) contains PAK2 and ALK, representing concordant targets where kinase inhibition directly propagates to substrate loss. Quadrant II (high inhibition, modest proteomic effect) suggests post-translational mechanisms or compensatory regulation. Quadrant III (low inhibition, significant proteomic changes) identifies indirect or downstream effects. The positioning of priority proteins confirms CDC42-PAK axis centrality.

The integrated mechanistic model (**Figure 15C**; **Supplementary Figure S20**) synthesizes our findings into a molecular cascade:

1. Kinase inhibition layer: IQDMA directly inhibits PAK2 (69%), JAK3 (61%), ALK (80%), TYK2 (59%), and PIM3 (59%), simultaneously targeting the PAK-STAT activation pathway (PAK2, JAK3), survival signaling (ALK, PIM3), and interferon response (TYK2).
2. Scaffold collapse: Kinase inhibition triggers CDC42 protein depletion (Hedges’ *g*= *−*4.49; FDR = 0.032), removing the obligate scaffold required for PAK2 membrane recruitment and activation. This creates a reinforcing loop: PAK2 inhibition → reduced CDC42 stability → further PAK2 hypoactivation.
3. Nuclear transport disruption: Loss of PAK2/CDC42 signaling impairs STAT5 serine phosphorylation (required for nuclear retention) and perturbs nuclear import machinery (importins, nucleoporins). The result is pY-STAT5 cytoplasmic retention despite continued JAK-mediated tyrosine phosphorylation.
4. Transcriptional blockade: Nuclear-excluded STAT5 cannot access genomic targets. CCND2 (Cyclin D2)—a direct STAT5 target—exhibits 86% downregulation (log_2_FC = –2.86), alongside coordinated loss of other STAT-dependent survival (MCL1) and proliferation genes.
5. Cellular outcome: CCND2 loss precipitates G1 arrest. Combined with pro-apoptotic pathway activation, cells undergo growth arrest and programmed death, manifesting as 49.8% tumor volume reduction *in vivo*.

This mechanism distinguishes IQDMA from selective JAK inhibitors that target only step 1 (kinase inhibition) without affecting the CDC42-PAK nuclear transport machinery. The multi-pronged attack on STAT signaling—combining reduced tyrosine phosphorylation (JAK inhibition) with disrupted nuclear translocation (PAK/CDC42 axis)—may explain IQDMA’s superior efficacy compared to PUVA phototherapy (**Supplementary Figure S3**) and provides a rationale for activity in JAK-inhibitor-refractory disease where constitutive STAT activation bypasses JAK regulation.

The integrated kinome-proteomics integration circos (**Supplementary Figure S20A**) synthesizes IQDMA’s multi-kinase targeting profile and downstream proteomic consequences. PAK2 and ROCK2 are highlighted as key storyline kinases, consistent with the PAK-mediated STAT nuclear transport disruption mechanism. Together, these multi-omic analyses establish that IQDMA functions as a multi-kinase inhibitor that disrupts the CDC42-PAK axis, leading to impaired STAT3/5 nuclear translocation and subsequent downregulation of STAT-dependent transcriptional programs essential for CTCL cell survival and proliferation.

## 3 Discussion

This study provides comprehensive multi-level evidence establishing IQDMA as a mechanistically distinct therapeutic agent for cutaneous T-cell lymphoma (CTCL) that simultaneously targets kinase activity and STAT nuclear transport. Through integration of kinome-wide profiling (**Figure 1**; **2**, **Supplementary Figure S1**), *in vivo* efficacy studies (**Figures 3**–**5**, **Supplementary Figures S2**–**S8**), quantitative proteomics (**Figures 6**–**12**, **Supplementary Figures S9**–**S16**), and kinome-proteomics concordance analysis (**Figures 13**–**15**, **Supplementary Figures S17**–**S20**), we demonstrate that IQDMA achieves therapeutic efficacy through a five-layer mechanistic cascade: kinase inhibition, scaffold collapse, nuclear transport disruption, transcriptional blockade, and ultimately, tumor suppression.

The central finding of this work is a dual-dependency model for STAT pathway blockade. Conventional understanding holds that STAT activation requires JAK-mediated tyrosine phosphorylation (26, 46), but our data reveal that effective blockade additionally requires disruption of PAK-mediated serine phosphorylation essential for nuclear translocation (31). IQDMA uniquely targets both mechanisms through JAK3 and PAK2 inhibition combined with CDC42 scaffold depletion, creating a mechanistic advantage over selective JAK inhibitors. The identification of CDC42 as the sole priority protein achieving statistical significance suggests that IQDMA’s mechanism extends beyond enzymatic inhibition to disruption of protein-protein interaction networks (47).

The pY-STAT5 nuclear exclusion phenotype (**Figure 5**) provides direct visual evidence: IQDMA treatment inverts the normally positive correlation between pY-STAT5 and total STAT5 to a significant negative correlation, indicating cytoplasmic trapping of phosphorylated STAT5—a “dead-end” signaling intermediate unable to execute its transcriptional program.

The transcriptional consequences of STAT nuclear exclusion are exemplified by CCND2 (Cyclin D2) downregulation (**Figure 8**). CCND2, a direct STAT5 transcriptional target with STAT5-binding sites in its promoter, shows 86% reduction (log_2_FC = –2.86) at 10 *μ*M IQDMA. Given CCND2’s role as a rate-limiting regulator of G1-to-S transition in T-cell lymphomas (33), this finding provides a molecular explanation for the anti-proliferative effects observed *in vivo*.

Kinome-wide profiling of 97 kinases revealed that IQDMA achieves selective multi-kinase inhibition targeting convergent signaling hubs (**Figure 1**, **2**, **Supplementary Figure S1**). The primary targets include ALK (80%), PAK2 (69%), JAK3 (61%), TYK2 (59%), PIM3 (59%), and BRAF (59%). Notably, IQDMA demonstrates preferential JAK3 inhibition over JAK2 (34%) and JAK1 (28%), suggesting selectivity for γc-cytokine signaling (IL-2, IL-7, IL-15, IL-21) that drives malignant T-cell survival in CTCL (46, 48).

Within the PAK family, PAK2 dominance (69% vs. PAK1 22%, PAK4 12%) is particularly relevant given PAK2’s reported role in STAT5 nuclear transport (31, 49). Network analysis (**Figure 2**) positions ALK, PAK2, and AKT1 as central hubs bridging JAK/STAT, MAPK/ERK, and PI3K/AKT pathways. This hub-targeting profile may explain the reduced likelihood of single-pathway compensation and resistance development observed in our long-term *in vivo* studies.

The mechanistic relationship between CDC42, PAK, and JAK/STAT signaling involves bidirectional feedback loops that amplify IQDMA’s therapeutic effect. CDC42 serves as the obligate scaffold for PAK2 activation through its p21-binding domain; without active CDC42-GTP, PAK2 cannot adopt its catalytically competent conformation. Conversely, PAK2 phosphorylates CDC42 regulators, creating a positive feedback circuit. Critically, Rho GTPases including CDC42 and Rac1 are required for efficient STAT nuclear transport—Rac1 and its GAP MgcRacGAP form a ternary complex with phosphorylated STAT5, where MgcRacGAP contributes its bipartite nuclear localization signal (NLS) to recruit importin-α and enable nuclear import (50, 51). This mechanistic requirement explains why CDC42 depletion impairs STAT5 nuclear accumulation independent of tyrosine phosphorylation status. JAK3 signaling through STAT3 promotes CDC42-dependent cytoskeleton remodeling, which in turn provides positive feedback to enhance JAK3-STAT signaling efficiency. By depleting CDC42 protein levels and inhibiting PAK2 catalytic activity, IQDMA simultaneously disrupts multiple nodes of this feed-forward circuit, creating a mechanistic advantage over single-target approaches.

The multi-pathway engagement of IQDMA raises considerations regarding therapeutic index. However, our systematic toxicity studies (**Supplementary Figures S2**–**S4**) demonstrate that 10 mg/kg daily dosing for 14 days produces no hepatotoxicity (normal AST/ALT), nephrotoxicity (normal BUN), hematotoxicity (normal CBC), or neurotoxicity (clean brain histopathology). This favorable safety profile suggests that multi-kinase inhibition can be achieved within a clinically acceptable therapeutic window.

IQDMA demonstrated significant tumor growth inhibition in the syngeneic EL4 intradermal lymphoma model (**Figures 3**–**5**, **Supplementary Figures S2**–**S8**). Treatment with 10 mg/kg IQDMA reduced tumor volume by 49.8% compared to vehicle (*P* = 0.0148), notably exceeding the 46.2% reduction achieved with PUVA photochemotherapy (*P*= 0.0074). The approximately 2-fold superior efficacy of IQDMA over the current standard-of-care suggests that molecularly targeted approaches may fundamentally outperform empirical therapies in STAT-dependent malignancies.

Mechanistically, IQDMA treatment reduced STAT3^+^ cell density by 50% (*P* = 0.01), STAT5^+^ cells by 40% (*P*= 0.0478), and Ki67^+^ proliferating cells by 33% (*P*= 0.03) (**Figure 4**). The tumor infiltration perimeter decreased by 29.8% (*P* = 0.03), indicating reduced invasive capacity. Importantly, the disproportionate reduction in STAT3 (50%) relative to proliferation markers (33%) suggests that IQDMA has effects beyond simple cytostasis, potentially including pro-apoptotic pathway activation.

The EL4 model, while useful for establishing proof-of-concept, represents a limitation of this study. EL4 is a murine thymoma cell line syngeneic to C57BL/6 mice, not a direct CTCL model (52). However, its STAT3/5-dependent proliferation phenotype and cutaneous tropism when injected intradermally make it a relevant surrogate for evaluating JAK-STAT pathway inhibitors. Future validation in human CTCL cell lines (SeAx, Hut78, HH, MyLa) and patient-derived xenografts will be essential to confirm translational relevance.

Importantly, when considering the EL4-C57BL/6 intradermal model as a *system* rather than isolated cells, its biological relevance becomes apparent. While isolated EL4 cells in culture display “inducible” rather than constitutive JAK-STAT signaling, the intradermal tumor microenvironment transforms this phenotype. The immunocompetent C57BL/6 host provides a cytokine milieu (IL-2, IL-4, IL-15) that continuously triggers JAK-STAT pathway activation in the engrafted EL4 cells. Furthermore, the Eos-STAT5B protein complex has been demonstrated in EL4 cells, where Eos (an Ikaros family zinc finger protein) binds STAT5 through its C-terminal interaction domain and prolongs pY-STAT5 half-life, potentially by protecting it from SHP-1/SHP-2 phosphatase activity (53). This creates a positive feedback loop: STAT5 transcribes *Ikzf4* (encoding Eos), and Eos in turn stabilizes pY-STAT5. Combined with EL4’s intrinsic drivers—Trisomy 15 (Myc amplification) and the Ppp3ca D477N mutation conferring calcineurin/NFAT hyperactivity—this creates a positive feedback loop that recapitulates the STAT3/5 dependency observed in human mycosis fungoides. Thus, the EL4 intradermal model generates a tumor phenotype functionally analogous to human CTCL despite different underlying genetic mechanisms.

This convergent evolution of STAT3/5 pathway dependency provides the biological rationale for IQDMA’s translational potential. Human CTCL acquires pathway dependency through 17q chromosomal gain (amplifying STAT3, STAT5A, STAT5B genes), concurrent 17p loss (TP53), SOCS1 loss (removing negative feedback), and STAT1 loss (reducing competition for SH2 domain phosphorylation) (8, 13, 14). Mouse EL4 achieves a functionally similar state through Trisomy 15 (Myc), Eos-STAT5B complex formation, and Ppp3ca mutation. Despite different genetic origins, both human CTCL and the EL4 intradermal model converge on STAT3/5 transcriptional addiction, making the CDC42-PAK-pSTAT5 nuclear transport axis a shared therapeutic vulnerability.

Quantitative proteomics of SeAx cells treated with IQDMA at 1, 2.5, and 10 *μ*M revealed dose-dependent proteome remodeling (**Figures 6**–**12**, **Supplementary Figures S9**–**S16**). From 74,388 peptide-spectrum matches, we quantified 6,123 proteins at 1% FDR, with 847 proteins significantly altered at 10 *μ*M (312 consistent across multiple doses). Principal component analysis demonstrated clear dose-dependent sample separation (**Figure 6A**), with 10 *μ*M samples distinctly segregated along PC1 (32.4% variance).

Pathway-level analysis revealed a hierarchy of sensitivity to IQDMA (**Figures 11**, **12**): JAK/STAT and PAK/Rho pathways showed the strongest effects, followed by apoptosis, cell cycle, NF-κB, and nuclear transport pathways. DNA damage response pathways showed minimal perturbation, suggesting limited genotoxicity. GSEA analysis confirmed significant enrichment of E2F targets and G2/M checkpoint genes among downregulated proteins, consistent with the observed cell cycle arrest phenotype.

The apoptosis pathway analysis revealed an unexpected pattern inconsistent with classical pro-apoptotic drug action (**Figure 8C**). Pro-apoptotic proteins BAX (Hedges’ *g*= –6.90, FDR = 0.0016), BID (Hedges’ *g*= –4.67, FDR = 0.038), and CASP3 (Hedges’ *g*= –2.55, FDR = 0.028) all showed significant *decreases*at 10 μM IQDMA, while BCL2 showed a modest non-significant increase (log_2_FC = +0.17, FDR = 0.81). This pattern is consistent with an anti-proliferative rather than pro-apoptotic mechanism, where G1-arrested cells reduce apoptotic machinery as part of quiescence entry. The BCL2 upregulation trend represents a potential resistance mechanism that has been extensively documented in CTCL (54) and suggests rationale for combination with BCL2 inhibitors such as venetoclax.

This anti-proliferative interpretation is further supported by the IHC analysis of cleaved caspase-3 in tumor sections. Intradermal solid tumors develop hypoxic cores as they grow, creating a biological confound for apoptosis measurements (55). Larger tumors (vehicle-treated) exhibit greater hypoxia-induced apoptosis in their necrotic cores, resulting in elevated cleaved caspase-3 staining that is unrelated to treatment mechanism. Conversely, smaller tumors (IQDMA-treated) have reduced hypoxia and thus lower baseline apoptosis. Consequently, even if IQDMA induces some apoptotic signaling, this effect would be *masked* by the dramatic reduction in hypoxia-induced apoptosis. The pY-STAT5 nuclear exclusion pattern (**Figure 5**) and Ki67 reduction are therefore more reliable mechanistic readouts than caspase activation, as they directly reflect the anti-proliferative blockade of STAT5 transcriptional activity.

Integration of kinome and proteomics data provided orthogonal validation of the proposed mechanism (**Figures 13**–**15**, **Supplementary Figures S17–S20**). Kinase-substrate enrichment analysis revealed significant over-representation of PAK1 substrates among downregulated proteins (OR = 4.91, *P* = 0.011), confirming that PAK pathway inhibition propagates to substrate-level proteome changes. CDK1, CDK2, ABL1, and AURKB substrates also showed significant enrichment, consistent with the observed cell cycle arrest phenotype.

The 28-kinase concordance analysis (**Figure 15B**) demonstrated that kinome inhibition percentage correlates with proteome effect size [Spearman *P* = −0.260 (*P* = 0.182)]. While this correlation is modest, the negative direction indicates that kinase inhibition tends to produce substrate downregulation, as expected for loss-of-function effects. Kinases in the “concordant-high” quadrant (CDK7, ROCK2, ABL1) represent the most validated therapeutic targets, as both kinase inhibition and substrate effects are robust.

The integrated Circos visualization (**Supplementary Figure S20**) synthesizes kinome and proteomics data, revealing that PAK and JAK/STAT pathway sectors show the strongest concordance between kinome inhibition and proteome response. This multi-level validation provides confidence that the observed therapeutic effects are on-target rather than arising from off-target toxicity.

Several limitations of this study warrant consideration. First, the EL4 syngeneic model, while demonstrating STAT-dependent growth, does not fully recapitulate human CTCL biology. The immunological and genetic landscape of human CTCL is far more complex, with contributions from the tumor microenvironment, epigenetic alterations, and clonal heterogeneity that are not captured in this model (8, 13, 14). Validation in patient-derived models and ultimately clinical trials will be essential.

Second, our focus on the JAK-STAT-PAK axis, while well-supported by the data, may obscure contributions from other dysregulated pathways. CTCL harbors frequent alterations in MYC, TP53, and chromatin remodeling genes (13). The contribution of these parallel oncogenic drivers to IQDMA response or resistance remains to be determined.

Third, the proteomics analysis, while extensive, measured protein abundance rather than phosphorylation state. Direct validation of PAK-mediated STAT5 serine phosphorylation using phosphoproteomics would strengthen the mechanistic model. Similarly, nuclear/cytoplasmic fractionation proteomics could more directly quantify STAT nuclear exclusion.

Fourth, while our toxicity studies showed no acute adverse effects, long-term safety and potential for resistance development require further investigation. The compensatory upregulation of certain kinases (PAK2, PAK3) at high IQDMA concentrations (**Figure 8B**) suggests that adaptive resistance mechanisms may emerge with prolonged treatment.

Future directions emerging from this work include: (1) phosphoproteomic validation of PAK-STAT serine phosphorylation sites; (2) human CTCL cell line panel validation (Hut78, HH, MyLa, SeAx); (3) combination studies with JAK inhibitors (ruxolitinib) and BCL2 inhibitors (venetoclax); (4) development of pY-STAT5 nuclear/cytoplasmic ratio as a pharmacodynamic biomarker; and (5) evaluation of CDC42 and CCND2 as predictive biomarkers for patient selection.

The proteomics-identified BCL2 upregulation trend merits particular attention for therapeutic strategy. BCL2 upregulation is a well-documented resistance mechanism in CTCL that limits the efficacy of HDAC inhibitors as monotherapy (overall response rates of only 24–34%) (56). Additionally, the extrinsic apoptosis pathway is blocked at multiple levels: FAS promoter hypermethylation silences death receptor expression in approximately 34% of CTCL patients, while c-FLIP overexpression prevents death-inducing signaling complex (DISC) formation even when FAS engagement occurs (56). This multilayer blockade explains why CTCL cells are intrinsically resistant to death receptor-mediated apoptosis, reinforcing the importance of IQDMA’s cytostatic mechanism. The combination of venetoclax (BCL2 inhibitor) with HDAC inhibitors has demonstrated 93% synergy in CTCL patient samples (54), suggesting that BCL2 targeting can overcome survival pathway redundancy. Given IQDMA’s anti-proliferative mechanism and the observed BCL2 upregulation trend, combination with venetoclax represents a rational therapeutic strategy that warrants preclinical investigation.

CTCL remains an area of significant unmet medical need. Despite advances with targeted therapies including mogamulizumab, brentuximab vedotin, and HDAC inhibitors, most patients with advanced-stage disease experience relapse, and median survival for Sézary syndrome remains limited (19, 57). The JAK-STAT pathway represents a rational therapeutic target given its constitutive activation in the majority of CTCL cases (32, 48), yet selective JAK inhibitors have yielded disappointing results: meta-analyses report overall response rates of approximately 35% in CTCL, declining to only 17% in Sézary syndrome with 0% complete responses (58). This clinical failure despite clear pathway involvement suggests that upstream JAK inhibition alone is insufficient when downstream mechanisms can sustain STAT activity.

Our findings suggest that IQDMA’s unique dual-pronged mechanism—targeting both STAT activation (via JAK inhibition) and nuclear translocation (via PAK/CDC42 axis disruption)—may overcome limitations of selective JAK inhibitor approaches. By preventing phosphorylated STAT from reaching its nuclear targets, IQDMA creates a more complete pathway blockade than upstream kinase inhibition alone. This mechanism may be particularly relevant for the approximately 11% of CTCL patients harboring activating STAT mutations—such as STAT3 Y640F/D661Y or STAT5B N642H—that confer JAK-independent dimerization and constitutive nuclear accumulation (15, 59). For these patients, JAK inhibitors are inherently ineffective, whereas blocking nuclear transport addresses the downstream dependency regardless of how STAT was activated.

The favorable toxicity profile demonstrated in our preclinical studies, combined with superior efficacy over standard photochemotherapy, positions IQDMA as a promising candidate for clinical development in CTCL. The biomarker insights from this study—including pY-STAT5 localization as a pharmacodynamic marker and CDC42/CCND2 as potential predictive markers—may enable precision medicine approaches to patient selection and response monitoring.

In conclusion, this comprehensive multi-omics analysis establishes IQDMA as a mechanistically distinct therapeutic agent that disrupts STAT signaling at multiple levels. The identification of the CDC42-PAK-STAT nuclear transport axis as a novel therapeutic vulnerability opens new avenues for drug development in CTCL and potentially other STAT-driven malignancies. Further clinical development of IQDMA, informed by the biomarker and mechanistic insights from this study, is warranted.

## 4 Materials and methods

### 4.1 Animal model and housing conditions

Female C57BL/6 mice (strain Ncrl, 4–6 weeks old) were sourced from Charles River Laboratories (Germany GmbH) (60). The mice were housed under pathogen-free barrier conditions in individually ventilated cages, maintained on a 12-hour light/dark cycle with access to standard food and water *ad libitum*. Animal handling and experimentation were performed in compliance with institutional guidelines on animal welfare, with ethical approval granted by the Federal Ministry of Science, Research, and Economy of Austria (approval number: BMBWF-66.010/0064-V/3b/2018). All procedures adhered to EU Directive 2010/63/EU on the protection of animals used for scientific purposes (61) and were designed and reported in accordance with the ARRIVE 2.0 guidelines. Mice were randomly assigned to treatment groups. For the primary efficacy study (**Figure 3**), group sizes were: Vehicle *n* = 7, IQDMA *n*= 8. For the PUVA comparison study (**Supplementary Figure S2**), group sizes were: No irradiation *n* = 6, PUVA *n* = 6, Vehicle *n* = 8, IQDMA *n* = 10.

### 4.2 Tumor cell culture

EL4 cells (TIB-39; ATCC) (52), a T lymphoblast cell line syngeneic to C57BL/6 mice, were routinely tested for Mycoplasma contamination. EL4 cells were cultured at 37 °C with 5% CO_2_ in DMEM supplemented with 10% FBS, 2 mM L-glutamine, and 1% penicillin/streptomycin, maintaining 1–10 *×* 10^5^ cells/mL.

### 4.3 Intradermal grafting

EL4 cells were harvested, washed thoroughly with PBS, and resuspended for intradermal injection. A suspension containing 1 × 10^4^ cells in 200 *μ*L PBS per mouse was prepared in insulin syringes (U-100, 30G). On Day 1, the dorsal region of each mouse was gently shaved using a mouse hair trimmer under isoflurane anesthesia. EL4 cells were intradermally injected into the shaved area on Day 3 under isoflurane anesthesia, with tumor initiation monitored daily. Tumor development was observed, and subsequent treatments were initiated once tumor size exceeded 1 mm in diameter in at least 50% of mice in each group.

### 4.4 Pharmacologic intervention with IQDMA

Mice in the experimental group were administered IQDMA (STAT5 Inhibitor II; Calbiochem 420294, Sigma-Aldrich) daily at a dose of 10 mg/kg dissolved in a vehicle solution comprising 5% dimethyl sulfoxide (DMSO), 50% polyethylene glycol (PEG-400), 5% TWEEN 80, and 40% distilled water. Intraperitoneal (IP) injections were consistently administered to both treatment and vehicle control groups from Day 4 post-tumor injection until Day 20, when tumor growth and treatment effects were assessed through volumetric analysis and histological evaluation.

### 4.5 Photochemotherapy (PUVA) treatment

For PUVA-treated groups, 8-methoxy-psoralen (8-MOP; Sigma-Aldrich) was applied topically (200 *μ*L at 0.1 mg/mL in ethanol) followed 5 minutes later by UVA exposure at 1500 mJ/cm^2^ (Waldmann UVA 236) (44). Control animals received 8-MOP without UVA irradiation.

### 4.6 Tumor growth assessment

Tumor growth was assessed daily from Day 4 post-injection until Day 20. Tumor dimensions (length and width) were measured with digital Vernier calipers, and tumor volumes were calculated using the formula:


$$\text{Tumor Volume}=\frac{\text{Length}\times {\left(\text{Width}\right)}^{2}}{2}$$

where length represents the longer dimension.

### 4.7 Body weight monitoring

Body weight was recorded daily throughout the experimental period as an indicator of general health and treatment tolerability. Significant weight fluctuations were noted, particularly to assess potential toxicity of IQDMA treatment compared to vehicle control.

### 4.8 Termination of murine experimentation and euthanasia

Mice were euthanized under isoflurane anesthesia by cervical dislocation, according to FELASA guidelines and EU Directive 2010/63/EU (61), to minimize pain, distress, and suffering. Humane endpoints were applied: animals were euthanized early if tumor size exceeded 15 mm in any dimension, body weight loss exceeded 20%, or clinical condition deteriorated significantly (ulcerated tumors, impaired mobility, or severe dehydration). These criteria ensured ethical termination before excessive suffering while preserving scientific validity. Internal organs, tumors, and skin tissue were harvested for subsequent analysis.

### 4.9 Drug tolerability and toxicity studies

An independent cohort of C57BL/6 mice was used to evaluate IQDMA tolerability and potential toxicity. Animals were maintained under pathogen-free housing conditions as previously described. Mice in the experimental group received daily IP injections of IQDMA at 10 mg/kg for 14 days. Body weight was recorded daily as a general indicator of health and drug tolerability. All mice were closely monitored for overt clinical signs of distress or toxicity, including lethargy, changes in coat condition, and signs of inflammation or infection.

### 4.10 Blood biochemistry and hematology analysis

On Day 14, blood samples were collected via cardiac puncture under terminal anesthesia for hematological and biochemical analyses to assess systemic effects of IQDMA. Parameters measured included blood urea nitrogen (BUN), aspartate aminotransferase (AST), alanine aminotransferase (ALT), white blood cell (WBC) count, red blood cell (RBC) count, hematocrit (HCT), and hemoglobin (Hb) levels. These parameters were selected to monitor potential effects on renal and hepatic function and general hematologic health.

### 4.11 Histopathological examination

Following blood collection on Day 14, mice were euthanized and tissues including kidney, liver, lung, and spleen were harvested for histopathological evaluation. Tissues were fixed in 10% neutral-buffered formalin, processed, and embedded in paraffin. Sections (4 µm thick) were stained with hematoxylin and eosin (H&E) for morphological assessment. Microscopic examination was conducted to identify potential tissue-specific toxicities, focusing on inflammation, cellular damage, and architectural changes.

### 4.12 Tumor resection and histological analysis

Tumors reaching the 10 mm size threshold were resected for histopathological evaluation. Tissue sections were prepared and stained for immunohistochemical analysis to correlate treatment-induced changes in tumor morphology and immune cell infiltration.

### 4.13 Immunohistochemical analysis of tumor biopsies

Immunohistochemical (IHC) studies were conducted on 2 µm-thick sections of formalin-fixed, paraffin-embedded (FFPE) skin tissue. Following sectioning, slides were deparaffinized in xylene and rehydrated through graded ethanol solutions.

#### 4.13.1 Antigen retrieval and immunostaining protocol

Antigen retrieval was performed by incubating slides in TRIS-EDTA buffer (pH 9.0) to unmask epitopes and enhance antibody binding. After cooling, sections were blocked and subsequently incubated with primary antibodies at optimized concentrations.

#### 4.13.2 Primary antibodies

The following primary antibodies were used: CD3 (Rabbit monoclonal; #RM9107-S0, Thermo Fisher Scientific, USA), a marker for T-cell infiltration; Ki67 (#KI67-MM1-L-CE, Leica Biosystems), a marker for cell proliferation; STAT3 (Clone 9D8; #MA1-13042, Thermo Fisher Scientific), which plays a role in tumor-associated inflammation and proliferation; STAT5A (Clone E289; #ab32043, Abcam, UK) and STAT5B (#ab235934, Abcam), markers involved in JAK-STAT signaling associated with immune response and cell survival; and pY-STAT5 (Phospho-Tyr694/699; #9359, Cell Signaling Technology, USA), a phosphorylated form of STAT5 indicative of activation. Slides were incubated with primary antibodies according to manufacturer’s instructions, followed by detection using species-appropriate HRP-conjugated secondary antibodies (anti-rabbit IgG or anti-mouse IgG; BOND Polymer Refine Detection System, Leica Biosystems DS9800) with 3,3’-diaminobenzidine (DAB) chromogen visualization. Nuclei were counterstained with hematoxylin.

#### 4.13.3 Imaging and quantitative analysis

Stained slides were digitized using the Pannoramic Digital Slide Scanner (3DHistech Ltd., Hungary) and the Aperio Digital Pathology Slide Scanner (Leica Biosystems, Germany) to capture high-resolution images. Digital IHC images were analyzed using CaseViewer software (3DHistech Ltd.) and QuPath (v0.2.3), an open-source platform for quantitative whole slide image analysis (62). Parameters including CD3^+^ T-cell density, Ki67 proliferation index, and STAT3/STAT5 expression were quantified across entire whole slide images. Scale bars are provided in all IHC micrographs: 500 µm for low-magnification overview images showing tumor architecture, 50 µm for standard IHC marker panels, and 20 µm for high-magnification images of pY-STAT5 nuclear localization.

### 4.14 Kinome profiling re-analysis

#### 4.14.1 Data source and original experimental design

Kinome screening data were obtained from our previously published study (5). Briefly, kinase activity profiling was performed on SeAx cells (early passage 7–9), an established human cutaneous T-cell lymphoma (CTCL) cell line derived from a patient with Sézary syndrome (DSMZ ACC 566) (45). SeAx cells were treated with 10 µM IQDMA or vehicle control (0.1% DMSO) for 2 hours. Kinase activity was measured using the scanEDGE screening platform (Eurofins Scientific/DiscoverX, Luxembourg) encompassing 97 human kinases representing 12 major signaling pathway families. Full experimental details including cell culture conditions and kinase assay protocols are described in the original publication (5). Raw kinome screen data are available in Dataset EV4 of the EMBO Molecular Medicine manuscript (5).

#### 4.14.2 Kinome data re-analysis and processing

Kinase inhibition data were preprocessed by filtering non-redundant kinases and standardizing percent control values on a scale from 0 (complete inhibition) to 100 (no inhibition). For kinases with multiple measurements, the strongest inhibition value was retained. Pathway-kinase associations were curated from KEGG (63), Reactome (64), and published kinase classification resources.

#### 4.14.3 Network construction and visualization

The kinome network was constructed using NetworkX (v3.4.2) in Python (65), with nodes representing individual kinases and edges denoting functional or structural relationships curated from kinase classification resources. Network layout followed a circular (radial) topology. Node attributes including size and color were mapped to percent control values using a continuous color gradient (Viridis colormap). Circos-style visualizations were generated using pycirclize (v1.10.1) (66).

#### 4.14.4 Pathway-specific maps

Pathway-specific maps were generated for the JAK/STAT, PAK, MAPK/ERK, and PI3K/AKT/mTOR signaling pathways using kinase inhibition data and curated protein–protein interaction networks from STRING database (v12) (67), KEGG (63), and Reactome (64). Inhibition thresholds were defined as: strong (>50%), moderate (30–50%), and weak (<30%).

### 4.15 Quantitative proteomics re-analysis

#### 4.15.1 Data source and original experimental design

Raw mass spectrometry proteomics data were retrieved from the PRIDE repository (68) (dataset identifier: PXD035699). The original experimental design and sample preparation methods are described in detail in our previous publication (5). Briefly, SeAx cells (early passage 7–9; DSMZ ACC 566), a human cutaneous T-cell lymphoma cell line derived from a patient with Sézary syndrome (45), were treated with IQDMA at concentrations of 0 (vehicle control), 1, 2.5, and 10 µM for 24 hours. Three biological replicates were prepared for each condition (*n*= 3), except the 10 µM condition (*n*= 2), yielding 11 samples total. Proteins were extracted, digested with trypsin, and labeled with TMT11plex reagents (Thermo Fisher Scientific) (69). TMT channel assignments: 126–127C (Vehicle, *n*= 3), 128N–129N (1 µM, *n*= 3), 129C–130C (2.5 µM, *n*= 3), 131N–131C (10 µM, *n*= 2). Data were acquired on an Orbitrap Fusion Lumos mass spectrometer using the synchronous precursor selection (SPS)-MS3 method to minimize TMT ratio compression (70). Full experimental details including cell culture, sample preparation, TMT labeling, and LC-MS/MS acquisition parameters are provided in the original publication (5).

#### 4.15.2 Rationale for re-analysis

The original study (5) employed database search tools and statistical methods available at the time of publication (2022). To address reviewer concerns and improve protein identification sensitivity, we performed a comprehensive re-analysis using state-of-the-art computational methods including: (1) MSFragger v4.1 (71), a next-generation database search engine with improved sensitivity for modified peptides; (2) Percolator v3.6.5 (72), a machine learning approach for PSM validation; (3) QRILC imputation (73, 74) specifically designed for left-censored mass spectrometry data; and (4) DEqMS (75), which accounts for PSM-count-dependent variance in TMT quantification. This re-analysis recovered substantially more peptide-spectrum matches compared to the original study, enabling more robust statistical analysis of IQDMA treatment effects.

#### 4.15.3 Database search and protein identification

Raw mass spectrometry data files were processed using MSFragger (v4.1) (71) within the FragPipe computational platform (v21.1). Spectra were searched against the UniProt/Swiss-Prot human proteome database including isoforms (downloaded December 2024; 42,421 entries) (76) concatenated with common laboratory contaminants from the cRAP database (175 entries). Reversed protein sequences were appended for target-decoy competition (prefix: rev_), yielding 85,452 total entries.

Database search parameters: precursor mass tolerance ±20 ppm; fragment ion tolerance 0.5 Da (ion trap MS2); fully tryptic specificity with cleavage after K/R except before P; maximum 2 missed cleavages; peptide length 7–50 amino acids; peptide mass 500–5,000 Da. Fixed modifications: carbamidomethylation of cysteine (+57.02146 Da) and TMT11plex labeling of peptide N-termini and lysine (+229.16293 Da). Variable modifications: oxidation of methionine (+15.99490 Da) and deamidation of asparagine/glutamine (+0.98400 Da), with maximum 3 variable modifications per peptide.

Deep learning-based feature prediction was performed using MSBooster (integrated in Frag-Pipe v21.1) to generate predicted retention times and fragment ion intensities, which were used to enhance Percolator scoring. Statistical validation of peptide-spectrum matches (PSMs) was performed using Percolator (v3.6.5) (72), a semi-supervised machine learning approach for discriminating correct from incorrect identifications. PSMs, peptides, and proteins were filtered at 1% FDR. This re-analysis yielded 74,388 PSMs corresponding to 36,496 unique peptides mapping to 6,123 protein groups.

#### 4.15.4 TMT quantification and data processing

TMT reporter ion intensities were extracted from MS3 spectra using pyteomics (v4.6) (77), with reporter ion peaks identified within ±10 ppm of theoretical *m/z* values. Only PSMs passing 1% FDR with non-zero reporter ion intensities in at least 6 of 11 channels were retained. Protein-level abundances were calculated by summing TMT reporter ion intensities across all PSMs mapping to each protein.

Missing values (<3% of the data matrix) were imputed using the QRILC (Quantile Regression Imputation of Left-Censored data) method implemented in the imputeLCMD R package (v2.1) (73, 74). QRILC is designed for mass spectrometry data where missing values result from signals below the detection limit (MNAR, missing not at random). All quantitative data were log_2_-transformed prior to statistical analysis.

Quality assessment confirmed low coefficients of variation within treatment groups (median CV: Vehicle 8.2%, 1 µM 7.9%, 2.5 µM 8.5%, 10 µM 9.1%) and high correlation between biological replicates (*R*^2^ > 0.96). Bovine serum protein contamination was minimal (3 proteins, 0.05% of identified proteins), which were excluded from downstream analyses.

#### 4.15.5 Differential expression analysis

Differential protein expression analysis was performed using DEqMS (v1.18.0) (75), an R/Bioconductor package that extends the limma (78) empirical Bayes framework by incorporating protein-specific variance estimates based on PSM counts. Log_2_-transformed, QRILC-imputed protein abundances were fit to a linear model with a design matrix specifying four treatment groups. Empirical Bayes moderation was applied using robust estimation. Three contrasts were evaluated: 1 µM vs Vehicle, 2.5 µM vs Vehicle, and 10 µM vs Vehicle.

P-values were adjusted for multiple hypothesis testing using the Benjamini-Hochberg procedure (79) to control FDR. Proteins were considered significantly differentially expressed if they met both statistical significance (FDR< 0.05) and biological relevance (|log_2_ fold change| > 0.585, corresponding to 1.5-fold change) criteria.

#### 4.15.6 Effect size calculations

To complement statistical significance testing with measures of practical significance, effect sizes were calculated for all protein abundance changes using both Cohen’s *d* (80) (standardized mean difference) and Hedges’ *g* (81) (bias-corrected for small sample sizes). Cohen’s *d* was first calculated as the mean difference divided by the pooled standard deviation:


$$d=\frac{{\bar{X}}_{1}-{\bar{X}}_{2}}{S_{\text{pooled}}}$$

Hedges’ g was then derived by applying a correction factor *J* to account for positive bias in small samples:


$$g=d\times J,\text{ where }J=1-\frac{3}{4\left(n_{1}+n_{2}\right)-9}$$

**Supplementary Figure S11** presents Cohen’s *d* distributions for visualization, while all statistical interpretations throughout the manuscript use the bias-corrected Hedges’ *g*. Effect sizes were interpreted using adapted thresholds: small ( |g|<0.8), notable ( 0.8≤|g|<1.5), strong ( 1.5≤|g|<3.0), and definitive ( |g|≥3.0).

#### 4.15.7 Gene set enrichment analysis

GSEA was performed using gseapy (v1.1.11) (82) with the preranked algorithm. Proteins were ranked by log_2_ fold change from DEqMS analysis. Enrichment was assessed against MSigDB Hallmark gene sets (2020 version, 50 gene sets) (83), KEGG pathways (2021 Human) (63), and Gene Ontology terms (2023) (84). GSEA parameters: 100 permutations; minimum gene set size 5; maximum gene set size 500; random seed 42. Gene sets with FDR *q*< 0.25 were considered significantly enriched.

#### 4.15.8 Pathway analysis

Pathway-level analysis used curated gene sets from the OmniPath database (85), comprising 13 signaling pathways (262 gene-pathway associations): JAK/STAT (30 genes), PAK/Rho GTPase (25 genes), PI3K/AKT (21 genes), MAPK (17 genes), Apoptosis (27 genes), Cell Cycle (26 genes), Ribosome Biogenesis (15 genes), RNA Processing (17 genes), T-Cell Receptor signaling (19 genes), Nuclear Transport (23 genes), Immune Checkpoint (13 genes), NF-κB (16 genes), and mTOR (13 genes). Pathway enrichment was assessed using Fisher’s exact test with Benjamini-Hochberg correction.

#### 4.15.9 Protein-protein interaction network analysis

Networks were constructed using data from the STRING database (v12) (67), retaining only high-confidence interactions (combined score > 0.7). Networks were analyzed using NetworkX (v3.4.2) (65), calculating node degree, betweenness centrality, and clustering coefficient. Visualization employed Kamada-Kawai force-directed layout, with node sizes scaled by statistical significance and colors indicating direction of change.

#### 4.15.10 Hierarchical clustering and heatmap visualization

Hierarchical clustering was performed using Ward’s minimum variance method (86) with Euclidean distance implemented in scipy.cluster.hierarchy (v1.14.1). Protein abundances were *z*-score normalized per row. Heatmaps were generated using seaborn.clustermap (v0.13.2) (87) with RdBu_r diverging colormap centered at zero, scale ±2 *z*-score units.

#### 4.15.11 Weighted pathway activity scoring

To quantify pathway-level responses to IQDMA treatment, a weighted activity score was calculated integrating three components: (1) pathway position weight reflecting signal propagation (upstream proteins weighted 1.5×, central pathway proteins 1.0×, downstream effectors 0.5×); (2) statistical significance as *−*log_10_(FDR); and (3) effect magnitude as $\left|\log_{2}\text{FC}\right|$. The final protein weight was computed as:


$$W_{i}={\text{Position}}_{i}\times \left(-\log_{10}{\text{FDR}}_{i}\right)\times |\log_{2}{\text{FC}}_{i}|$$

Pathway-level weighted activity was then calculated as the weighted average of individual protein log_2_ fold changes:


$$\text{Weighted Activity}=\frac{\sum_{i=1}^{n}(\log_{2}{\text{FC}}_{i}\times W_{i})}{\sum_{i=1}^{n}W_{i}}$$

This approach prioritizes upstream signaling changes that propagate through pathways while accounting for both statistical confidence and biological effect magnitude.

#### 4.15.12 Alluvial (Sankey) diagram construction

Protein expression fate across IQDMA doses was visualized using alluvial-style flow diagrams constructed with matplotlib (v3.9.2). Proteins were categorized at each dose level (Vehicle, 1 μM, 2.5 μM, 10 μM) into expression states: Upregulated ( $\log_{2}\text{FC}>0.585$), Stable ( $\left|\log_{2}\text{FC}\right|\leq 0.585$), or Downregulated ( $\log_{2}\text{FC}<-0.585$). Flow widths between consecutive dose levels were proportional to the number of proteins transitioning between states, with distinct colors indicating direction of change.

#### 4.15.13 UpSet plot visualization

Set intersections of differentially expressed proteins across treatment conditions were visualized using UpSet plots (88), generated with the upsetplot Python package (v0.9.0). The from_memberships function was used to construct set membership data, with intersection sizes displayed as bar heights and sorted by cardinality (largest intersections first). This visualization enabled identification of dose-specific versus shared protein expression changes.

### 4.16 Kinome-proteomics integration analysis

#### 4.16.1 Multi-omics data integration

To provide an integrated view of IQDMA mechanism of action, kinome screening data (97 kinases) were integrated with quantitative proteomics data (6,123 proteins) from the same cell line (SeAx) and treatment conditions. A union of 28 kinases was established from kinases detected in both datasets. Concordance categories were assigned based on agreement between kinome inhibition and proteomics effect size using Spearman rank correlation and hypergeometric overlap tests.

#### 4.16.2 Rank-Rank Hypergeometric Overlap analysis

RRHO analysis assessed concordance between kinome inhibition rankings and proteomics effect size (|Hedges’ *g*|) across multiple threshold combinations. Hypergeometric tests evaluated overlap significance, with results visualized as concordance heatmaps.

#### 4.16.3 Kinase-substrate network analysis

Kinase-substrate relationships were retrieved from the OmniPath database (85) using the omnipathR package (v3.13.7). For the top 8 kinases with >40% inhibition (ALK 80%, PAK2 69%, JAK3 61%, PIM3 59%, TYK2 59%, BRAF 59%, ABL1 51%, INSR 46%), the five substrates with largest $\left|\log_{2}\text{FC}\right|$ values were identified. Chord diagrams were generated using pycirclize (v1.10.1) (66) with chord thickness proportional to substrate $\left|\log_{2}\text{FC}\right|$.

#### 4.16.4 Concordance classification

Kinases were classified into concordance categories:

- Concordant-High: High inhibition (>30%) and negative Hedges’ g(proteomics downregulation)
- Concordant-Low: Low inhibition (*<*30%) and stable/positive Hedges’ g
- Discordant: Unexpected combinations indicating indirect effects or pathway crosstalk

### 4.17 Statistical analysis

#### 4.17.1 *In vivo* studies

Tumor growth curves were analyzed using unpaired *t*-test or two-way ANOVA, as appropriate, with significance threshold *p* < 0.05. Body weight data were analyzed using two-way ANOVA with *post-hoc* Tukey’s multiple comparison test.

#### 4.17.2 Immunohistochemistry

IHC data were subjected to two-tailed Spearman correlation analysis (89) to evaluate associations between cell densities (CD3^+^, Ki67^+^ cells) and molecular markers (STAT3, STAT5, pY-STAT5). Spearman’s rank correlation coefficient (*r_s_*) with 95% confidence intervals was calculated. Significance threshold: *p<* 0.05.

#### 4.17.3 Proteomics

Statistical analyses were performed using R (v4.4.3) and Python (v3.13). DEqMS was used for differential expression with Benjamini-Hochberg FDR correction (79). Fisher’s exact test was used for pathway enrichment. All analyses used fixed random seeds (seed = 42) for reproducibility.

### 4.18 Software and tools

#### 4.18.1 Mass spectrometry data re-analysis

- FragPipe (v21.1) with MSFragger (v4.1) (71)
- Percolator (v3.6.5) (72)
- pyteomics (v4.6) (77)

#### 4.18.2 Statistical analysis software

- R (v4.4.3) with DEqMS (v1.18.0) (75), limma (v3.60) (78), imputeLCMD (v2.1) (73)
- GraphPad Prism (v10.0) (90)

#### 4.18.3 Python environment

- Python (v3.13) with numpy (v2.2.0), scipy (v1.15.0), pandas (v2.2.0)
- scikit-learn (v1.7.0) for PCA (3D visualization with 95% confidence ellipsoids) and t-SNE (perplexity=5 for *n*= 11 samples, learning_rate=‘auto’, max_iter=1000, random_state=42)
- matplotlib (v3.9.2) and seaborn (v0.13.2) (87)
- gseapy (v1.1.11) (82)
- networkx (v3.4.2) (65)
- pycirclize (v1.10.1) (66)

#### 4.18.4 Image analysis

- QuPath (v0.2.3) (62)
- CaseViewer (3DHistech Ltd.)

#### 4.18.5 Computing environment

All computational analyses were performed on GNU/Linux (Ubuntu 25.10).

### 4.19 Data availability

The mass spectrometry proteomics raw data used in this re-analysis are available from the ProteomeXchange Consortium via the PRIDE partner repository (68) (dataset identifier: PXD035699). The raw data were originally deposited as part of our previous publication (5). Kinome screen data are available in Dataset EV4 of the published EMBO Molecular Medicine manuscript (5). The re-analysis presented here, including database search results, processed quantification matrices, and computational analysis scripts, are available upon reasonable request to the corresponding author.

## Data Availability

The mass spectrometry proteomics raw data used in this re-analysis are available from theProteomeXchange Consortium via the PRIDE partner repository (dataset identifier: PXD035699). Kinomescreen data are available in Dataset EV4 of Sorger et al. (5)EMBO Mol Med (doi: 10.15252/emmm.202115200). Analysis scripts: https://github.com/SaptaDey/IQDMA-STAT5-CDC42-PAK2-CTCL-Repository.
