# Supplementary material for: IQDMA disrupts STAT5 nuclear transport through CDC42-PAK2 axis collapse in cutaneous T-cell lymphoma
Source: Front Immunol. 2026 Mar 17;17:1674527. doi: 10.3389/fimmu.2026.1674527 (PMC13036142; doi:10.3389/fimmu.2026.1674527)
Supplement: Supplementary file 1 [file SupplementaryFile1.pdf]

Supplementary Material

IQDMA disrupts STAT5 nuclear transport through CDC42-PAK2 axis collapse in cutaneous T-cell lymphoma

*Saptaswa Dey, Helena Sorger, Michaela Schlederer, Isabella Perchthaler, Martin L. Metzelder, Lukas Kenner, Richard Moriggl, Peter Wolf**

*Correspondence: Peter Wolf, peter.wolf@medunigraz.at

Contents

#	Figure	Description	Page
1	Figure S1	IQDMA kinome screen: multi-pathway kinase inhibition landscape	2
2	Figure S2	Drug tolerability: organ toxicity and hematological safety profiling	3
3	Figure S3	PUVA vs IQDMA: head-to-head comparison against phototherapy gold standard	4
4	Figure S4	Tumor architecture: H&E histopathology of IQDMA-treated skin lesions	5
5	Figure S5	Proliferative arrest: Ki-67 immunostaining reveals mitotic shutdown	6
6	Figure S6	STAT3 modulation: subcellular redistribution under IQDMA treatment	7
7	Figure S7	Total STAT5 expression: nuclear-cytoplasmic balance in tumor tissue	8
8	Figure S8	pY-STAT5 compartmental shift: nuclear export as therapeutic mechanism	9
9	Figure S9	Proteomics QC: TMT quantification fidelity across 74,388 PSMs	10
10	Figure S10	Sample-level proteomics: PCA clustering and dose-dependent signatures	11
11	Figure S11	Effect size volcano: Cohen's *d* identifies priority drug targets	12
12	Figure S12	Dose-response proteomics: CDC42 as sole statistically significant hit	13
13	Figure S13	Pathway heatmaps: nine signaling cascades under IQDMA perturbation	14
14	Figure S14	Pathway enrichment: weighted activity scores and GSEA rankings	15
15	Figure S15	KEGG network overlays: six pathway maps with effect size annotations	16
16	Figure S16	Functional enrichment: GO network and pathway fold-change distributions	17
17	Figure S17	RRHO concordance: kinome-proteomics rank overlap for 28 kinases	18
18	Figure S18	Kinase-substrate detection: cross-platform concordance analysis	19
19	Figure S19	Substrate enrichment: PAK1 leads with OR = 4.91 among kinase hubs	20
20	Figure S20	Integrated circos: multi-track kinome-proteomics mechanistic summary	21

This document contains 20 supplementary figures (S1–S20) with full legends at original 300 DPI resolution.

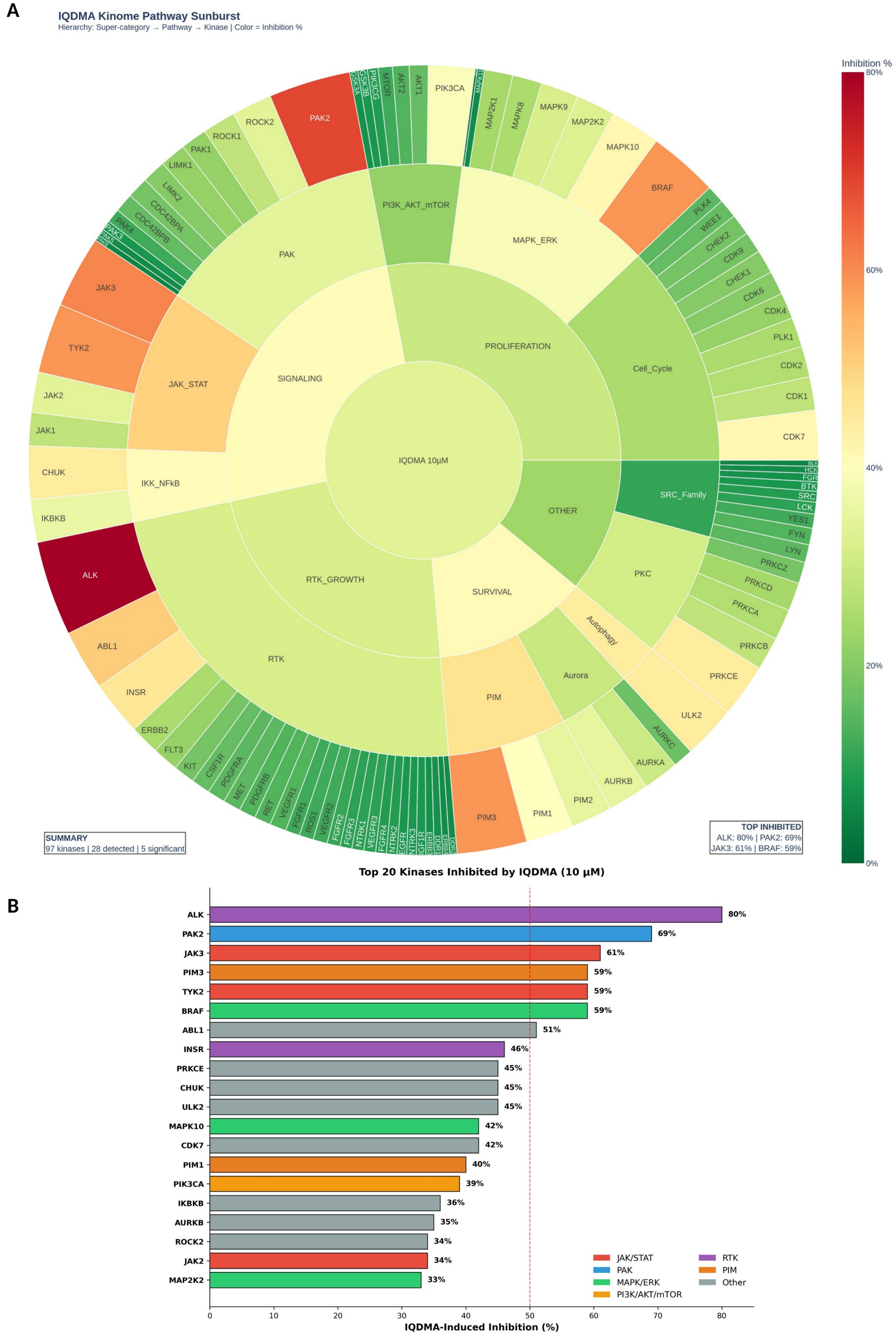

Supplementary Figure S1. IQDMA kinome screen overview. **(A)** Hierarchical sunburst visualization depicting the pathway-kinase organization of IQDMA-induced kinase inhibition. The kinome screen analyzed 97 kinases treated with IQDMA at 10 µM concentration. Top inhibited kinases include ALK (80%), PAK2 (69%), JAK3 (61%), and BRAF (59%). **(B)** Horizontal bar chart of the top 20 kinases ranked by IQDMA-induced inhibition percentage, color-coded by primary signaling pathway membership.

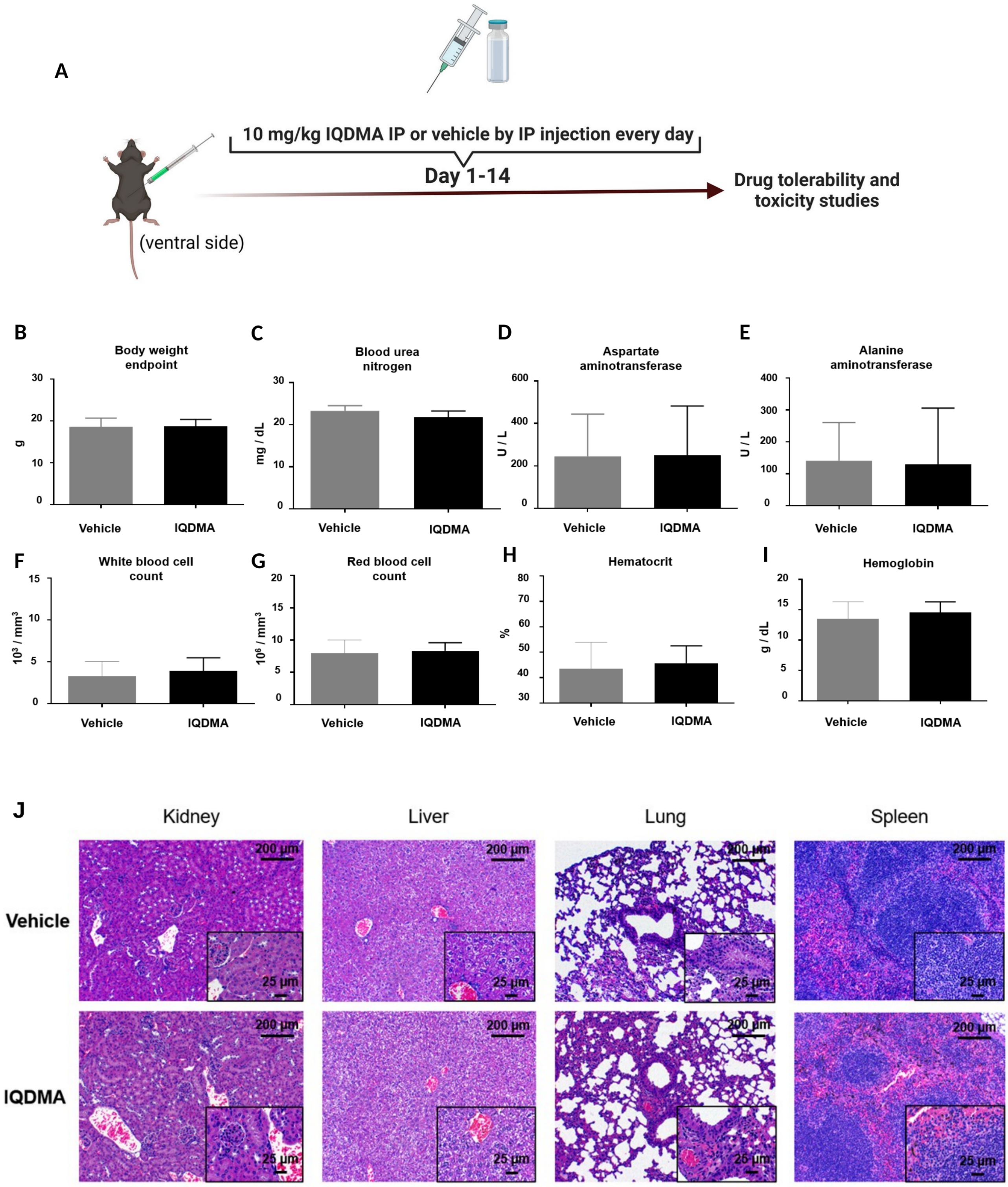

Supplementary Figure S2. Evaluation of tolerability and toxicity of IQDMA in mice following intraperitoneal administration. **(A)** Schematic representation of experimental design. **(B–I)** Assessment of physiological and hematological parameters: **(B)** Body weight, **(C)** Blood urea nitrogen, **(D)** Aspartate aminotransferase, **(E)** Alanine aminotransferase, **(F)** White blood cell count, **(G)** Red blood cell count, **(H)** Hematocrit, and **(I)** Hemoglobin. No significant differences were observed between vehicle and IQDMA-treated groups. **(J)** Histopathological analysis of vital organs including kidney, liver, lung, and spleen.

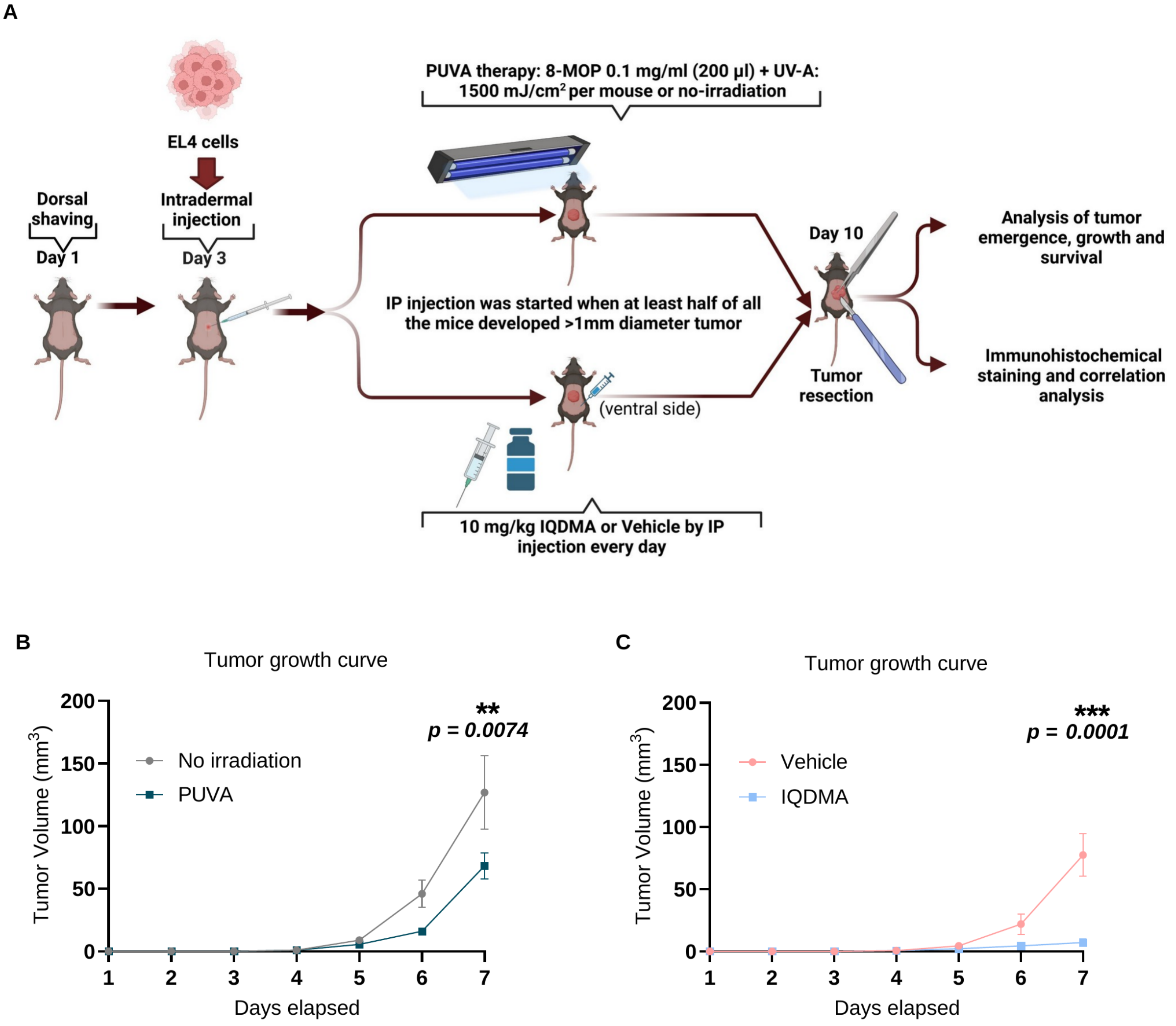

Supplementary Figure S3. Comparative therapeutic efficacy of PUVA and IQDMA in a T-cell lymphoma model. **(A)** Experimental setup starting with EL4 cell injection, then PUVA therapy or IQDMA treatment, and subsequent tumor resection and analysis. **(B)** Tumor volume comparison over time shows PUVA treatment (dark blue line) vs no irradiation (gray line) ($P = 0.0074$). **(C)** Tumor volume analysis between IQDMA-treated mice (light red line) and vehicle control (light blue line) ($P = 0.0001$). *Note: This figure was previously Main Figure 2 and has been moved to Supplementary Materials.*

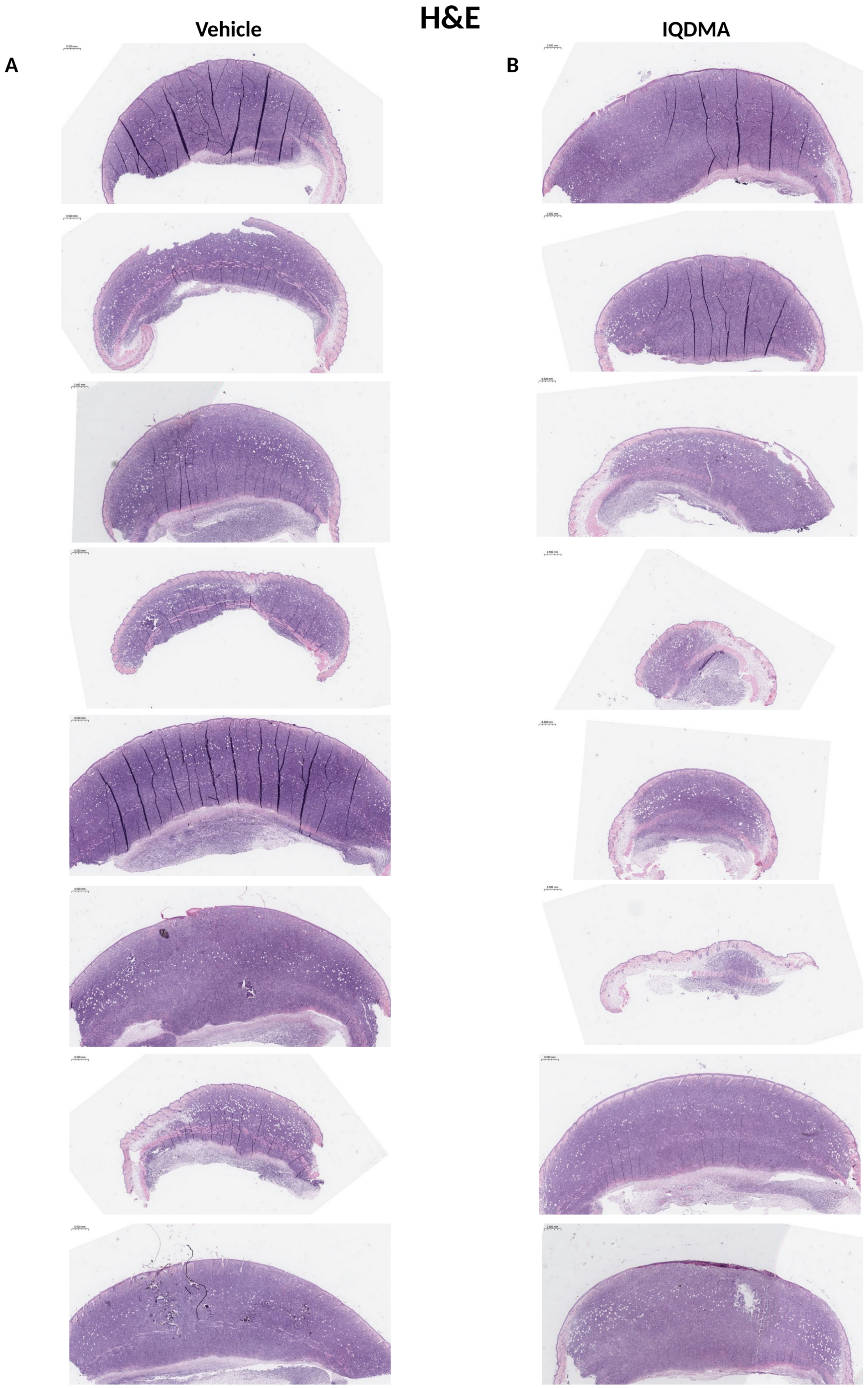

Supplementary Figure S4. Histological examination of skin tumor tissues from a tumor-stage Mycosis Fungoides mouse model following IQDMA treatment. **(A–B)** Representative H&E-stained sections of skin tumor tissues from mice treated with **(A)** vehicle or **(B)** 10 mg/kg IQDMA.

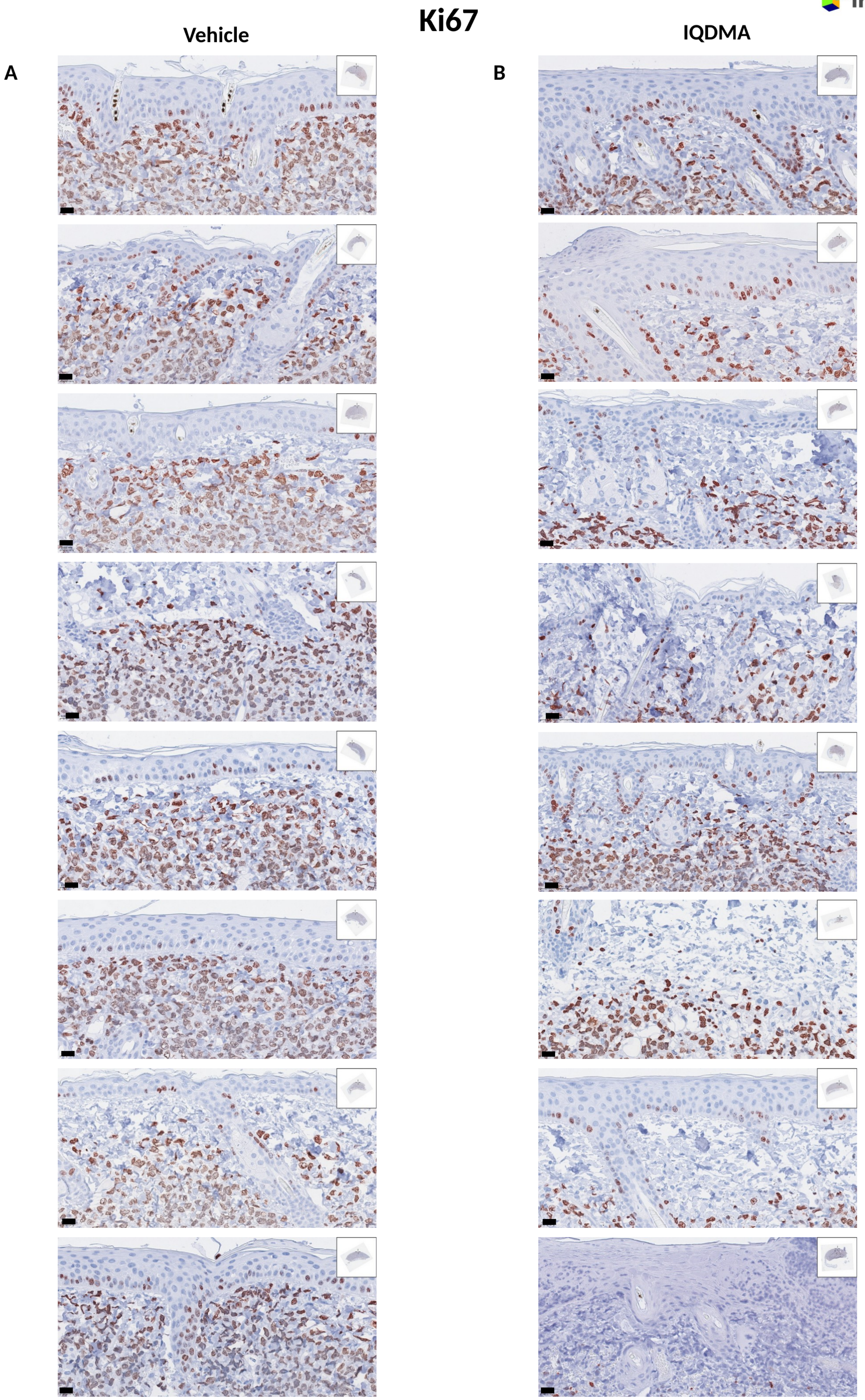

Supplementary Figure S5. Immunohistochemical analysis of Ki-67 expression in skin tumor tissues. **(A–B)** Representative images of Ki-67 staining in **(A)** vehicle-treated and **(B)** IQDMA-treated mice. IQDMA treatment reduces Ki-67-positive cells, indicating decreased tumor cell proliferation.

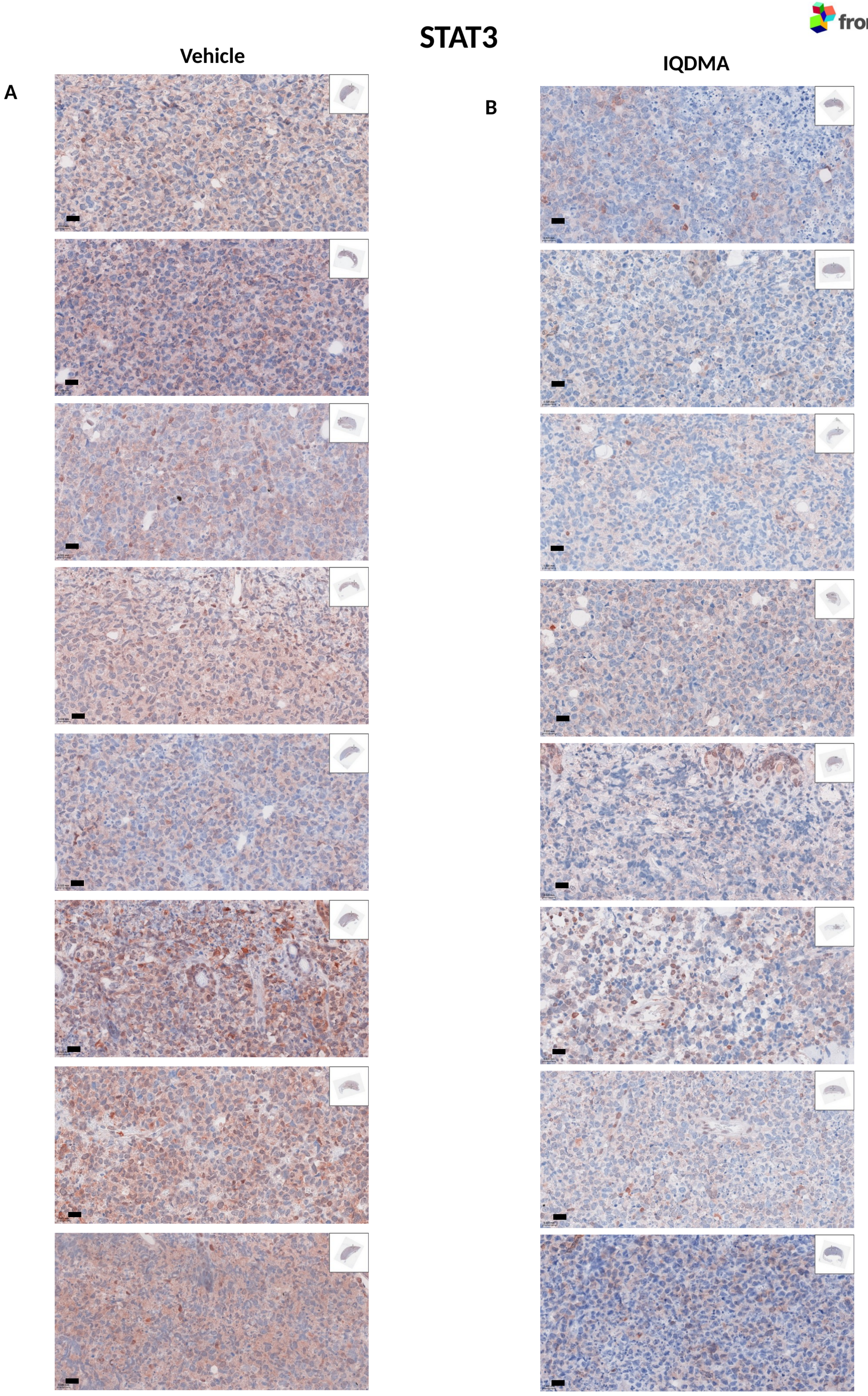

Supplementary Figure S6. Immunohistochemical analysis of STAT3 expression in skin tumor tissues. **(A–B)** Representative images of STAT3 staining in **(A)** vehicle-treated and **(B)** IQDMA-treated mice. IQDMA treatment modulates STAT3 expression and subcellular distribution.

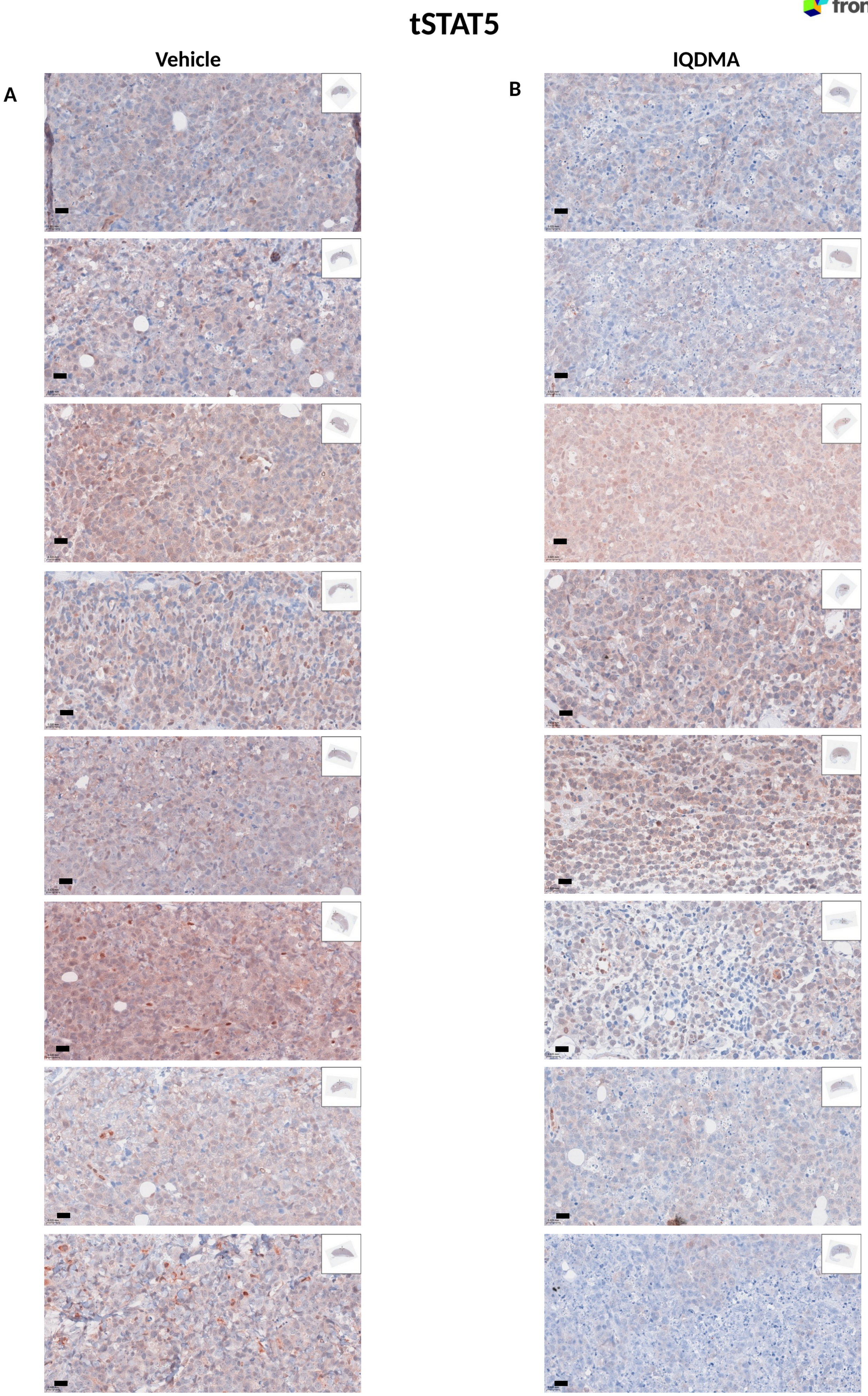

Supplementary Figure S7. Immunohistochemical analysis of total STAT5 (tSTAT5) expression in skin tumor tissues. **(A–B)** Representative images of total STAT5 staining in **(A)** vehicle-treated and **(B)** IQDMA-treated mice.

frontiers

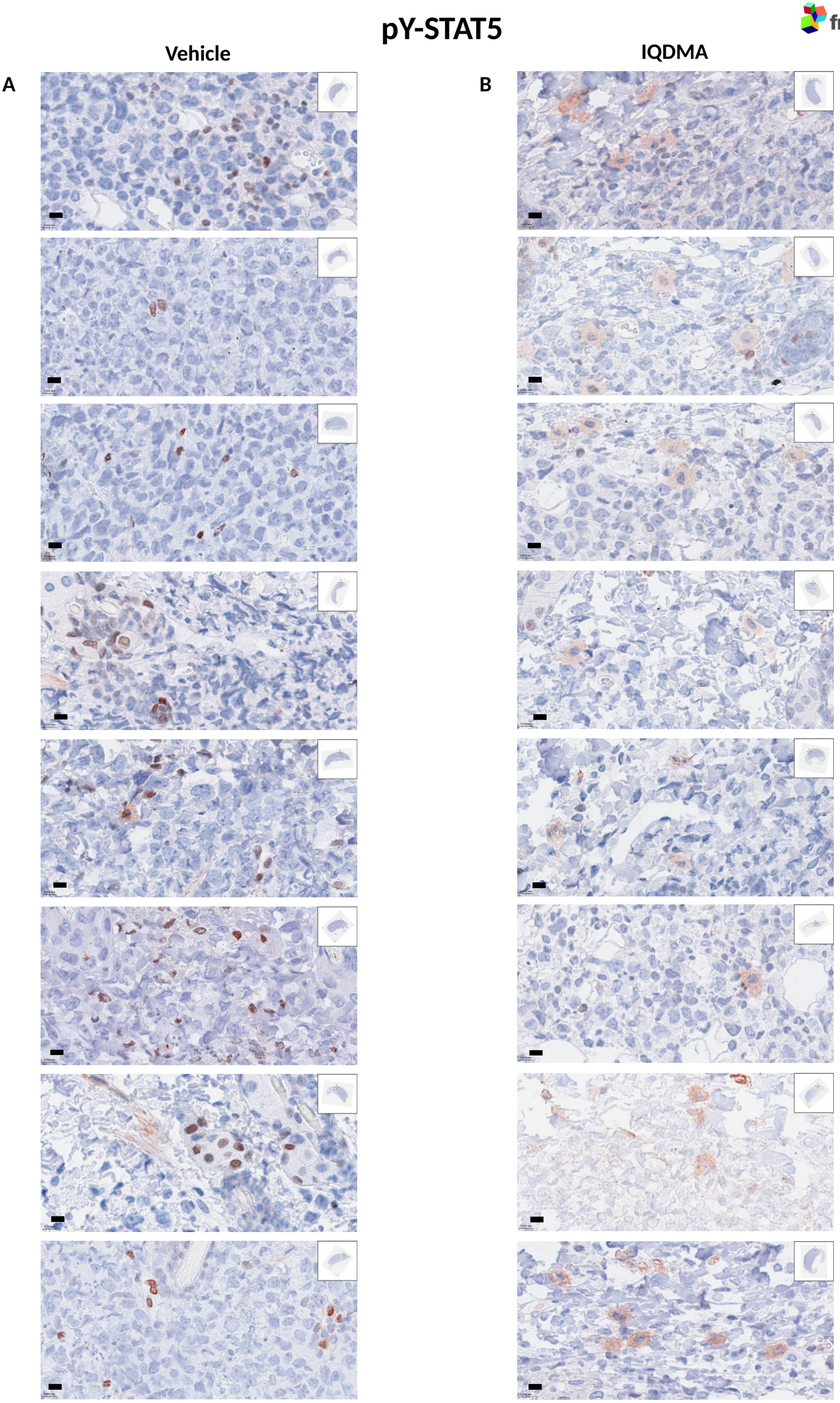

Supplementary Figure S8. Immunohistochemical analysis of phosphorylated STAT5 (pY-STAT5) expression in skin tumor tissues. (A–B) Representative images of pY-STAT5 (Tyr694) staining in **(A)** vehicle-treated and **(B)** IQDMA-treated mice. A compartmental shift of pY-STAT5 from the nucleus to the cytoplasm was observed in the IQDMA-treated group.

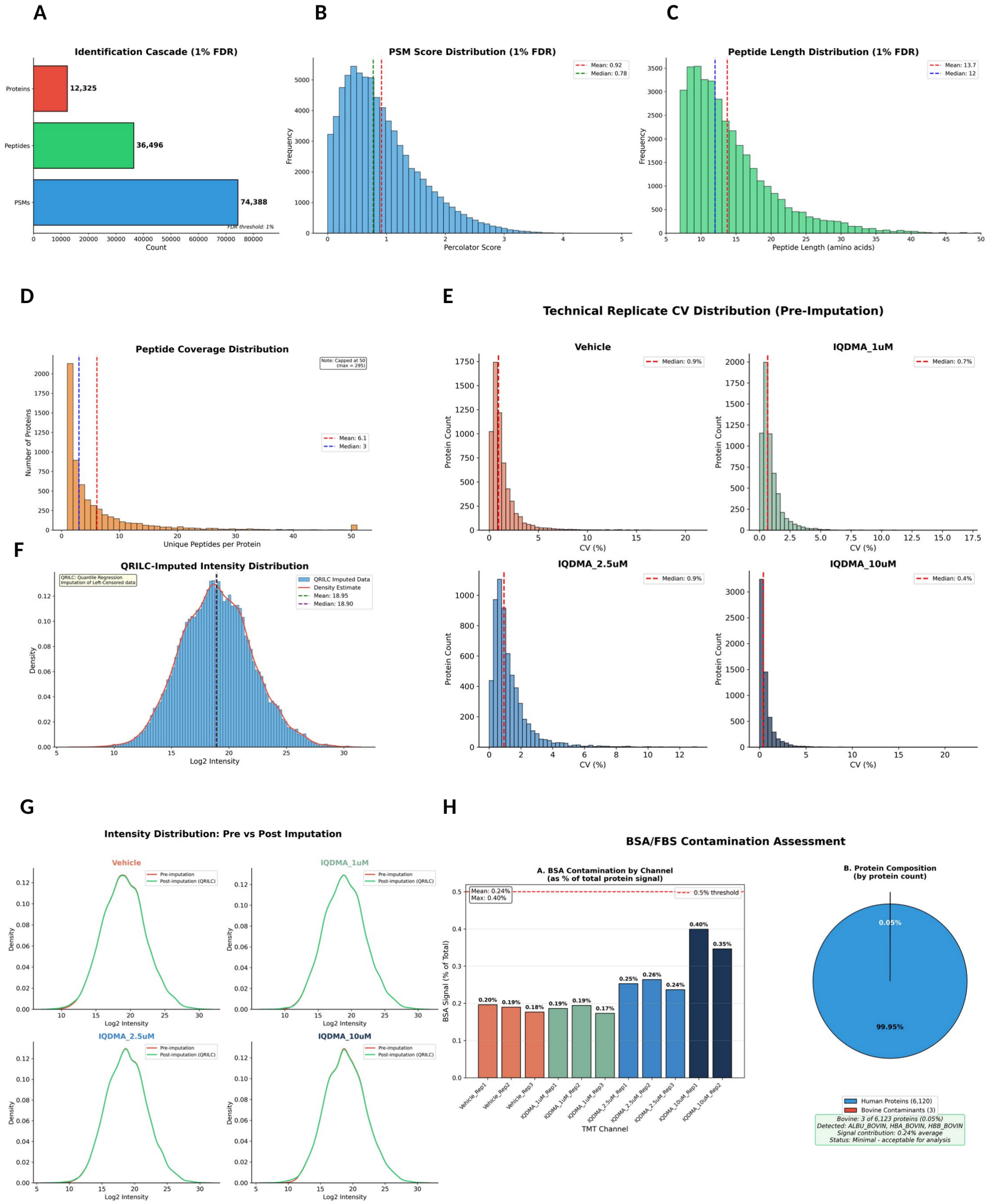

Supplementary Figure S9. Quality control metrics and data processing for quantitative TMT proteomics. **(A)** Identification cascade showing sequential filtering at 1% FDR: PSMs (*n* = 74,388), unique peptides (*n* = 36,496), and protein groups (*n* = 6,123). **(B)** Distribution of Percolator discriminant scores. **(C)** Peptide length distribution. **(D)** Coefficient of variation (CV) distribution for TMT reporter ion intensities. **(E)** Distribution of unique peptides per protein. **(F)** Distribution of protein abundances following QRILC imputation. **(G)** Comparison of protein intensity distributions before and after QRILC imputation. **(H)** Assessment of bovine serum albumin (BSA) contamination: 0.05% bovine proteins detected.

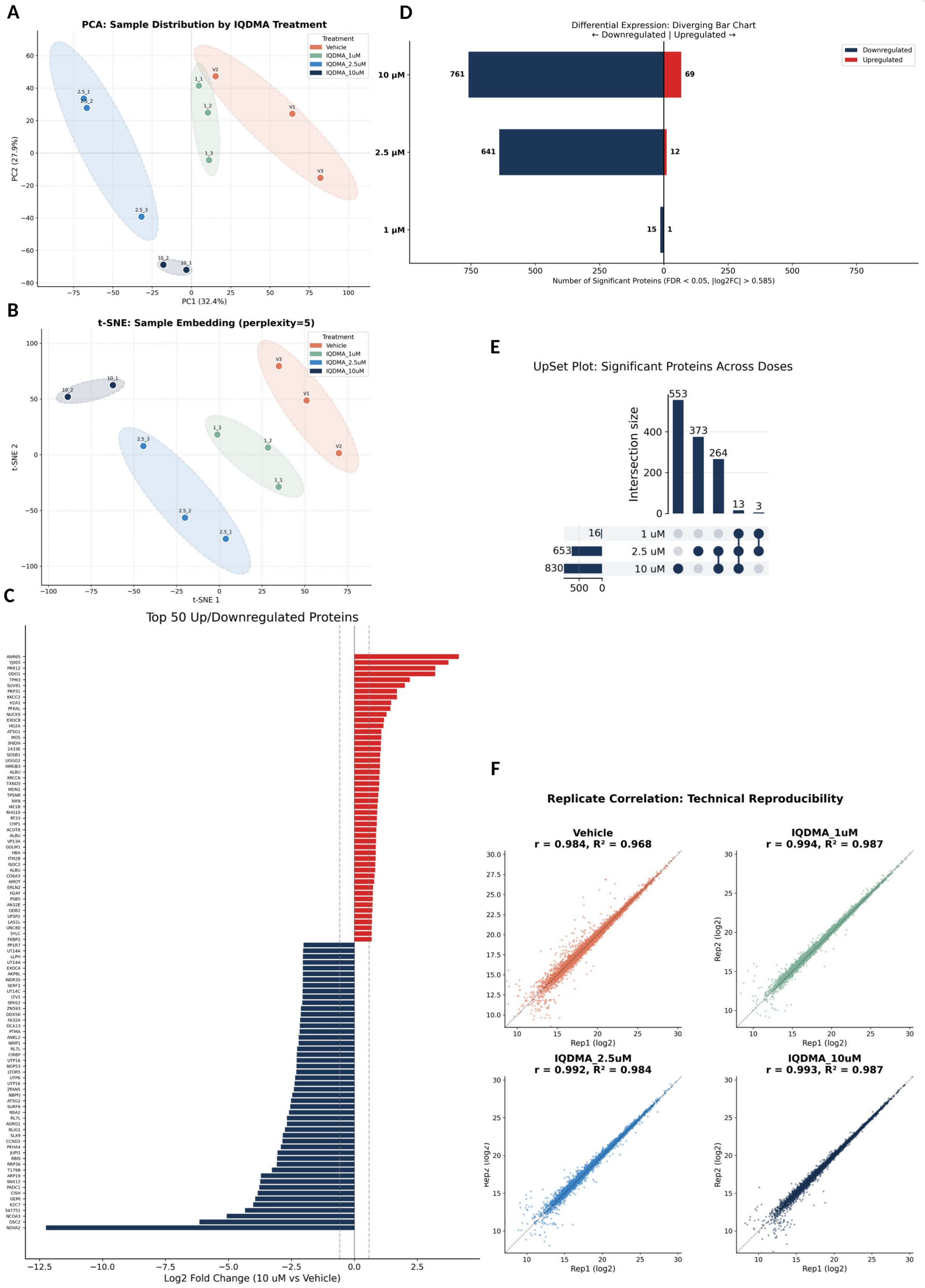

Supplementary Figure S10. Sample-level analysis and differential protein expression. **(A)** Principal component analysis (PCA) of protein abundances. **(B)** Summary of significantly altered proteins at each IQDMA concentration. **(C)** t-SNE visualization of sample relationships. **(D)** UpSet plot showing overlap of significantly altered proteins across doses. **(E)** Waterfall plot of top differentially expressed proteins at 10 µM IQDMA. **(F)** Replicate correlation scatter plots demonstrating high technical reproducibility ($R^2 > 0.96$).

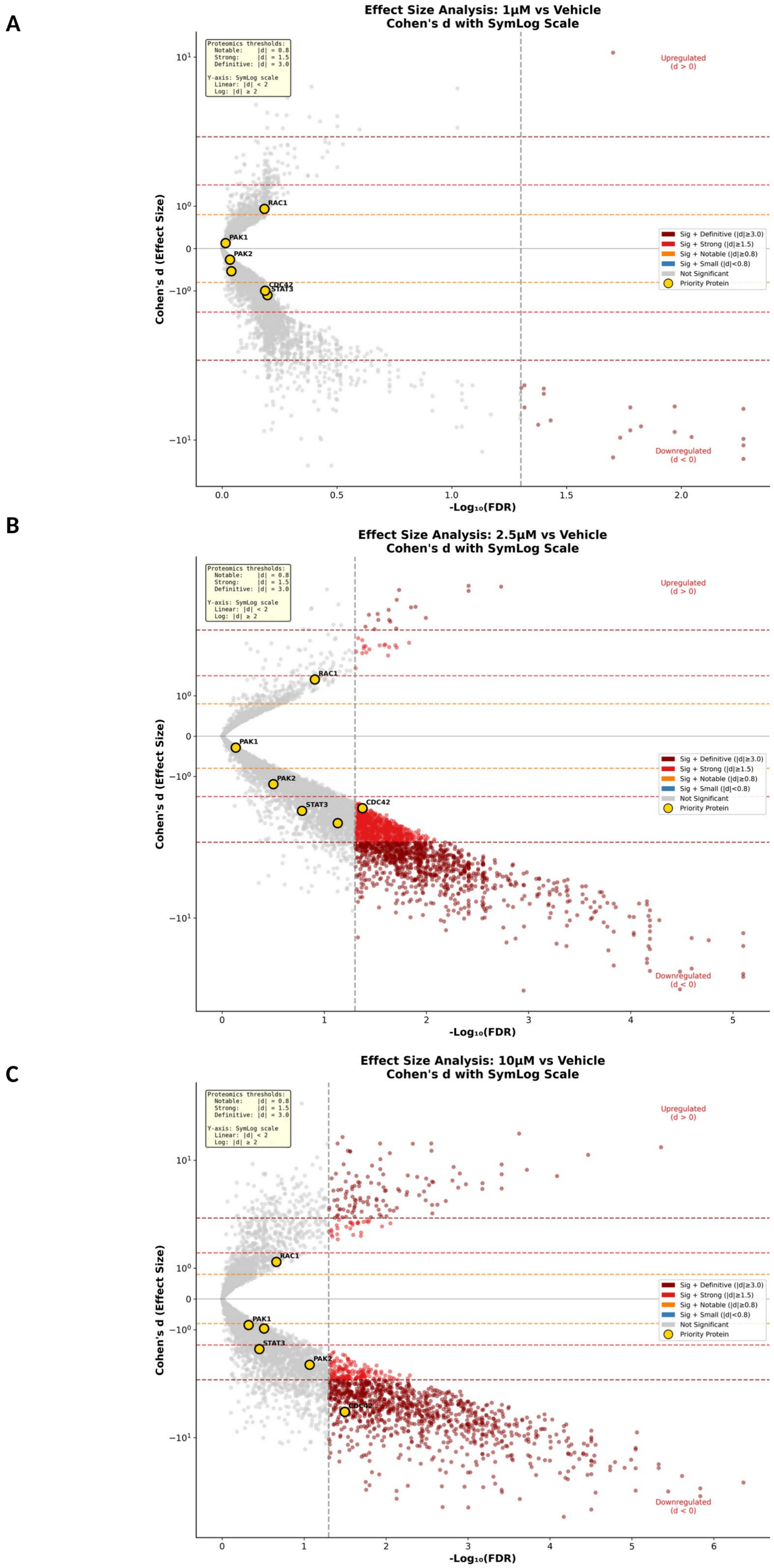

Supplementary Figure S11. Effect size analysis of IQDMA-induced proteomic changes. **(A–C)** Cohen's *d* effect size versus statistical significance at **(A)** 1 μM, **(B)** 2.5 μM, and **(C)** 10 μM IQDMA. Priority proteins (STAT3, STAT5A, STAT5B, JAK1, PAK1, PAK2, CDC42, RAC1) are highlighted.

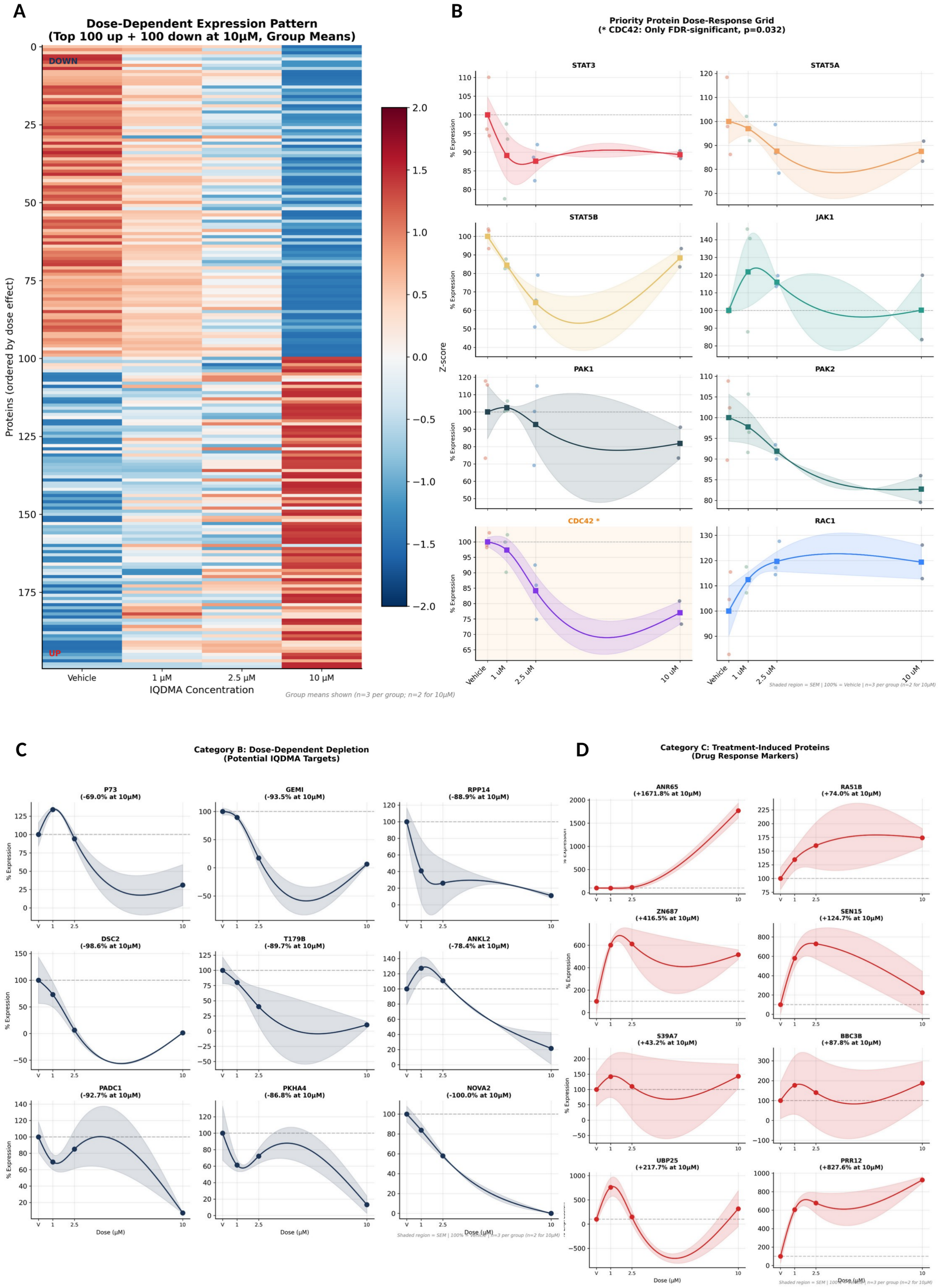

Supplementary Figure S12. Dose-response characterization of IQDMA effects on the proteome. **(A)** Heatmap of dose-dependent protein expression changes for the top 200 dose-responsive proteins. **(B)** Dose-response curves for priority target proteins. CDC42 panel is highlighted as the only priority protein reaching statistical significance (FDR = 0.032 at 10 μM). **(C)** Expression profiles of Category C (treatment-induced) proteins. **(D)** Expression profiles of Category B (dose-depleted) proteins.

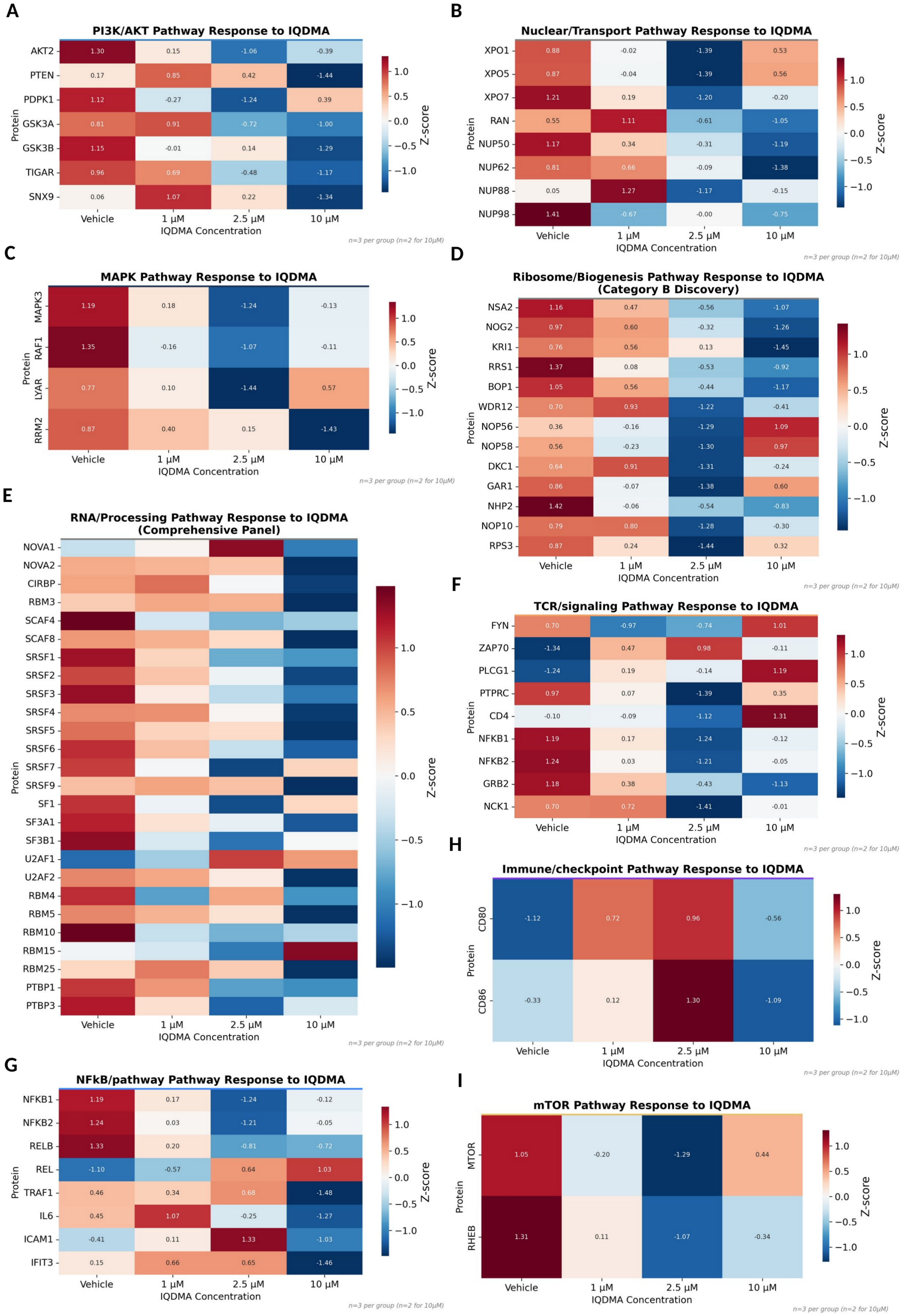

Supplementary Figure S13. Pathway-specific protein expression heatmaps. **(A)** PI3K/AKT signaling pathway. **(B)** Nuclear transport machinery. **(C)** MAPK signaling cascade. **(D)** Ribosome biogenesis. **(E)** RNA processing machinery. **(F)** T-cell receptor (TCR) signaling. **(G)** Immune checkpoint regulators. **(H)** NF-κB signaling pathway. **(I)** mTOR signaling pathway.

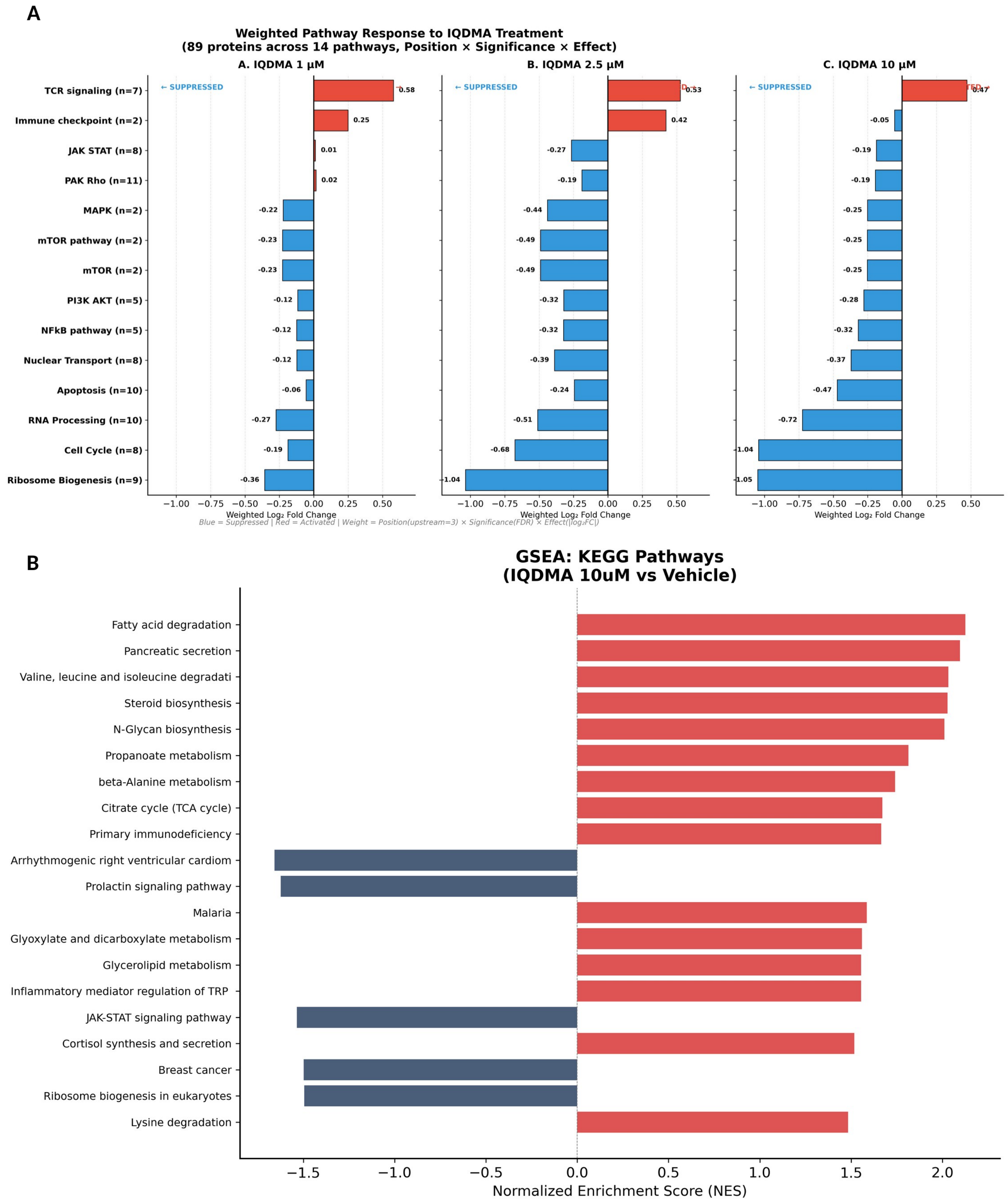

Supplementary Figure S14. Pathway enrichment analysis summary. **(A)** Weighted pathway activity scores across IQDMA doses. **(B)** Gene Set Enrichment Analysis (GSEA) of KEGG pathways showing normalized enrichment scores for the top 20 pathways.

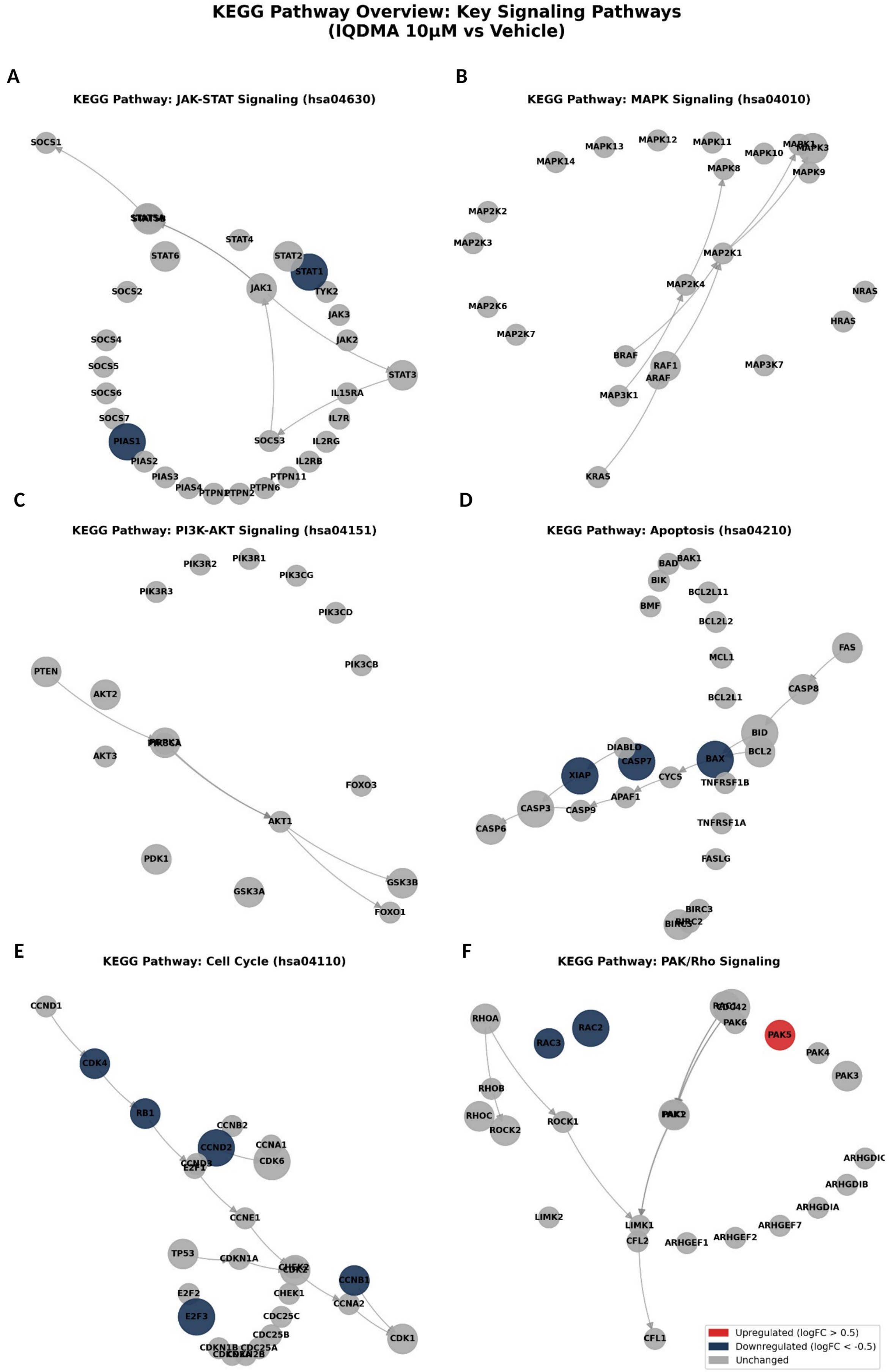

Supplementary Figure S15. KEGG Pathway Network Visualizations. Network representations of key signaling pathways with IQDMA response overlay. **(A)** KEGG Pathway: JAK-STAT Signaling (hsa04630) showing pathway members with effect sizes and FDR significance annotations. **(B)** KEGG Pathway: MAPK Signaling (hsa04010) displaying MAPK cascade components. **(C)** KEGG Pathway: PI3K-AKT Signaling (hsa04151) showing survival pathway network. **(D)** KEGG Pathway: Apoptosis (hsa04210) displaying apoptotic regulators. **(E)** KEGG Pathway: Cell Cycle (hsa04110) showing cell cycle regulatory network. **(F)** KEGG Pathway: PAK/Rho Signaling showing Rho-GTPase and PAK family network. Node colors represent effect size direction and magnitude.

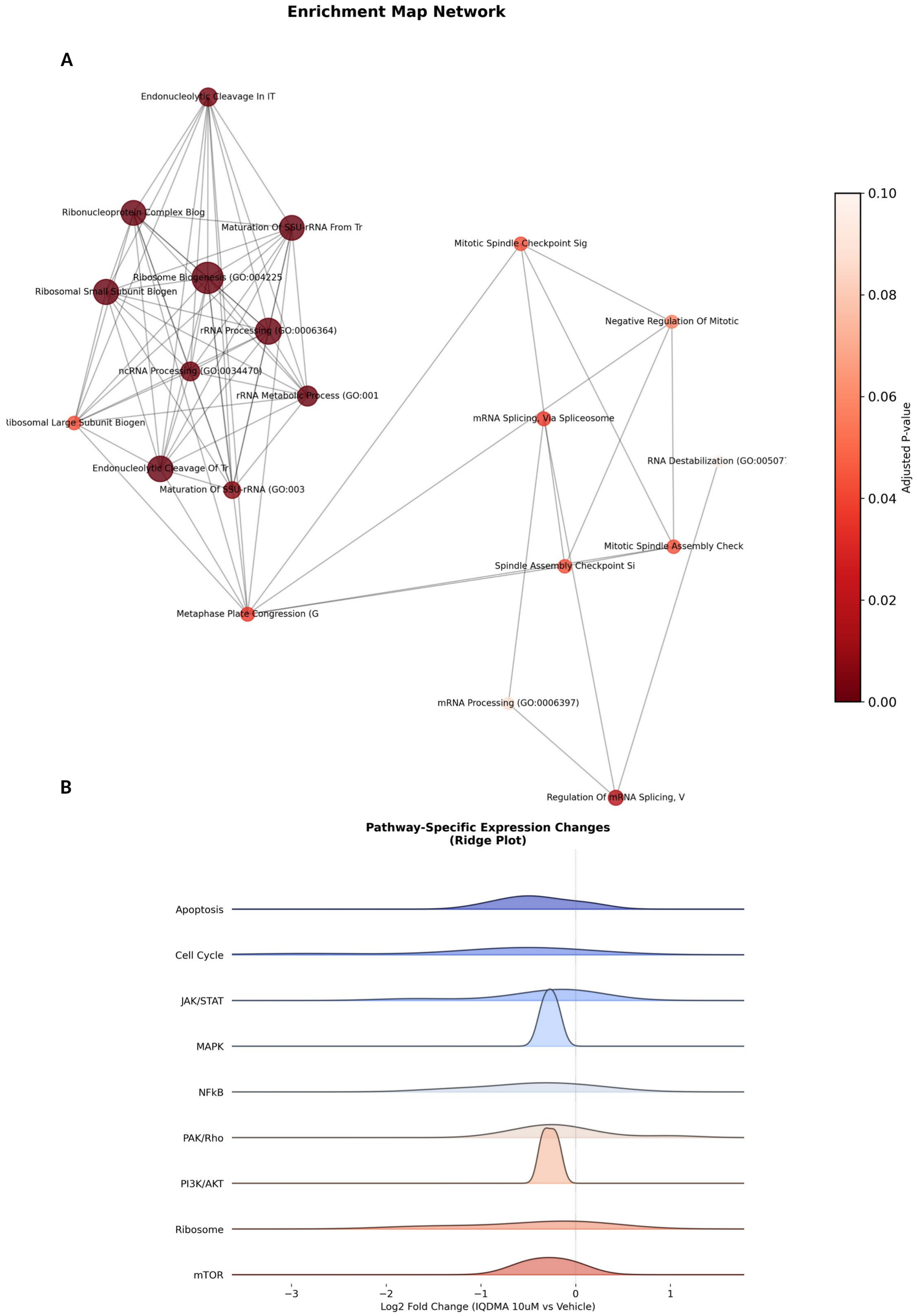

Supplementary Figure S16. Functional enrichment network and pathway expression distributions. **(A)** Gene Ontology enrichment network for downregulated proteins. Nodes represent enriched GO Biological Process terms. **(B)** Ridge density plot of pathway-specific fold change distributions across 9 major signaling pathways.

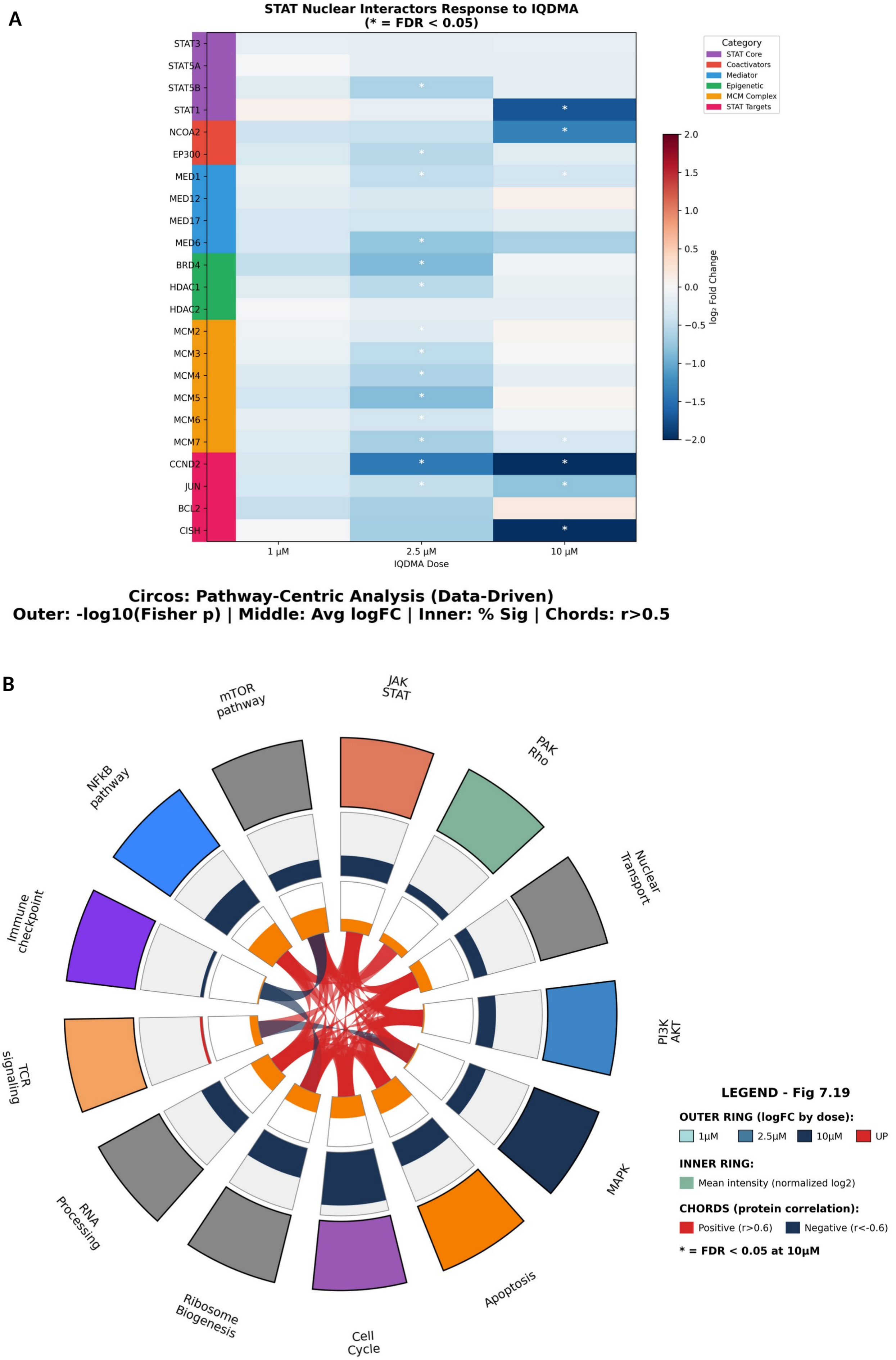

Supplementary Figure S17. Rank-Rank Hypergeometric Overlap (RRHO) analysis of kinome-proteomics concordance. **(A)** RRHO heatmap visualizing overlap between kinome inhibition rankings and proteomics effect size rankings for 28 kinases. **(B)** Concordance scatter plot of kinome inhibition versus proteomics effect size. Spearman ρ = −0.260 (P = 0.182).

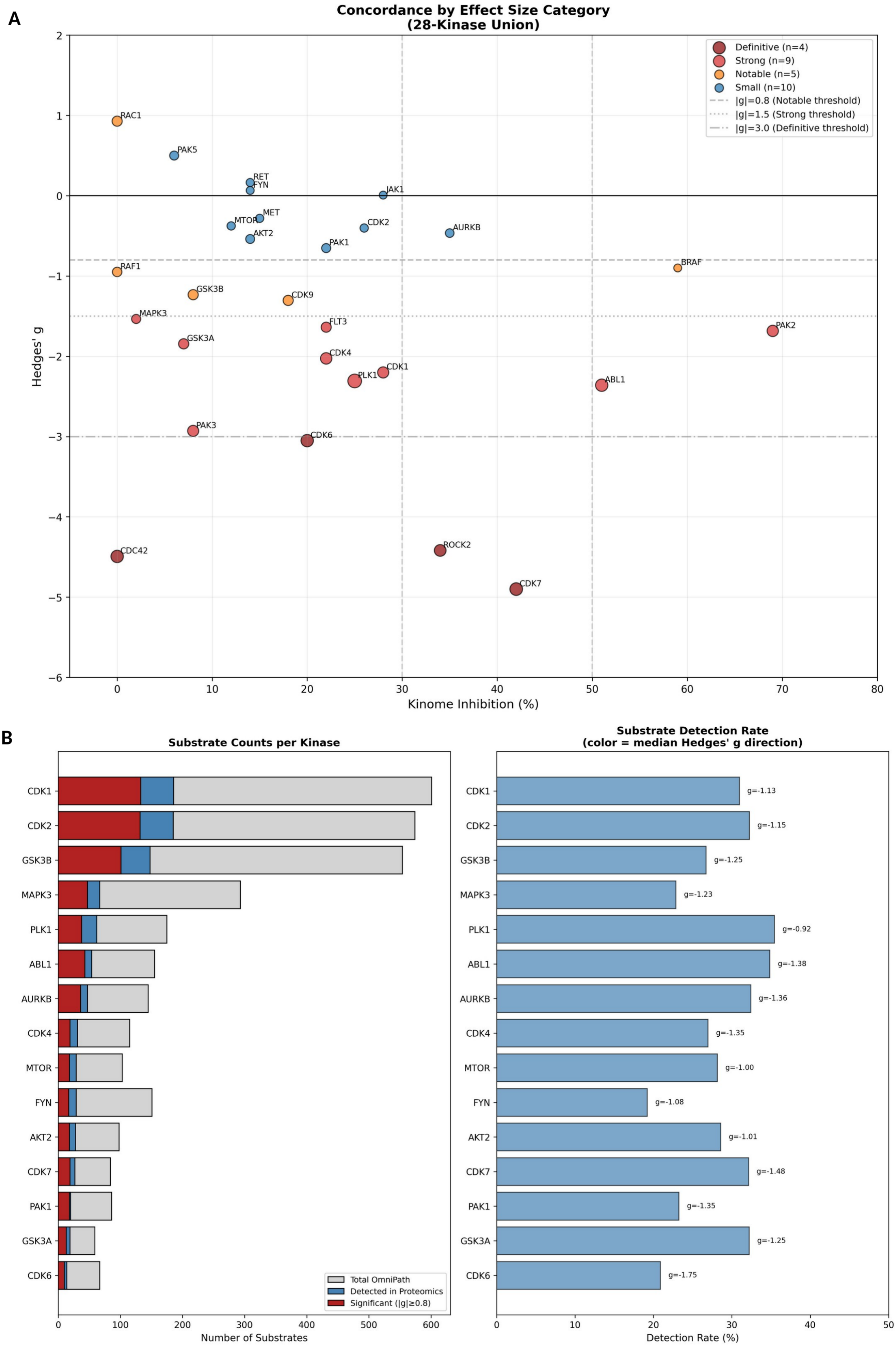

Supplementary Figure S18. Kinome-proteomics concordance analysis. **(A)** Effect category scatter plot showing relationship between kinome inhibition and proteomics effect size magnitude for 28 detected kinases. **(B)** Pathway enrichment analysis bar chart showing substrate counts and detection rates for top 15 kinases.

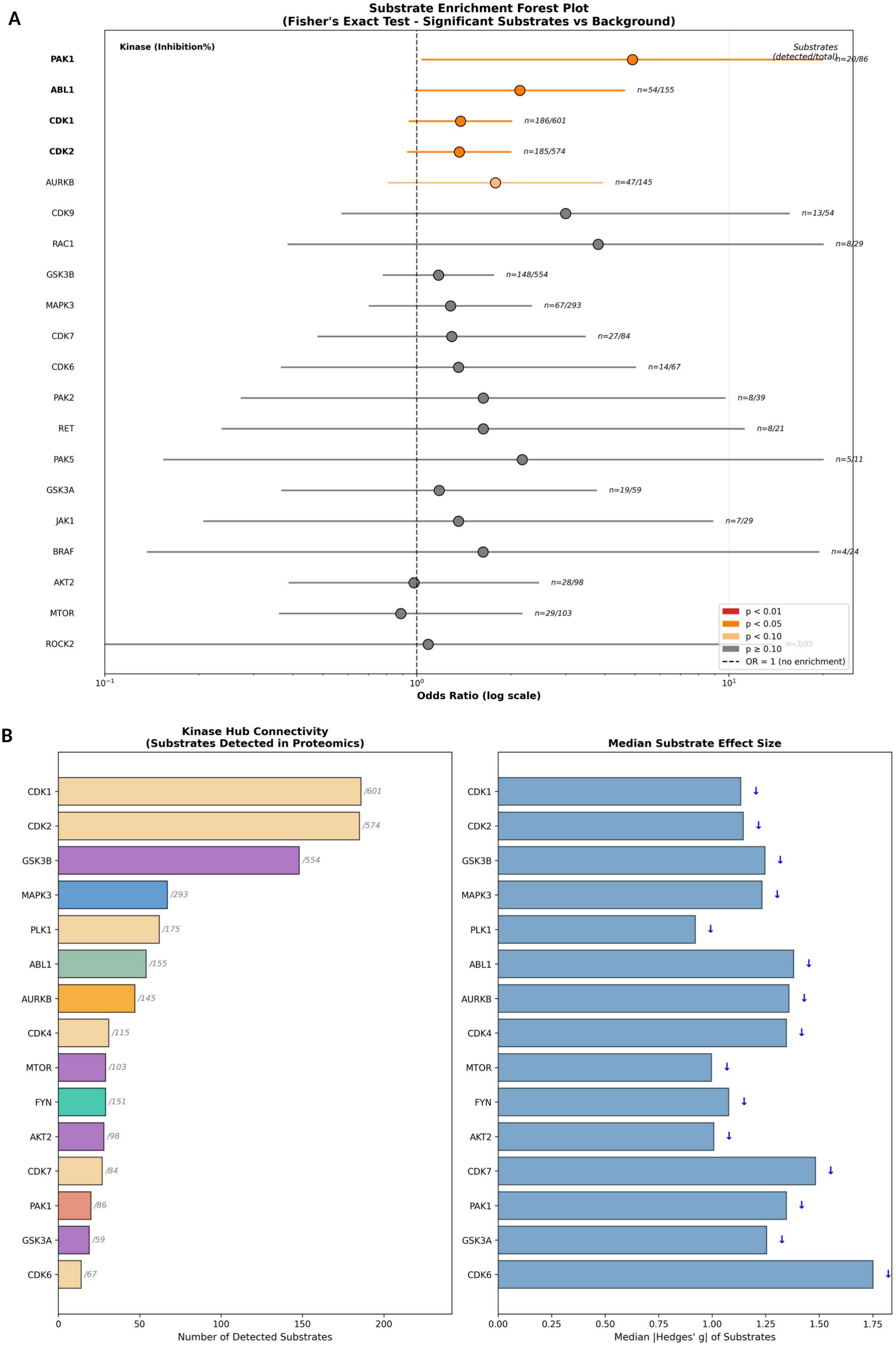

Supplementary Figure S19. Effect size forest plot and kinase hub analysis. **(A)** Forest plot of substrate enrichment odds ratios. Significantly enriched kinases: PAK1 (OR = 4.91, P = 0.011), ABL1 (OR = 2.14, P = 0.013), CDK1 (OR = 1.38, P = 0.029), CDK2 (OR = 1.37, P = 0.032). **(B)** Kinase hub analysis scatter plot showing detected substrate counts and median absolute Hedges' *g*.

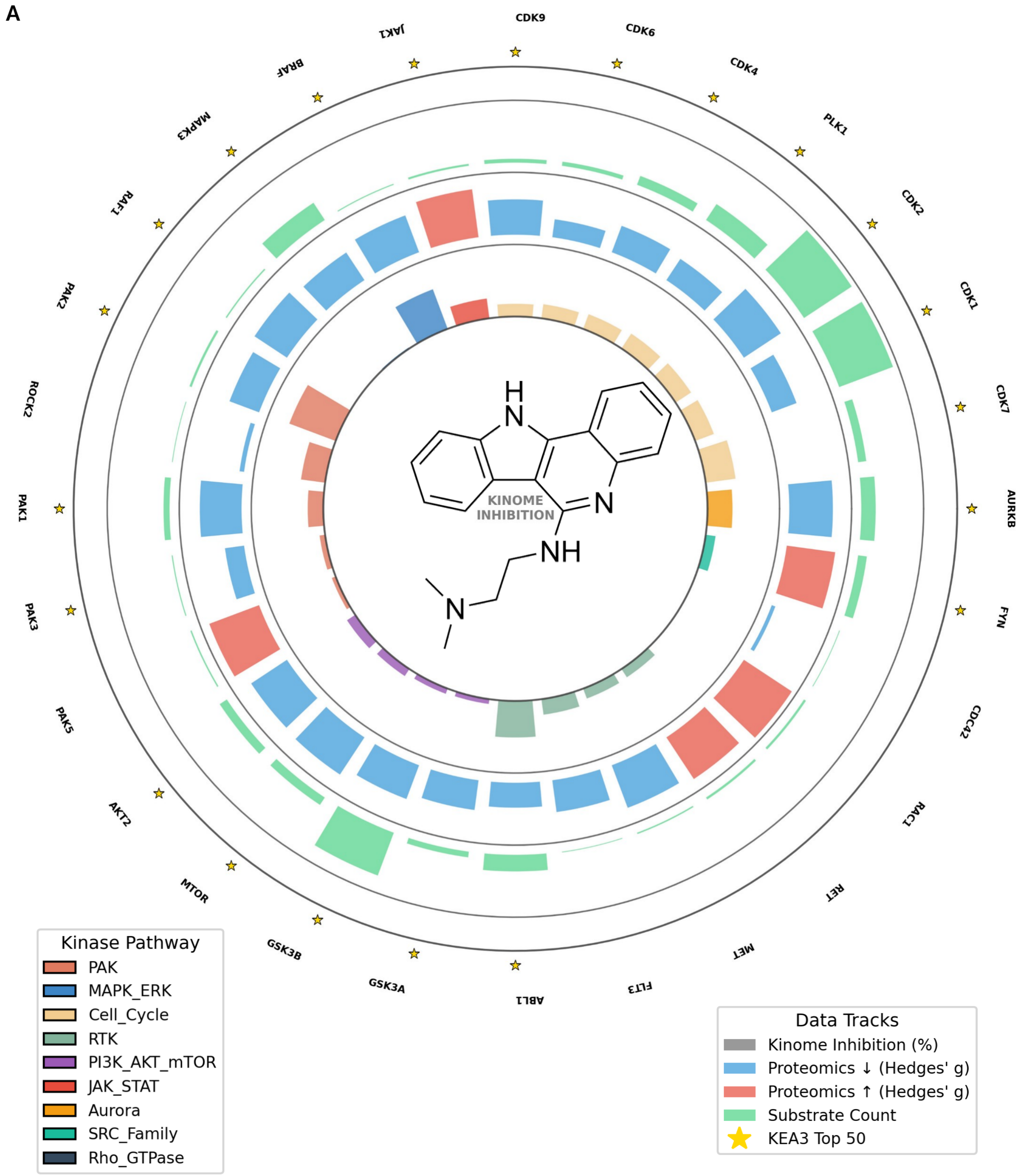

Supplementary Figure S20. Integrated Kinome-Proteomics Summary. **(A)** Multi-track circos displaying integrated kinome-proteomics analysis with IQDMA chemical structure in center. Tracks from outer to inner: (1) Kinase names organized by pathway; (2) Kinome inhibition percentage; (3) Proteomics $\log_2$ fold change; (4) $-\log_{10}$(FDR) significance; (5) Chords connecting kinases to detected substrates. This integrated visualization summarizes IQDMA's multi-kinase targeting profile and downstream proteomic consequences. PAK2 and ROCK2 are highlighted as key storyline kinases.
